# New monstersaur specimens from the Kaiparowits Formation of Utah reveal unexpected richness of large-bodied lizards in Late Cretaceous North America

**DOI:** 10.1098/rsos.250435

**Published:** 2025-06-18

**Authors:** C. Henrik Woolley, Joseph J. W. Sertich, Keegan M. Melstrom, Randall B. Irmis, Nathan D. Smith

**Affiliations:** ^1^ Dinosaur Institute, Natural History Museum of Los Angeles County, Los Angeles, CA, USA; ^2^ Department of Earth Sciences, University of Southern California, Los Angeles, CA, USA; ^3^ Department of Geosciences, Warner College of Natural Resources, Colorado State University, Fort Collins, CO, USA; ^4^ Smithsonian Tropical Research Institute, Panama, Panama; ^5^ Natural History Museum of Utah, University of Utah, Salt Lake City, UT, USA; ^6^ Department of Biology, University of Central Oklahoma, Edmond, OK, USA; ^7^ Department of Geology and Geophysics, University of Utah, Salt Lake City, UT, USA

**Keywords:** Monstersauria, Squamata, Laramidia, Grand Staircase–Escalante National Monument, biogeography

## Abstract

Monstersauria (Squamata, Anguimorpha) fossils are present in most Upper Cretaceous sedimentary basins in western North America, but despite almost a century of collection, their record remains extremely fragmentary. Here, we describe new material belonging to large-bodied monstersaurs, including a new taxon, *Bolg amondol* gen. et sp. nov., based on a fragmentary associated skeleton and co-occurring specimens from the middle unit of the upper Campanian Kaiparowits Formation of Grand Staircase–Escalante National Monument in southern Utah, USA. Phylogenetic analyses recover *B. amondol* within Monstersauria, with two unique anatomical features: fused osteoderms on the jugal and the presence of autotomy septa on the distal caudal vertebrae. Critically, *B. amondol* is morphologically distinct from the problematic Late Cretaceous North American monstersaur *Palaeosaniwa canadensis*, whereas co-occurring monstersaur vertebrae and parietals from the Kaiparowits Formation (cf. *P. canadensis*) highlight a pressing need for a reassessment of this important, widespread taxon. These results offer new evidence that at least three lineages of distinct, large-bodied monstersaurian lizard were present on the palaeolandmass of Laramidia during the Campanian Stage. Importantly, *B. amondol* represents the most complete squamate recovered from late Campanian southern Laramidia and reveals key anatomical characteristics for future identification of isolated lizard fossil elements.

## 1. Introduction

Monstersauria [1] is a long-lived, extant (Cretaceous–Present) clade of Northern Hemisphere anguimorph lizards, with taxa distributed across North America and Eurasia [1–22]. The oldest fossils attributed to Monstersauria are found in Lower Cretaceous sediments in Japan (*Morohasaurus kamitakiensis*, lower Albian Ohyamashimo Formation [15]) and the western United States (*Primaderma nessovi*, Cenomanian Mussentuchit Member, Cedar Mountain Formation [10]). Today, Monstersauria is represented by five species belonging to the genus *Heloderma* that are distributed throughout the deserts of the American Southwest and northwestern Mexico [23]. Because of the complex history of squamate phylogenetics and the presence of multiple competing hypotheses of squamate evolutionary relationships based on morphology and genomics, monstersaurian taxa have been placed in various combinations of anguimorph clades [11,12,24–29]. In some morphology-based hypotheses, Monstersauria is a polyphyletic group that includes taxa deemed to be more closely related to *Varanus* than to anguids [11,25]. Recent molecular and combined-evidence phylogenetic analyses [12,24,26–29] recovered Monstersauria/Helodermatidae (taxa more closely related to *Heloderma* than *Varanus* or *Anguis*) as sharing a more recent common ancestor with Anguidae than with Varanidae. Despite the disagreement over outgroup relationships, the monophyly of Monstersauria has been recovered repeatedly with new analyses and the addition of new taxa (e.g. [11–14]; this analysis).

Our current understanding of the clade Monstersauria contains fewer than 15 described fossil and extant species [6,12,23,30]. Despite this limited diversity, they attained an array of body sizes and morphological disparity throughout the clade’s evolutionary history. The most recent diagnosis for Monstersauria [6] lists a combination of 34 unambiguous synapomorphies that distinguish the clade from other anguimorphans (electronic supplementary material). Combinations of these character-states give the clade its distinctive general body plan: bulky, osteoderm-covered lizards with blunt snouts and widely spaced, spire-like conical teeth.

Across the approximately 100 Myr fossil record of Monstersauria, the clade achieved its highest taxonomic diversity and widest geographic distribution during the Campanian Stage (approx. 83.6−72.1 Ma) of the Late Cretaceous Period, with occurrences of taxa in present-day Mongolia [1,2,5,6,9,12], and in sedimentary basins in the Western Interior of North America [3,4,7,8,30–34]. The Campanian Djadokhta and Baruungoyot formations of Mongolia preserve the most complete and best-studied fossil monstersaurians: the smaller-bodied *Gobiderma pulchrum* [2,12] and the larger-bodied *Estesia mongoliensis* [1,5,6]. These two taxa are part of an assemblage of at least 50 co-occurring species of lizards [2,9], illustrating the richest-known squamate diversity in a Mesozoic faunal assemblage [35]. Additionally, the potential monstersaur taxon (see §5) *Asprosaurus bibongriensis* is known from an incomplete, associated skeleton from the Campanian Seonso Formation of South Korea [14], and could represent the largest member of the clade yet known.

The exceptionally complete Campanian monstersaur fossils from Asia contrast with those of North America, which are represented almost exclusively by numerous but fragmentary specimens [3,4,7,8,30–34,36]. Only two monstersaurian species have thus far been erected from the late Campanian of North America: *Palaeosaniwa canadensis* [31] and *Labrodioctes montanaensis*, the latter assigned to Helodermatidae [7]. Other large-bodied anguimorph taxa include *Parasaniwa cynochoros* [32] from the Kaiparowits Formation of Utah, as well as the tentative referral of specimens from the Oldman Formation (Alberta, Canada) to a new species of *Parasaniwa* (*Parasaniwa*, new species, cf. *P. wyomingensis* [7]), referred to herein as Oldman Fm *Parasaniwa* sp. nov. To date, most described and catalogued fossil remains of material exhibiting monstersaurian and/or varanoid/platynotan features from the late Campanian of North America have either been referred to *Pal. canadensis* (usually material belonging to larger individuals) or to *Paras. wyomingensis* (usually material belonging to smaller individuals). These historical referrals span northern (Wapiti Formation [30]; Oldman/Dinosaur Park formations [7]; Judith River Formation [37]; Two Medicine Formation [8,30]; central (Mesaverde Formation [38]), and southern geologic units from the Western Interior of North America (Aguja Formation [34]; Cerro del Pueblo Formation [39]) that comprise more than 5 million years of the Campanian Stage. The referral of fragmentary Campanian lizard material to *Pal. canadensis*, a taxon erected on the basis of a fragmentary vertebra from the Oldman Formation [31], and *Paras. wyomingensis*, a taxon erected on the basis of material from the upper Maastrichtian Lance Formation [31], over such a wide palaeolatitudinal (approx. 35−60° N) and temporal range suggests that the taxonomic diversity of ‘platynotans’/monstersaurians on the late Campanian palaeolandmass Laramidia [40] has been historically underestimated.

Over the past 30 years, survey efforts in the upper Campanian Kaiparowits Formation of Grand Staircase–Escalante National Monument, southern Utah, USA, have recovered an abundant and diverse squamate assemblage [32,41,42]. This assemblage contains taxa found nowhere else on Laramidia, bolstering dinosaur-based [43] and plant-based [44] hypotheses of sedimentary basin-level, or regional, endemism during the Campanian Stage. Despite intensified research and the abundance of squamate fossils, relatively few specimens have been referred to monstersaurian taxa and/or other clades that may include monstersaurian taxa under different hypotheses of squamate evolutionary relationships (i.e. Platynota, Varanoidea). Nydam [32] assigned UMNH VP 21180, a partial maxilla, to a new species of *Parasaniwa*, *Paras. cynochoros*. Nydam [32] also described several isolated marginal tooth-bearing elements and teeth that exhibit morphology distinctive enough to assign to different ‘morphotypes’ of playnotan lizards (Kaiparowits morphotypes H–J). Though fragmentary, the fossils described in Nydam [32] demonstrate the presence of at least two large-bodied anguimorph taxa in the Kaiparowits Formation.

Here, we describe several new specimens from the Kaiparowits Formation that can be attributed to large monstersaur lizard taxa, including one associated partial skeleton assigned to a new taxon, *Bolg amondol* gen. et sp. nov. Using descriptive comparisons and the results of parsimony-based phylogenetic analyses, we discuss the implications of this new material for understanding monstersaur taxonomic richness, morphological disparity and body-size distribution during the late Campanian of Laramidia.

### 1.1. Geologic setting

**Figure 1.**
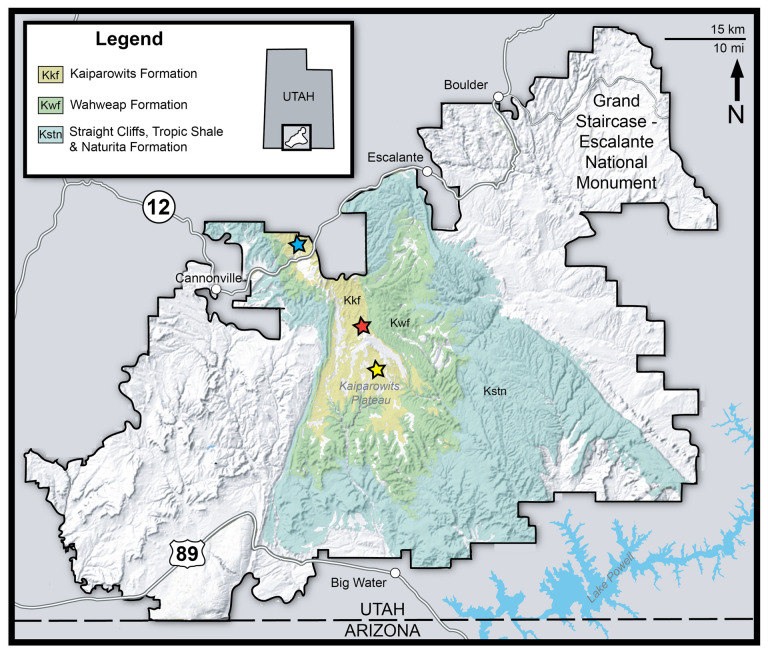
Geologic map of Upper Cretaceous sediments in Grand Staircase–Escalante National Monument, Utah, modified from Titus *et al*. [45]. Stars indicate field areas containing localities of specimens described in this study (blue star: The Blues field area; red star: Fossil Ridge field area). Yellow star indicates the approximate location of the *Parasaniwa cynochoros* [32] type locality. Shaded relief layer of Grand Staircase–Escalante National Monument contains information from *TessaDEM*, which is made available here under the Open Database License (OdbL): https://tessadem.com*.*

The Kaiparowits Formation is exposed in south-central Utah, USA, almost exclusively within the boundaries of Grand Staircase–Escalante National Monument (figure 1). Topographically, the Kaiparowits Formation (approximately 1000 m thick) is characterized by badland-forming, bluish-grey siltstones and mudstones, and greyish sandstone exposures, which overlay the predominantly yellow-brownish fluvial channel deposits that comprise the lower-middle Campanian Wahweap Formation [45–53]. The Kaiparowits Formation was deposited in an alluvial to coastal plain setting, characterized by predominantly fluvial and floodplain deposits that include crevasse splays, perennial ponds and oxbow lakes [47,49,53]. Source material probably originated from the west along the Sevier Orogenic Belt [54,55] and was transported eastward to the Western Interior Seaway. The palaeoenvironment was probably seasonally flooded and ponded based on paludal deposits, biotic indicators [44,56], leaf physiognomy [44] and the isotopic composition of dinosaur teeth [57–59]. Temperature estimates and palaeoenvironmental interpretations [54,60,61] suggest that the climate of the Kaiparowits Formation was humid and seasonally tropical/subtropical, similar to the present-day Gulf Coast of the United States.

**Figure 2.**
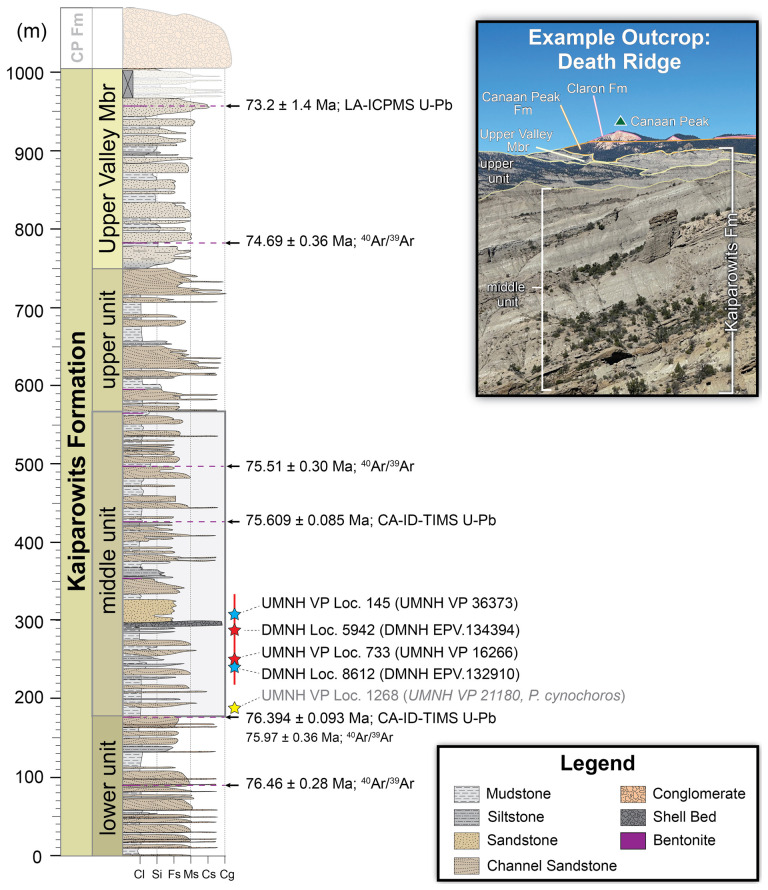
Composite stratigraphic column of the Kaiparowits Formation, with stratigraphic positions of localities of described material marked by stars (blue star: The Blues field area; red star: Fossil Ridge field area). Vertical red line indicates the stratigraphic uncertainty range for these localities. Yellow star indicates the position of the *Parasaniwa cynochoros* [32] type locality. Black horizontal arrows indicate stratigraphic position of radiometrically dated bentonite layers, indicated in purple. Stratigraphic column modified from Roberts [49], Beveridge *et al*. [53] and Ramezani *et al*. [62].

Currently, the Kaiparowits Formation is informally divided into upper, middle and lower units [47,49,51] (figure 2), as well as the stratigraphically highest and formalized Upper Valley Member [53,62], with the middle unit producing the bulk of floral and faunal fossil specimens [63]. All localities preserving materials described within this study are from the middle unit. Specific locality information for all localities in this study is reposited at the Natural History Museum of Utah (UMNH) and Denver Museum of Nature & Science (DMNH), respectively, and made available to qualified researchers and officials upon request and review.

**Figure 3.**
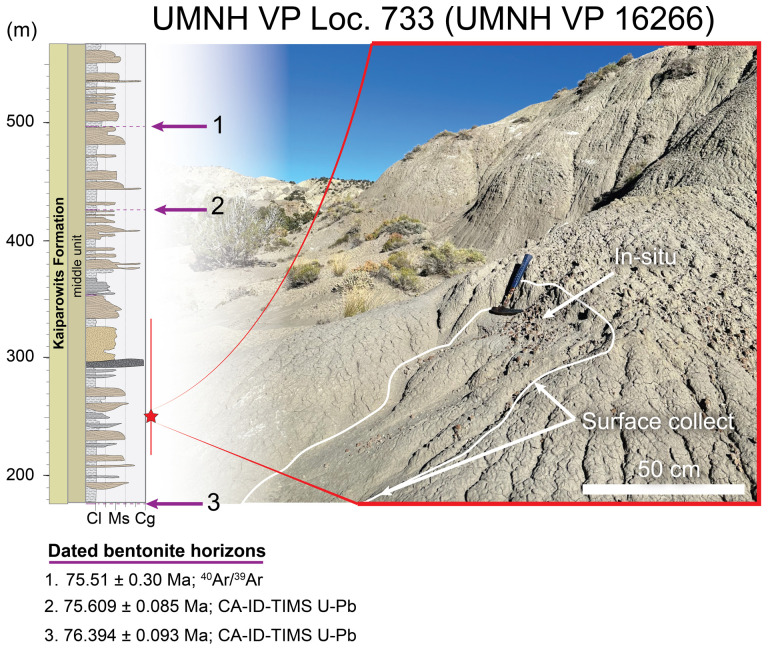
Fossil locality UMNH VP Loc. 733, where the holotype specimen of *Bolg amondol* (UMNH VP 16266) was discovered in 2005. Photo taken by author C.H.W. in October 2023.

More specifically, all specimens were recovered from the lower half of the middle unit of the upper Campanian Kaiparowits Formation (figure 2), below bentonite horizon KBC-109 (U-Pb CA-ID-TIMS dated to 75.609 ± 0.085 Ma [62]; and above bentonite horizon KP-07 (U-Pb CA-ID-TIMS dated to 76.394 ± 0.093 Ma [62]). The holotype specimen of *Bolg amondol* gen. et sp. nov. (UMNH VP 16266) was collected from a grey-green horizon (figure 3), consisting of massively bedded, muddy silt to fine sand. This lithology corresponds to lithofacies Sf of facies association FA8 of Roberts [49], interpreted as rapid deposition in a floodplain environment.

## 2. Material and methods

### 2.1. Field collections and permits

All specimens described here were collected under palaeontological research and collection permits issued by the United States Bureau of Land Management (BLM) Utah State Office and under the supervision of the Grand Staircase–Escalante National Monument and Paria River District Office. Specimen UMNH VP 16266 was discovered *in situ* by author J.J.W.S. in 2005 in the Fossil Ridge field area and collected under BLM surface permit UT-S-04-016; additional surface elements were recovered by authors C.H.W., J.J.W.S. and R.B.I. in 2023 under surface permit UT09-008S-GS. Specimen DMNH EPV.132910 was discovered by G. Maccracken in 2018 at an extensive microvertebrate locality in The Blues field area, collected under BLM surface permit UT11-010S-GS. Specimens DMNH EPV.134394.1 and EPV.134394.2 were excavated from a large, ceratopsid-dominated bonebed locality in The Blues field area, discovered by author J.J.W.S. in 2018 and A. Patterson in 2021, and collected under BLM excavation permit UT16-009E-GS.

### 2.2. Specimen preparation

UMNH VP 16266 was collected from matrix using hand tools and mechanically prepared using airscribes. Surface specimens were assembled by hand using reversable adhesives B15 and B72 in acetone in the fossil preparation laboratories at the Natural History Museum of Utah (UMNH VP 16266 and VP 36373), Denver Museum of Nature & Science (DMNH EPV.132910, EPV.134394.1 and EPV.134394.2), and Natural History Museum of Los Angeles County (UMNH VP 16266).

### 2.3. Imaging and processing

Skull and girdle elements of UMNH VP 16266 were imaged using a Keyence VHX-5000 digital microscope in the Natural History Museum of Los Angeles County’s Ichthyology collections. All other images of specimens were taken using a Cannon EOS 5D with a Cannon EFS 35 mm macro lens. Stacked images were taken using the software program Helicon Remote, which were then exported for processing in the software program Helicon Focus. Stacked photos were edited in Adobe Photoshop CC 2023 and figures for the manuscript were assembled in Adobe Illustrator CC 2023.

### 2.4. Computed tomography

UMNH VP 16266, DMNH EPV.132910 and DMNH EPV.134394.1 were scanned using a GE Phoenix Nanotom CT scanner at the University of Southern California Molecular Imaging Center. Additionally, the pectoral girdle elements and metapodial of UMNH VP 16266 and DMNH EPV.134394.2 were scanned using a Skyscan 1273 µCT scanner in the Ichthyology collections at the Natural History Museum of Los Angeles County. The specimens were scanned using a 0.1 mm copper filter at a voltage of 80 kV, and a voxel size of 42.22 µm. CT image data was processed and segmented using Avizo Lite (v. 2019.2), and surface *.stl files of all specimens were exported for imaging in MeshLab (v. 2022.02).

### 2.5. Institutional abbreviations

ANSP: Academy of Natural Sciences, Philadelphia, Pennsylvania, United States of America; BMNH: Natural History Museum, London, United Kingdom; CMNFV: Canadian Museum of Nature, Ottawa, Ontario, Canada; DMNH: Denver Museum of Nature & Science, Denver, Colorado, United States of America; FMNH: Field Museum of Natural History, Chicago, Illinois, United States of America; GSC: Geological Survey of Canada, Ottawa, Ontario, Canada; IGM: Mongolian Institute of Geology, Ulaan Baatar, Mongolia; KDRC: Korea Dinosaur Research Center, Chonnam National University, Republic of Korea; MOR: Museum of the Rockies, Bozeman, Montana, United States of America; MNHAH: Museum of Nature and Human Activities, Hyogo, Japan; NHMG: Guangxi Natural History Museum, Zoology Collection, Nanning, Guangxi Province, China; NHMLAC/LACM: Natural History Museum of Los Angeles County, Los Angeles, California, United States of America; OMNH: Sam Noble Museum of Natural History, Norman, Oklahoma, United States of America; SDSNH: San Diego Natural History Museum, San Diego, California, United States of America; TMM: University of Texas Jackson School Museum of Earth History, Austin Texas, United States of America; TMP: Royal Tyrell Museum of Paleontology, Drumheller, Alberta, Canada; UALVP: Laboratory for Vertebrate Paleontology, University of Alberta, Edmonton, Alberta, Canada; UCMP: University of California Museum of Paleontology, Berkeley, California, United States of America; UF: University of Florida Museum of Natural History, Gainesville, Florida, United States of America; UMNH: Natural History Museum of Utah, Salt Lake City, Utah, United States of America; USNM: National Museum of Natural History, Smithsonian Institution, Washington, DC, United States of America; ZPAL MgR: Zakład Paleobiologii, Polska Akademia Nauk (Palaeobiological Institute, Polish Academy of Sciences), Warsaw, Poland.

### 2.6. Anatomical nomenclature

For most cranial and axial nomenclature in this study, we follow the terminology used by Klembara [64], Conrad *et al*. [12] and Klembara *et al*. [65]. Regarding the use of the term ‘parietal foramen’, instead of the historically employed ‘pineal foramen’, we follow the anatomical nomenclature published in Smith *et al*. [66].

## 3. Systematic palaeontology

Clade SQUAMATA Opell, 1811 [67]

Clade ANGUIMORPHA Fürbinger, 1900 [68]

Clade MONSTERSAURIA Norell & Gao, 1997 [1]

*BOLG* gen. nov.

Zoobank ID: http://zoobank.org/urn:lsid:zoobank.org:act:ACC90422-F0BD-42D3-8B84-B5E18DE1A8C2

**Diagnosis:** As for the type and only species.

**Etymology:** *Bolg*, after the fictional goblin-prince and secondary antagonist in *The Hobbit*, a novel written by one of the great literary monster creators of the 20th century, J. R. R. Tolkien. Named after a goblin due to the monstersaurian affinities of holotype specimen UMNH VP 16266 (see Diagnosis below).

*BOLG AMONDOL* sp. nov.

Zoobank ID: http://zoobank.org/urn:lsid:zoobank.org:act:55C2F63B-331E-453C-B3F5-0F02C5D0C0DF

**Diagnosis:** A large-bodied monstersaurian lizard with dermal armour that exhibits the following unique combination of characters: (i) nasal premaxillary articular surface extends at least one-third of the anteroposterior length of the nasal; (ii) presence of fused osteoderms separated by grooves on the jugal (autapomorphy); (iii) minimal retraction of the nares (similar to *Pr. nessovi* but different from *G. pulchrum*, *E. mongoliensis*, *H. horridum*); (iv) angulated dorsal maxillary border of the external naris (differing from the rounded condition in *Par. bogerti*); (v) posterior margin of the nasal tapers to form a laterally curved point (differing from the rounded condition seen in *G. pulchrum*, *C. nankangensis* and *E. mongoliensis*); (vi) posterior medial subdental margin of the dentary overhangs Meckelian groove (differing from the open condition in *L. montanaensis*; (vii) basal enamel infoldings of marginal teeth tightly spaced (differing from the wider spacing present in *M. kamitakiensis* and *Pr. nessovi*); (viii) dorsal vertebral centra with smooth ventral surfaces that lack medial pits (similar to *H. horridum*, *G. pulchrum*, *Pr. nessovi* and *Par. bogerti*, but different from *P. canadensis*); (ix) presence of autotomy septa on distal caudal vertebrae (differing from *H. horridum*).

**Etymology:** The specific epithet, *amondol*, is derived from the fictional Elvish *Sindarin* language in J. R. R. Tolkien’s *The Hobbit*, *The Lord of the Rings*, *The Silmarillion* and other works, a combination of *amon-* (Elvish, Sindarin) ‘mound’ [69], and *-dol* (Elvish, Sindarin) ‘head’ [69], named for the mound-like, fused osteoderms that cover the skull (maxilla, jugal).

**Holotype:** UMNH VP 16266, an associated fragmentary skeleton including cranial, axial, pectoral, pelvic and appendicular elements.

**Locality and horizon:** UMNH VP LOC 733, Kane County, Utah, USA. Lower half of the middle unit of the Kaiparowits Formation in the Fossil Ridge field area. Sediment at the locality consists of a fine-grained, silty sandstone associated with overbank floodplain deposition [49] exposed over the top of a low-lying ridge. Closely associated, *in situ* skeletal elements were recovered from a fossil concentration in a siltstone horizon with an area of less than 1 square metre in small (<20 cm diameter) field jackets. No other vertebrate elements were recovered within the concentration, strongly supporting the interpretation that the site contained the remains of only a single individual. Additionally, eroded skeletal fragments and partial elements were surface collected from a broad slope directly below the *in situ* site (figure 3).

cf. *Palaeosaniwa canadensis* (Gilmore, 1928) [31]

**Referred specimens:** DMNH EPV.134394, associated remains of a single individual, including a partial parietal (DMNH EPV.134394.1) and a posterior dorsal vertebra (DMNH EPV.134394.2). These elements were collected in close proximity from the same bonebed horizon containing only closely associated but disarticulated skeletons of individual vertebrate animals (e.g. two ceratopsid dinosaurs, one alligatoroid crocodylian). The absence of other squamate materials, and the association of all other skeletons in the quarry strongly suggests that these two specimens are also associated, from the same individual; DMNH EPV.132910, an isolated partial parietal.

**Locality and horizon:** DMNH Loc. 5942 (DMNH EPV.134394), Garfield County, Utah, USA. DMNH Loc.8612 (DMNH EPV.132910), Garfield County, Utah, USA. Both specimens are from the lower half of the middle unit of the Kaiparowits Formation (Late Cretaceous, late Campanian).

Monstersauria indet.

**Referred specimen:** UMNH VP 36373, a well-preserved dorsal vertebra missing the dorsal portion of the neural spine.

**Locality and horizon:** UMNH VP LOC 145, Garfield County, Utah, USA. Lower half of the middle unit of the Kaiparowits Formation (Late Cretaceous, late Campanian).

## 4. Osteological and comparative description

### 4.1. Bolg amondol

#### 4.1.1. UMNH VP 16266 (holotype)

**Figure 4.**
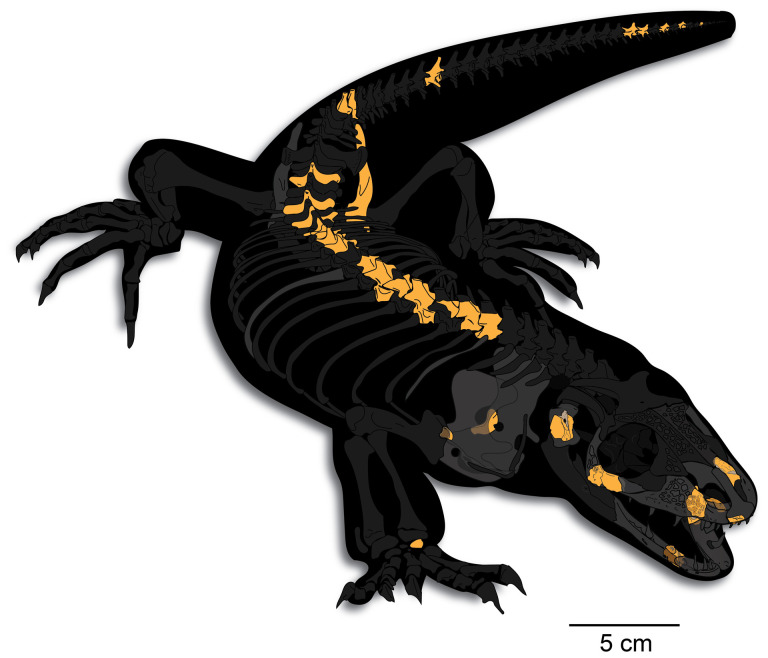
Reconstruction of UMNH VP 16266 (holotype, *Bolg amondol* gen. et sp. nov.). Gold: preserved skeletal elements. Grey: morphological hypotheses of reconstructed elements based on the morphology of preserved skeletal elements. Black: missing skeletal elements, based on publicly available rendered CT scans on morphosource.org of specimen UF:Herp:153328, *Heloderma horridum*.

UMNH VP 16266 is an associated fragmentary skeleton (figure 4) that preserves portions of the cranial, axial and appendicular regions. Identifiable elements include: portions of the premaxilla; right maxilla; a mostly complete left nasal (subsequently lost after imaging but before CT scanning); a partial right jugal; the anterior tip of the right vomer; a partial left palatine; an incomplete right quadrate; fragments of the left and right dentaries; anterior and posterior thoracic vertebrae; lumbar vertebrae; proximal, middle and distal caudal vertebrae; portions of the left and right scapulocoracoid; a mostly complete left ilium; a metapodial (possibly a radiale); as well as numerous associated vertebral, rib, and limb fragments. All of the elements of UMNH VP 16266 are proportionally correct in size for a single individual, and there is no duplication of elements. Furthermore, all material with this specimen shares the same preservation (colour and texture). This, along with the tight spatial association of the material, strongly supports our interpretation that it comprises a single associated individual. Although multitaxic aggregations of squamate material have been proposed for other Mesozoic sites (e.g. [70]), we note this interpretation is somewhat controversial because other associated skeletons of non-squamate taxa from the same deposits are well-accepted as individuals of a single taxon. Regardless, multitaxic aggregations of associated small tetrapod skeletons are not a taphonomic mode that has been documented from the Kaiparowits Formation.

### 4.2. Cranial skeleton

#### 4.2.1. Premaxilla

**Figure 5.**
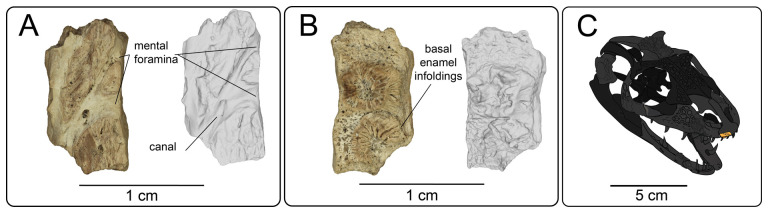
Premaxilla fragment UMNH VP 16266.4 in (A) dorsal and (B) ventral views. (C) Inferred placement of the element in a schematic reconstruction of the skull of *Bolg amondol*.

**Description:** UMNH VP 16266.4 (figure 5) is a tooth-bearing element with two partially preserved tooth bases. The dental shelf is much thinner in ventral view than the other, associated tooth bearing elements, and the teeth are smaller than the tooth bases preserved in the dentary elements. The preserved teeth in premaxilla UMNH VP 16266.4 are much larger than the palatal teeth preserved on the partial left palatine (see below). Basal enamel infoldings (plicidentine) are present in both tooth bases (figure 5B). The tooth bases are closely spaced, almost abutting one another, and are subangular in cross section. The surface opposite from the tooth-bearing margin is eroded, but a branching canal is visible on the surface (figure 5A). The preservation and size of the specimen is consistent with all other cranial materials associated with UMNH VP 16266. This specimen is tentatively referred to the premaxilla because of the short width of the tooth-bearing surface and the smaller teeth relative to the other associated marginal tooth-bearing bones for UMNH VP 16266 (e.g. maxilla, dentary).

**Comparison:** The incomplete preservation of the premaxilla of UMNH VP 16266 precludes most morphological comparisons with other taxa. However, the combination of (i) basal enamel infoldings on the teeth, and (ii) the close spacing of the teeth compare favourably to the premaxillae of numerous Cretaceous anguimorph taxa, including those of *Parasaniwa wyomingensis* [7], *Paraderma bogerti* [3], *Gobiderma pulchrum* [12], *Estesia mongoliensis* [5,6], as well as extant anguimorphs *Heloderma suspectum* [12], *Varanus* [5] and *Lanthanotus borneensis* [5].

#### 4.2.2. Maxilla

**Figure 6.**
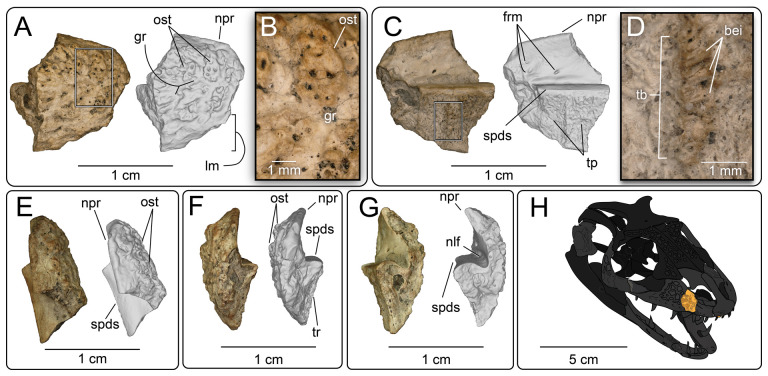
Photographs (left side of panels) and rendered CT scans (right side of panels) of the right maxilla UMNH VP 16266.3 (*Bolg amondol*). (A) lateral view; (B) magnified view of osteoderms separated by grooves on the lateral surface of the nasal process; (C) medial view; (D) magnified view of the tooth-bearing surface, showing traces of basal enamel infoldings at the incomplete tooth base; (E) dorsal view; (F) anterior view; (G) posterior view. (H) Inferred placement of the element in a schematic reconstruction of the skull of *Bolg amondol*. Anatomical abbreviations: bei, basal enamel infoldings; frm, foramina; gr, grooves; lm, labial margin; nlf, nasolacrimal fossa; npr, nasal process; ost, osteoderms; spds, supradental shelf; tb, tooth base; tp, tooth position; tr, tooth row.

**Figure 7.**
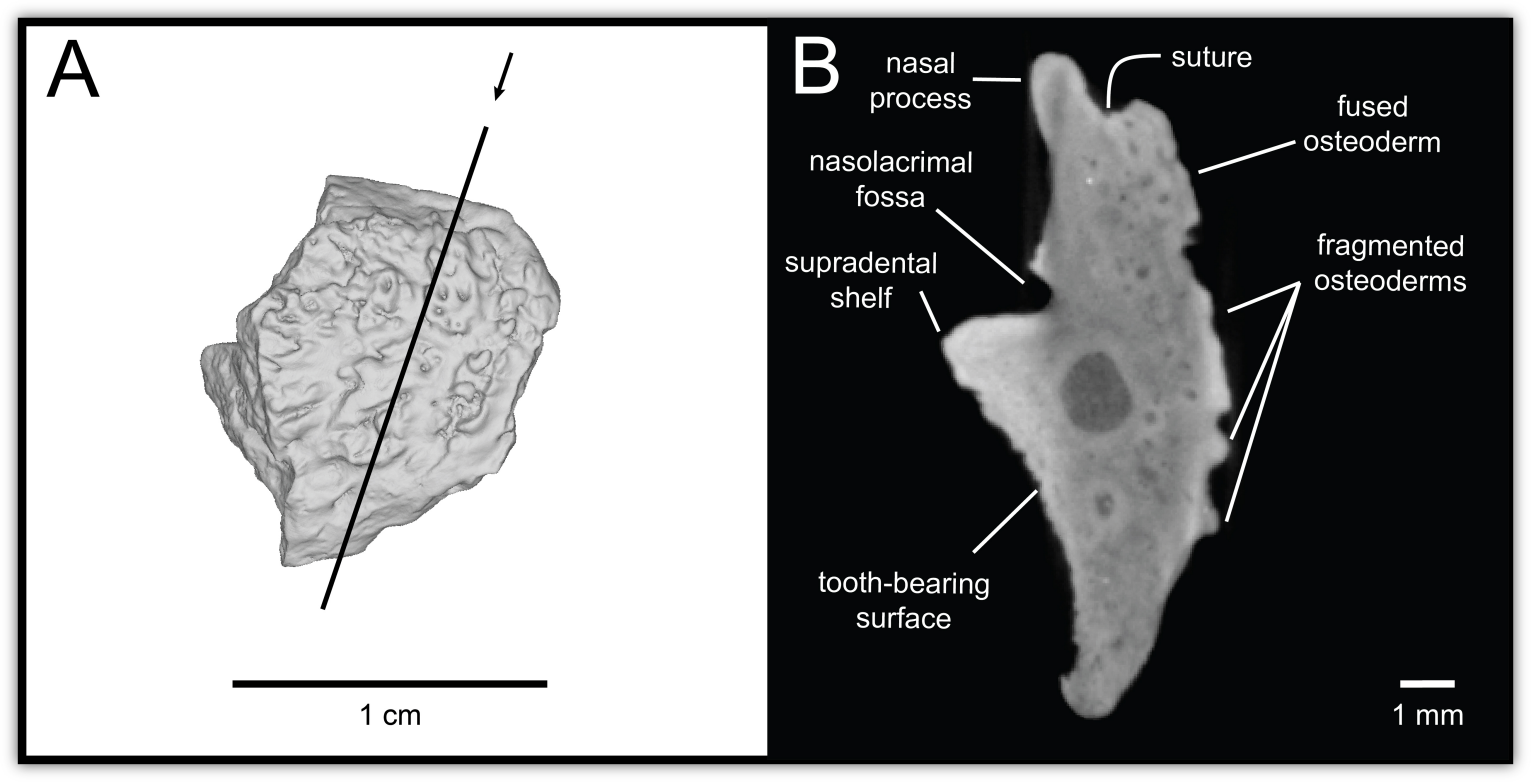
CT data illustrating osteoderm attachment to the lateral surface of maxilla UMNH VP 16266.3 (*Bolg amondol*). (A) Rendered CT scan of UMNH VP 16266.3 in lateral view. Black line indicates position and angle of CT slice displayed in (B). (B) Oblique transverse CT slice illustrating osteoderm attachment to the lateral surface of the maxilla.

**Description:** UMNH VP 16266.3 (figure 6) is the anterior portion of the right maxilla, preserving portions of two tooth positions and the anterior margin of the nasal process. Pitted/vermiculate osteoderms are fused to the external surface of the nasal process (figures 6 and 7). Grooves are present between osteoderms, dividing them into polygonal shapes (figure 6A,B). The osteoderms extend inferiorly to the edge of a smooth labial margin, a fragment of which is preserved on this specimen. The rest of the labial margin is eroded away, and no mental foramina are preserved. The angle of the apical portion of the nasal process is gently inclined from the supradental shelf (figure 6C), the angle steepening in the anterior-most preserved end to form the posterior/ventral border of the naris. We interpret this feature to indicate a naris that is not retracted, as observed in the middle Cretaceous (Cenomanian) monstersaur *Primaderma nessovi* [10]. On the medial surface of the facial process, three small, posterior directed foramina are present (figure 6C). Additionally, a groove is present at the base of the facial process in medial view (figure 6C). This is interpreted as the anterior portion of the nasolacrimal fossa, as observed in *Heloderma suspectum* [21]. Tooth attachment is modified pleurodont, and fragments of basal enamel infoldings are present on the anterior tooth position (figure 6D). The supradental shelf curves medially relative to the lateral margin of the nasal process (figure 6E), similar to other monstersaur taxa such as *Estesia mongoliensis* [5,6], *Gobiderma pulchrum* [2,12], *Paraderma bogerti* [3,22] and *Heloderma suspectum* [12].

**Figure 8.**
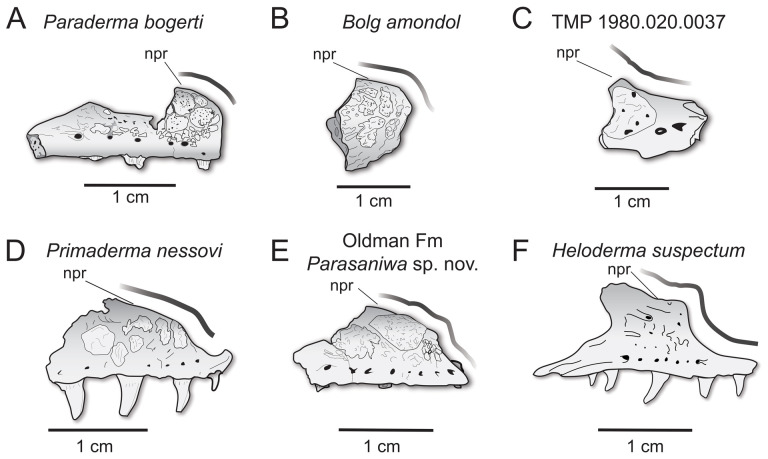
Comparisons of anterior nasal processes of monstersaurian maxillae in lateral view. Thick grey line follows the anterior margin of the nasal process of the maxilla. (A) Mirror-imaged line drawing of *Paraderma bogerti* holotype left maxilla UCMP 54261 from the Maastrichtian Lance Formation (Fm), Wyoming, modified from Pregill *et al*. [22]. (B) Line drawing of UMNH VP 166266.3 (*Bolg amondol*) from the upper Campanian Kaiparowits Fm, Utah. (C) Line drawing of right maxilla TMP 1980.020.0037, referred to *Palaeosaniwa canadensis* from the upper Campanian Oldman Fm, Alberta, modified from Gao & Fox [7]. (D) Line drawing of OMNH 26742 holotype right maxilla of *Primaderma nessovi* from the Albian–Cenomanian Cedar Mountain Fm, modified from Nydam [10]. (E) Line drawing of UALVP 33346, a right maxilla referred to a new species of Parasaniwa from the upper Campanian Oldman Fm, modified from Gao & Fox [7]. (F) Line drawing of the right maxilla of *Heloderma suspectum*. Anatomical abbreviations: npr, nasal process.

**Figure 9.**
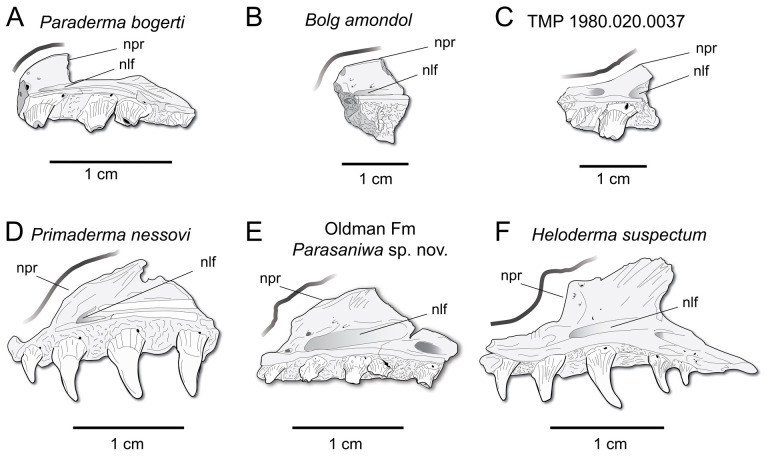
Comparisons of anterior nasal processes and nasolacrimal fossae of monstersaurian maxillae in medial view. Thick grey line follows the anterior margin of the nasal process of the maxilla. (A) Mirror-imaged line drawing of *Paraderma bogerti* holotype left maxilla UCMP 54261 from the Maastrichtian Lance Formation (Fm), Wyoming, modified from Pregill *et al*. [22]. (B) Line drawing of UMNH VP 166266.3 (*Bolg amondol*) from the upper Campanian Kaiparowits Fm, Utah. (C) Line drawing of right maxilla TMP1980.020.0037, referred to *Palaeosaniwa canadensis* from the upper Campanian Oldman Fm, Alberta, modified from Gao & Fox [7]. (D) Line drawing of OMNH 26742 holotype right maxilla of *Primaderma nessovi* from the Albian–Cenomanian Cedar Mountain Fm, modified from Nydam [10]. (E) Line drawing of UALVP 33346, a right maxilla referred to a new species of *Parasaniwa* from the upper Campanian Oldman Fm, modified from Gao & Fox [7]. (F) Line drawing of the right maxilla of *Heloderma suspectum*. Anatomical abbreviations: npr, nasal process; nlf, nasolacrimal fossa.

**Comparison:** The maxilla of UMNH VP 16266 (UMNH VP 16266.3) preserves several features that merit comparison to contemporaneous and extant anguimorph taxa. The presence of pitted, polygonal osteoderms separated by grooves on the external surface of the maxilla compares favourably with Oldman Fm *Parasaniwa* sp. nov. (UALVP 33346 [7]; figure 8E), *Primaderma nessovi* (figure 8D) [10], *Paraderma bogerti* (figure 8A) [3,22] and *Lowesaurus matthewi* [17,^22^]. In terms of osteoderm morphology and patterning, UMNH VP 16266.3 most resembles the holotype maxilla of the Maastrichtian North American monstersaur, *Paraderma bogerti* (UCMP 54261 [3,22]). Similar to *Paraderma*, the nasal process of the maxilla curves strongly medially in anterior and posterior views (figure 6F,G). However, a major difference between UMNH VP 16266 and UCMP 54261 is in the relative height of the anterior margin of the nasal process in lateral view (figure 8A,B) and in medial view (figure 9A,B). The nasal process of UMNH VP 16266.3 is shorter than observed in UCMP 54261 (figures 8A and 9A), suggesting that the nares for *Bolg amondol* were dorsoventrally shallower than those observed in *Paraderma bogerti*, in addition to being unretracted. The dorsal margin of the nasal process of UCMP 54261 is also much more rounded than UMNH VP 16266.3, which is more angulated in lateral (figure 8B) and medial (figure 9B) views. One additional difference between UMNH VP 16266.3 and UCMP 54261 is the slope and extent of the dental attachment surface relative to the supradental shelf. The maxillary tooth attachment surface of UMNH VP 16266 is much more medially directed than that observed in UCMP 54261 [22], in which it is more ventrally directed throughout the tooth row.

In medial view (figure 9), additional differences are evident in the curvature of the dorsal margin of the anterior nasal process in UCMP 54261 (*Paraderma bogerti*) and UMNH VP 16266.3 (*Bolg amondol*). The proportions and positions of the nasolacrimal fossae on UCMP 54261 and UMNH VP 16266.3 are similar. What appears to be the anterior tip of the nasolacrimal fossa on TMP 1980.020.0037 (figure 9C) is dorsoventrally expanded relative to those observed in UMNH VP 16266 and UCMP 54261.

The evident absence of significant narial retraction in UMNH VP 16266 is similar to the interpretation of the holotype maxilla of the Cenomanian monstersaur *Primaderma nessovi* [10], although the height of the anterior nasal process of *Primaderma nessovi* is relatively greater (figures 8D and 9D) than UMNH VP 16266. The absence of retracted nares is also interpreted in specimen TMP 1980.020.0037 (figures 8C and 9C) [7], a fragmentary anterior maxilla from the Oldman Formation of Alberta, referred to *Palaeosaniwa canadensis* based on size and stratigraphic overlap with the holotype vertebra [7]. Although fragmentary, the ascending nasal process of TMP 1980.020.0037 is even shallower than that observed on UMNH VP 16266 (figures 8C and 9C). Additionally, while modest dermal sculpturing is present on TMP 1980.020.0037, osteoderms are absent. However, it is important to acknowledge that, in squamates, the presence of osteoderms may be intraspecifically variable [71]. In sum, UMNH VP 16266.3 possesses a unique combination of traits that differentiates this specimen from other monstersaurs: grooved osteoderms fused to the maxilla; relatively short, angulated rather than smooth anterior nasal process; unretracted nares; and a medially facing tooth attachment surface.

#### 4.2.3. Nasal

**Figure 10.**
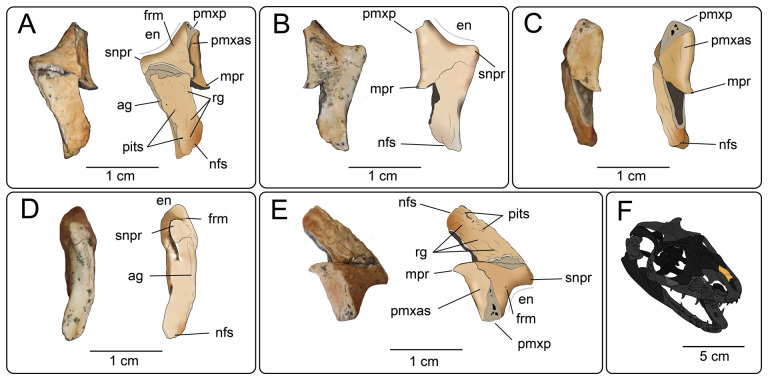
Photographs (left side of panels) and line drawings (right side of panels) of the left nasal UMNHVP 16266.1 (*Bolg amondol*) in (A) dorsal, (B) ventral, (C) medial, (D) lateral and (E) anteromedial views. (F) Inferred placement of the element in a schematic reconstruction of the skull of *Bolg amondol*. Anatomical abbreviations: ag, articulation groove; en, external naris; frm, foramen; mpr, medial process; nfs, nasofrontalsuture; pmxas, premaxillary articular surface; pmxp, premaxillary process; rg, rugosities; snpr, supranasal process.

**Description:** UMNH VP 16266.1 (figure 10) is a mostly complete left nasal, with a portion of the premaxillary process eroded away and a portion of the mediolateral margin missing (figure 10A). Overall, the nasal is gently curved dorsoventrally (figure 10C,D) and gradually tapers posteriorly in dorsal and ventral views (figure 10A,B). The dorsal surface exhibits strong rugosities with two pits/foramina along the posterolateral margin (figure 10A,E). The preserved medial articular surface of the nasal is broad, indicating extensive contact ventrolateral to the presumably broad nasal process of the premaxilla (figure 10A). Based on how squamate nasal processes tend to contact the nasal (e.g. Gauthier et al. [25] Char. 24−25), we hypothesize that due to the wide and shallowly dipping premaxillary articular surface on the nasal bone of UMNH VP 16266.1, the nasal process of the premaxilla would also have been broad. In dorsal and ventral views, a preserved medial process is apparent (figure 10A,B), potentially signifying the termination of the contact with the nasal process of the premaxilla. The premaxilla–nasal contact therefore extends posteriorly just beyond the posterior margin of the external naris (figure 10A). A descending lamina emanating from the ventral surface of the nasal is absent. In dorsal view, the supranasal process forms a concave anterior border such that it forms the posterior border of the external naris (figure 10A,E). A small, anteriorly directed foramen is present on the lateral surface of the premaxillary process, connecting with the external naris (figure 10A,E). The supranasal process tapers ventrolaterally to a point where it presumably meets the ascending nasal process of the maxilla to form the posterior border of the external naris. A shallow groove running anteroposteriorly along the lateral margin of the nasal indicates continuous contact with the nasal process of the maxilla and/or the prefrontal, as observed in other monstersaur taxa (e.g. *Gobiderma pulchrum* [12], *Heloderma suspectum* [12]; figure 10D). In dorsal view, the anterior mediolateral width of the nasal is greater than the posterior mediolateral width, with the posterior lamina of the nasal presumably in contact with the frontal(s) (figure 10A). The frontal sutures are somewhat worn but indicate weak interdigitation with the frontal (figure 10A–D). Additionally, the posterior margin of the nasal appears to have a small, medial facet where an anterior prong of the frontal invaded, producing a W-shaped frontonasal suture in dorsal view when complete (figure 10A).

**Figure 11.**
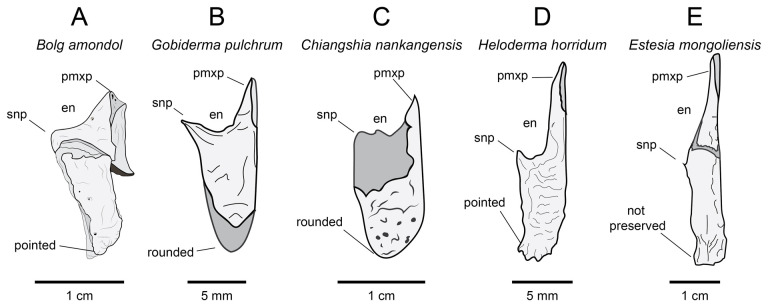
Comparisons of left nasals of *Bolg amondol* to select anguimorph and monstersaur taxa in dorsal view. (A) Line drawing of UMNH VP 16266.1 (holotype, *Bolg amondol*). (B) Line drawing of the left nasal of ZPAL MgR III/64 (holotype, *Gobiderma pulchrum*, modified from [12]. (C) Mirrored line drawing of the right nasal of NHMG 009318 (holotype, *Chiangshia nankangensis*, modified from [13]). (D) Line drawing of the left nasal of UF 153328 (*Heloderma horridum*). (E) Line drawing of the left nasal of IGM 3/14 (holotype, *Estesia mongoliensis*, modified from [5]). Anatomical abbreviations: en, external naris; pmxp, premaxillary process; snpr, supranasal process.

**Comparison:** Compared with closely related anguimorph taxa (figure 11), UMNH VP 16266.1 does not bear explicit morphology that indicates close affinities to one taxon over another. However, a suite of features are similar to correlative regions on the nasals of other taxa. For instance, UMNH VP 16266.1 exhibits an extensive medial articular surface for the premaxilla, also present in *Pseudopus apodus* [65] and *Gobiderma pulchrum* [12] (figure 11B). However, the premaxillary articular surface extends further posteriorly than in any of the taxa used here for comparison (figure 11), a potentially autapomorphic feature of this taxon. The anterior narial margin of UMNH VP 16266.1 forms an obtuse angle that stretches from the supranasal process to the premaxillary process, similar to *Pseudopus apodus* [65], *Gobiderma pulchrum* [12], *Chiangshia nankangensis* [13] and *Heloderma suspectum* [12], but differing from *Shinisaurus crocodilurus* [72] and *Estesia mongoliensis* [5,6]. Additionally, the posterior margin of UMNH VP 16266.1 ‘hooks’ laterally, similar to the anguid *Pseudopus apodus* [65] and *Heloderma suspectum* [12], but different from *Shinisaurus crocodilurus* [72], *Gobiderma pulchrum* [12], *Chiangshia nankangensis* [13] and *Estesia mongoliensis* [5,6]. The posterior tapering of the nasal of UMNH VP 16266.1 is similar to *Shinisaurus crocodilurus* [72], *Pseudopus apodus* [65] and *Gobiderma pulchrum* [12]. The dermal rugosities on the dorsal surface suggest potential attachment for osteoderms, as observed in *Gobiderma pulchrum* [12], *Chiangshia nankangensis* [13] and *Heloderma suspectum* [12,20], but different from *Estesia mongoliensis* [5,6]. This suite of features found on UMNH VP 16266.1 are a unique combination not found in other monstersaur/anguimorph taxa.

#### 4.2.4. Jugal

**Figure 12.**
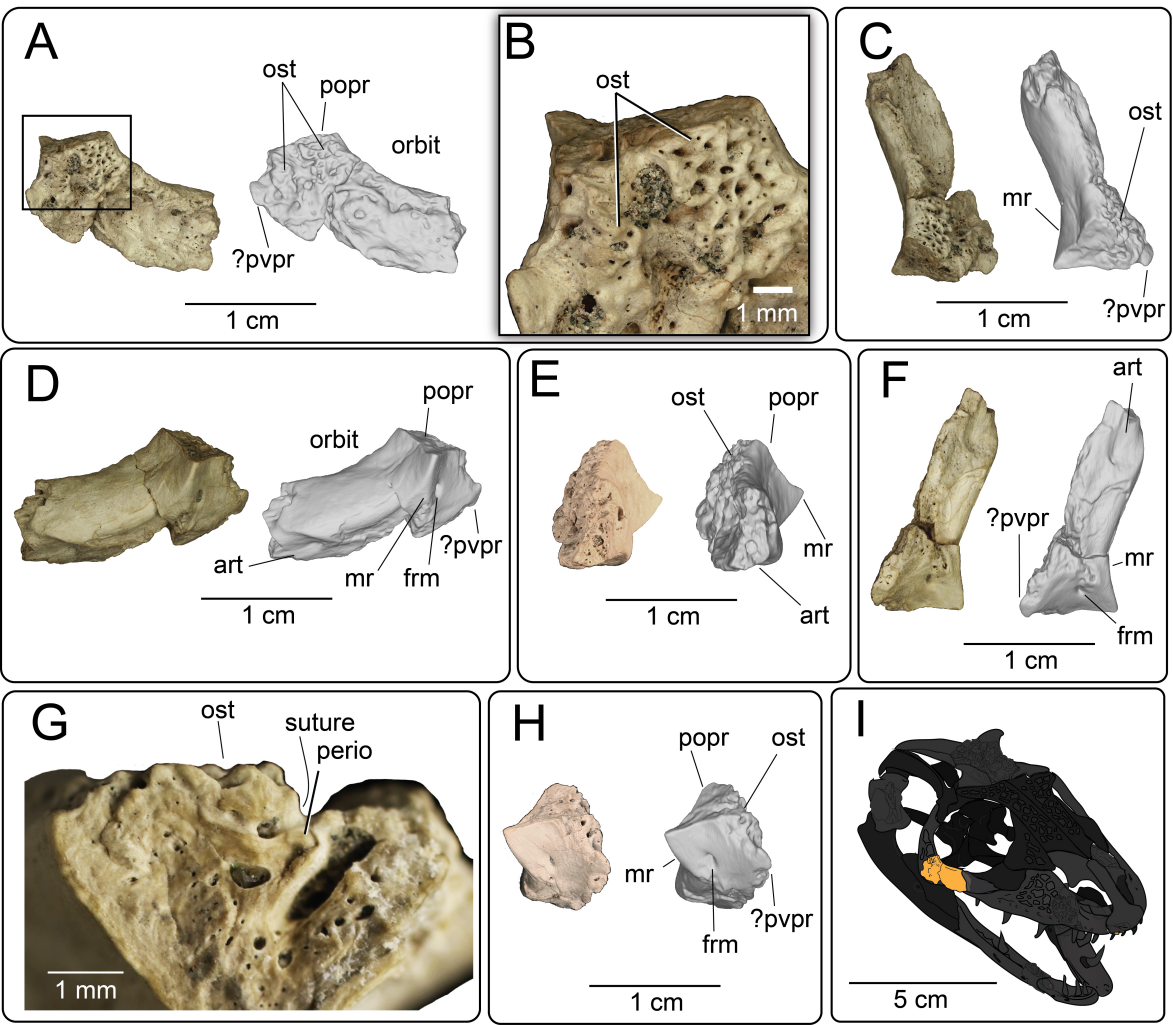
Photographs (left side of panels) and rendered CT scans (right side of panels) of the right jugal UMNH VP 16266.2 (*Bolg amondol*). (A) Lateral view. (B) Magnified view of osteoderms on postorbital process. (C) Dorsal view. (D) Medial view. (E) Anterior view. (F) Ventral view. (G) Magnified dorsomedial view, illustrating attachment of osteoderms to the postorbital process. (H) Posterior view. (I) Inferred placement of the element in a schematic reconstruction of the skull of *Bolg amondol*. Anatomical abbreviations: frm, foramen; mr, medial ridge; ost, osteoderms; perio, periosteum; popr, basal portion of postorbital process; pvpr, posteroventral process.

**Figure 13.**
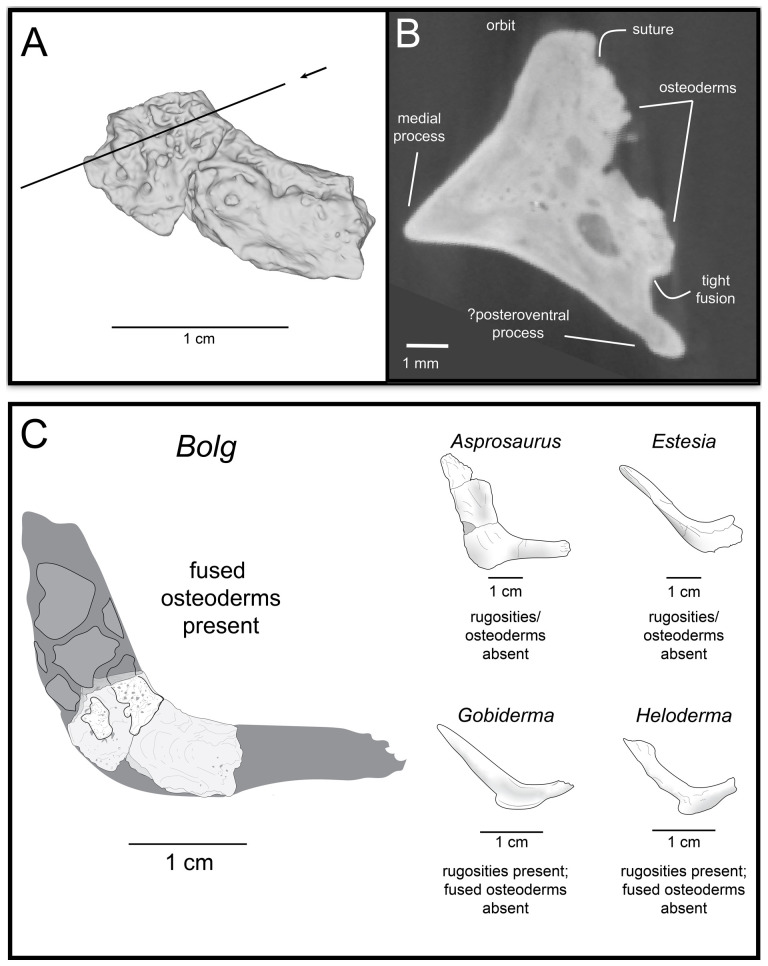
(A) Rendered CT scan of UMNH VP 16266.2 (right jugal, *Bolg amondol*) in lateral view. Black line indicates position and angle of CT slice displayed in (B). (B) Oblique transverse CT slice illustrating osteoderm attachment to the lateral surface of the postorbital process of the jugal. (C) Comparisons of dermal sculpturing on the jugal postorbital process in lateral view among select monstersaur taxa (see §5.2 for phylogenetic placement of *Asprosaurus*). Line drawings: *Bolg amondol* UMNH VP 16266.2; *Asprosaurus bibongriensis* KDRC-BB4, modified from [14]; *Estesia mongoliensis*, mirrored, IGM 3/14, modified from [5]; *Gobiderma pulchrum*, IGM 3/55, modified from [12]; *Heloderma horridum*, UF 153328.

**Figure 14.**
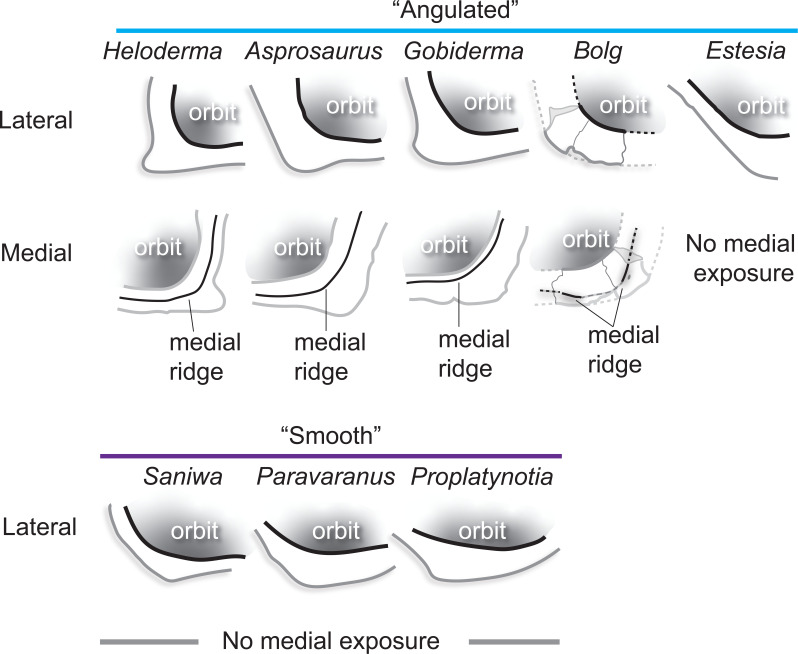
Comparisons of the shape of the orbital margin of the jugal in lateral view among monstersaur and varanoid taxa. Line drawings not to scale. ‘Angular’ jugals modified from same references as in figure 13. *Saniwa ensidens*: FMNH PR 2378 [74]; *Paravaranus angustifrons*: ZPAL MgR I/67 [2]; *Proplatynotia longirostrata*: ZPAL MgR I/68 [2].

**Description:** UMNH VP 16266.2 (figure 12) is the ventromedial portion of a right jugal, preserving a segment of the suborbital margin and the base of the postorbital process. The external surface of the postorbital process preserves pitted rugosities that, in cross-sectional view, appear to represent fused osteoderms (figure 12G). CT scan images of UMNH VP 16266.2 reveal thin suture lines between the bases of the raised osteoderms and the periosteum/cortical bone (figure 13). The medial ridge of the jugal is pronounced and triangular in cross-section at the base of the postorbital process (figure 12A–F,H). In medial, dorsal, and ventral views, the preserved portions of the medial crest form an angulated line (figure 12C*–*F,H), suggesting that the overall shape of the jugal is angulated (Conrad [73] Char. 47 = 0; see figure 14 for examples). The anterior portion of the jugal is broken; the nature of contact with the maxilla is absent. However, a portion of an anteromedial articular surface can be observed in medial, anterior and ventral views (figure 12D*–*F). This could be an articular surface for the palatine, ectopterygoid or maxilla, as in *Heloderma horridum* (specimen UF:Herp:153328) and *Pseudopus apodus* [65].

**Comparison:** The angulated orbital margin of the jugal UMNH VP 16266.2 is present in monstersaur taxa *Gobiderma pulchrum* [12], *Heloderma* [12], *Asprosaurus bibongriensis* [14] and *Estesia mongoliensis* [5,6], but differs from fossil varanoid taxa *Saniwa ensidens* [74], *Proplatynotia longirostra* [2] and *Cherminotus angustifrons* [2] (figure 14). Additionally, the ornamentation on the postorbital process of UMNH VP 16266.2 is so extensive that it is most likely to represent fused osteoderms. Although osteoderms are present on the jugals of monstersaur taxa such as *Gobiderma pulchrum* [12] and *Heloderma* (e.g. LACM 163852), they are not fused to the bone as extensively as observed in UMNH VP 16266.2. Other hypothesized fossil monstersaurs that preserve jugals, such as *Asprosaurus bibongriensis* [14] and *Estesia mongoliensis* [5,6], do not preserve osteoderms and have thus been inferred to lack them. Although the nature of dermal ossification morphology and attachment has been shown to be extremely variable, even across individuals of a given species [20], the tightly fused ornamentation/osteoderms present on the postorbital process of the UMNH VP 16266.2 has not been reported for any other monstersaurian.

#### 4.2.5. Vomer

**Figure 15.**
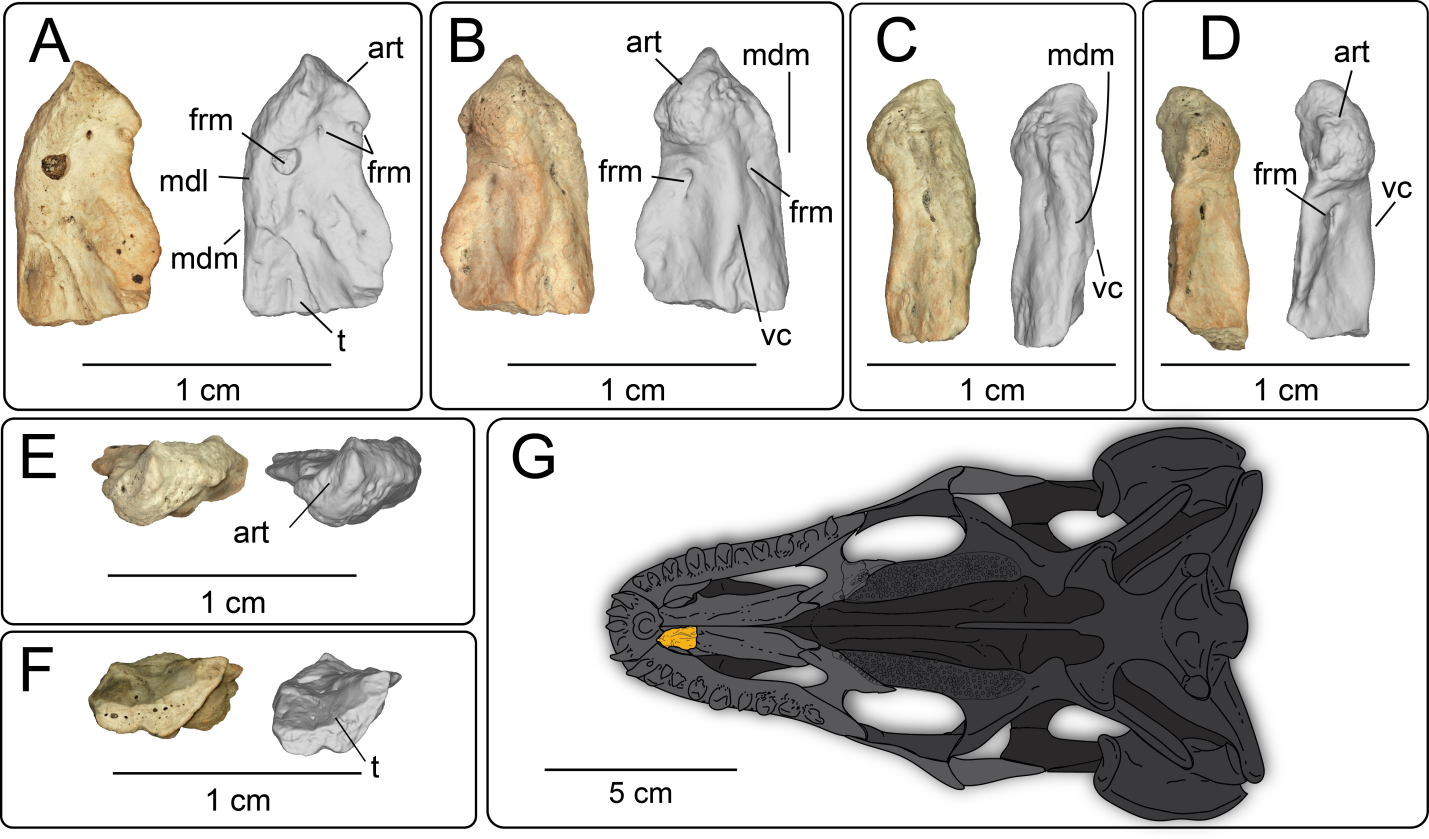
Photographs (left side of panels) and rendered CT scans (right side of panels) of the right vomer UMNH VP 16266.25 (*Bolg amondol*) in (A) dorsal, (B) ventral, (C) medial, (D) lateral, (E) anterior and (F) posterior views. (G) Inferred placement of the element in a schematic reconstruction of the skull of *Bolg amondol*, modified from the ventral view of *Gobiderma pulchrum* [12]. Anatomical abbreviations: art, articular surface; frm, foramen; mdl, medial lamina; mdm, medial margin; t, trough; vc, ventral crest.

**Description:** UMNH VP 16266.25 (figure 15) is the anterior tip of the right vomer. This preserved segment of the vomer is mediolaterally narrow, and the articular surface is also narrow and directed anterolaterally at roughly a 45° angle from the main body of the vomer (figure 15A,B). On the dorsal surface, posteromedial to the articular surface, is a small, posteriorly opening foramen. Just lateral to the medial lamina on the dorsal surface, a larger circular, dorsally opening foramen excavates a thick ridge along the medial margin, though this may be a post-depositional diagenetic or erosional feature. Posterolateral to this foramen is a ventrally convex trough that widens posteriorly. In ventral view (figure 15B), the anterolateral margin is lightly curved, forming a portion of the medial margin of the vomeronasal fenestra. The ventral crest is prominent (figure 15B,D), and connects to the margin of the articular surface. On both the medial and lateral margins of the ventral crest are foramina that are roughly equal in size (figure 15B*–*D).

**Figure 16.**
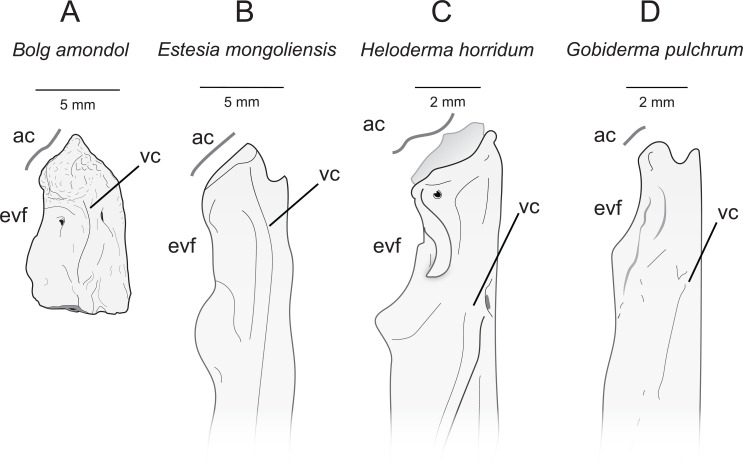
Comparisons of anterior right vomers of *Bolg amondol* (A) and selected monstersaur taxa. (B) Right vomer of IGM 3/14 (holotype, *Estesia mongoliensis*, modified from [5]). (C) Right vomer of UF 153328 (*Heloderma horridum*). (D) Right vomer of *Gobiderma pulchrum*, modified from [12]. Anatomical abbreviations: ac, articular crest; evf, external vomeronasal fenestra; frm, foramen; vc, ventral crest.

**Comparison:** The ventral surface UMNH VP 16266.25 compares favourably with preserved vomers of other monstersaur and anguimorph taxa (figure 16). The angle of the anterior articular facet of UMNH VP 16266.25 is similar to the 45° angle from the long axis of the vomer seen in *Estesia mongoliensis* and *Gobiderma pulchrum*, whereas the vomer of *Heloderma horridum* has an anterior articular facet that is nearly perpendicular to the long axis of the vomer. The margin along the external vomeronasal fenestra in UMNH VP 16266.25 is shallowly concave in ventral view (as opposed to relatively deeply concave in *Heloderma horridum*), and the posterior edge is marked by a gently sloping process, similar to *Estesia mongoliensis* and *Gobiderma pulchrum* (figure 16B,D). The medial margin of the external vomeronasal fenestra in *Estesia mongoliensis* is anteroposteriorly expanded compared with UMNH VP 16266.25 and *Gobiderma pulchrum*. The ventral crest of UMNH VP 16266.25 terminates anteriorly on the anterior articular crest, a trait shared with *Estesia mongoliensis*. Unique to UMNH VP 16266.25 among compared taxa is the presence of anterior foramina on the medial and lateral edges of the ventral crest.

#### 4.2.6. Palatine

**Figure 17.**
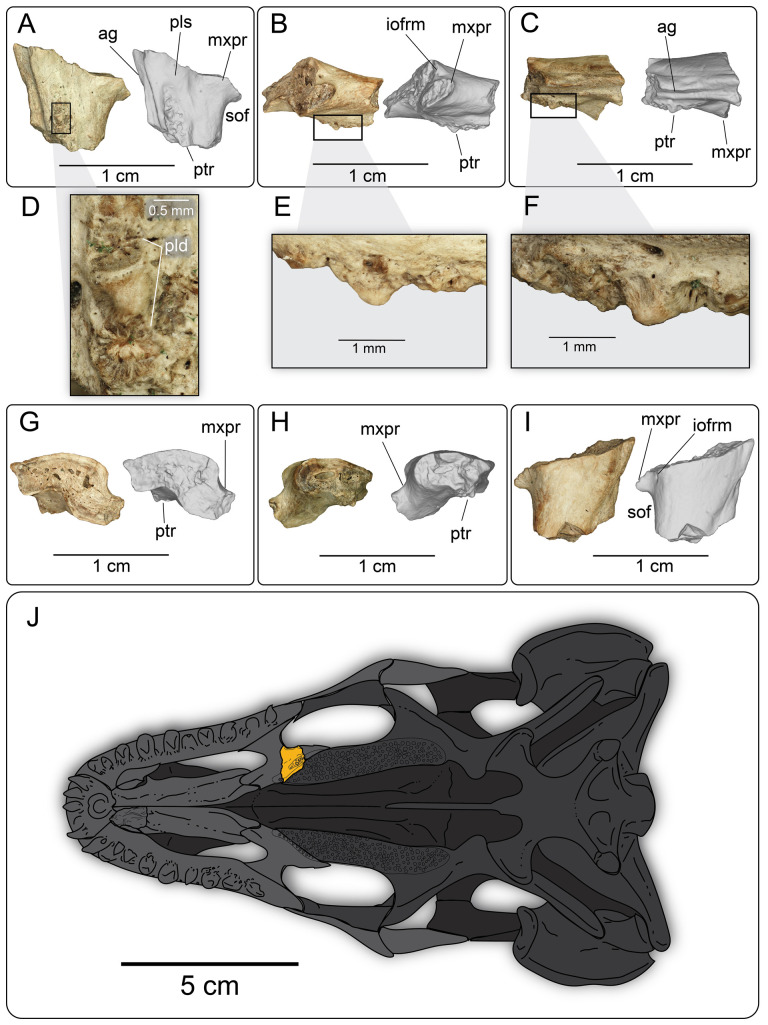
Photographs (left side of panels) and rendered CT scans (right side of panels) of the left palatine UMNH VP 16266.26 (*Bolg amondol*) in (A) ventral, (B) lateral and (C) medial views. (D) Close-up image of palatine tooth row in ventral view. (E) Close-up image of the palatine tooth row in lateral view. (F) Close-up image of the palatine tooth row in medial view. (G) Anterior, (H) posterior and (I) dorsal views. (J) Inferred placement of the element in a schematic reconstruction of the skull of *B. amondol*, modified from the ventral view of *Gobiderma pulchrum* [12]. Anatomical abbreviations: ag, articulation groove; iofrm, infraorbital foramen; mxpr, maxillary process; pls, palatine sulcus; ptr, palatine tooth row; sof, suborbital fenestra.

**Description:** UMNH VP 16266.26 (figure 17) is a portion of the left palatine, preserving the anterior portion of a palatal tooth row, the anterior margin of the suborbital fenestra, and the posterior portion of the palatine sulcus. In ventral view (figure 17A), the medial portion of the palatine preserves a prominent groove for articulation with the pterygoid. The palatal teeth (figure 17D*–*F) are present in a single row, with one preserved conical, recurved tooth crown. The palatal teeth preserve basal enamel infoldings, as in the preserved marginal tooth bases. In dorsal view (figure 17I), the posterior portion of the infraorbital foramen is visible, with a small, laterally directed fossa (figure 17B).

**Figure 18.**
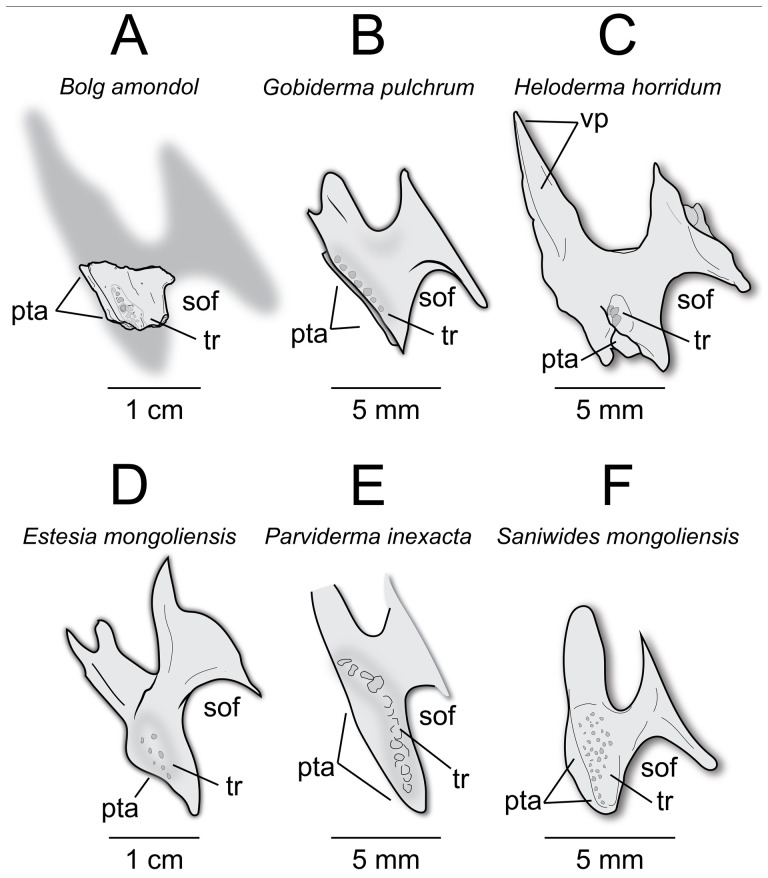
Comparisons of the palatine in select monstersaur taxa (A–D) and close outgroups (E,F) in ventral view. (A) Line drawing of the left palatine of UMNH VP 16266 (holotype, *Bolg amondol*). (B) Line drawing of the left palatine of *Gobiderma pulchrum*, modified from [12]. (C) Left palatine of UF 153328 (*Heloderma horridum*). (D) Left palatine of IGM 3/196 (*Estesia mongoliensis*, modified from [6]). (E) Left palatine of ZPAL MgR-I/43 (holotype, *Parviderma inexacta*, modified from [2]). (F) Left palatine ZPAL MgR-I/72 (holotype, *Saniwides mongoliensis*, modified from [2]). Anatomical abbreviations: pta, pterygoid articulation; sof, suborbital fenestra; tr, palatine tooth row; vp, vomerine process.

**Comparison:** The presence of recurved palatal teeth with basal enamel infoldings forming a row parallel and adjacent to the pterygoid articulation is similar to *Gobiderma pulchrum* (figure 18B). The palatine of *Heloderma horridum* (UF:Herp:153328, figure 18C) preserves a pair of recurved palatal teeth that are arranged almost perpendicular to the pterygoid articulation, whereas the palatine of *Estesia* (figure 18D) possesses a palatal tooth row that is arranged mostly anterior to the pterygoid articulation. *Parviderma* (figure 18E) exhibits a tooth row that is subparallel to the pterygoid articulation, but distinctively curves laterally from the pterygoid articulation, forming a gentle apex in palatal view. The preserved portion of the maxilla/jugal process on UMNH VP 16266.26 protrudes roughly perpendicular to the long axis of the element, similar to *Gobiderma*, *Heloderma*, *Estesia*, *Parviderma* and *Saniwides* (figure 18).

#### 4.2.7. Quadrate

**Figure 19.**
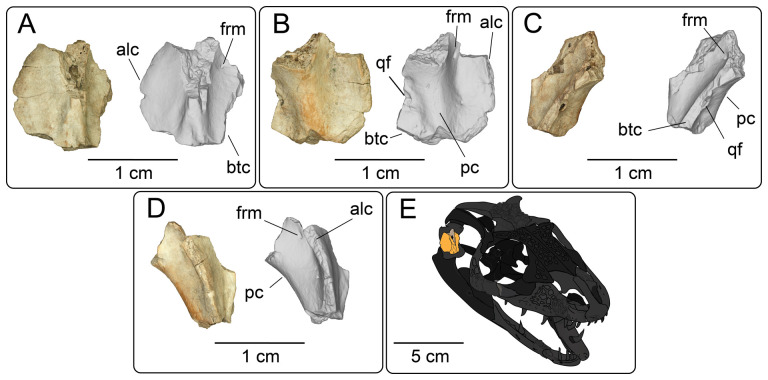
Photographs (left side of panels) and rendered CT scans (right side of panels) of the right quadrate UMNH VP 16266.7 (*Bolg amondol*) in (A) anterior, (B) posterior, (C) medial and (D) lateral views. (E) Inferred placement of the element in a schematic reconstruction of the skull of *Bolg amondol*. Anatomical abbreviations: alc, anterolateral crest; btc, base of tympanic crest; frm, foramen; pc, posterior crest; qf, quadrate foramen.

**Description:** UMNH VP 16266.7 (figure 19) is the partial right quadrate, missing the cephalic condyle and dorsal tuber as well as the articular condyle. As such, the nature of articulation of UMNH VP 16266.7 to the rest of the cranium and the mandible is impossible to determine. The basal portion of the anterolateral crest is preserved, which flares laterally in anterior and posterior view (figure 19A,B). The basal portion of the tympanic crest is preserved, with a large foramen that opens medially. This quadrate foramen is interpreted as the medial terminus of the quadrate canal [65] (figure 19B). The posterior crest is prominent in lateral view (figure 19D), and forms a gently sloping ridge at the midline (figure 19B,D).

**Figure 20.**
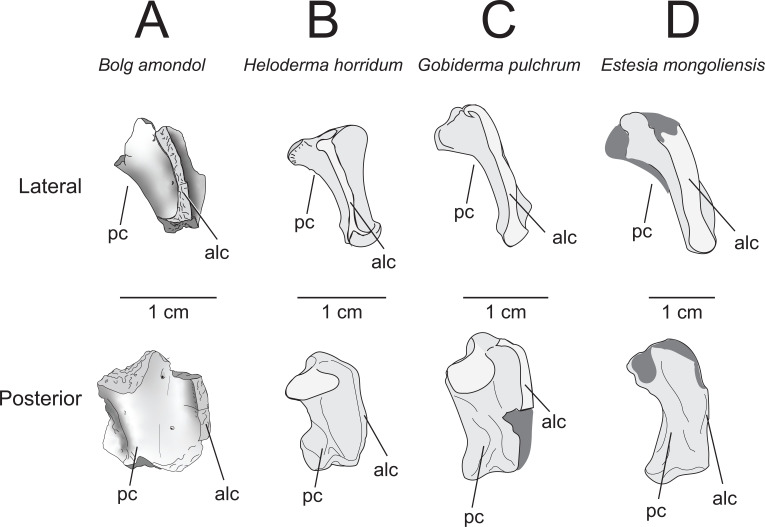
Comparisons of quadrates of select monstersaur taxa, in lateral view (top row) and posterior view (bottom row). (A) UMNH VP 16266.7 (holotype, *Bolg amondol*). (B) LACM 163582 (*Heloderma horridum*). (C) IGM 3/905 (referred specimen, *Gobiderma pulchrum*, modified from [12]). (D) IGM 3/14 (holotype, *Estesia mongoliensis*, modified from [5,12]). Anatomical abbreviations: alc, anterolateral crest; pc, posterior crest.

**Comparison:** Though much of the morphology is missing from UMNH VP 16266.7, the extensive, flared anterolateral crest is similar to that of other monstersaur taxa, such as *Heloderma*, *Gobiderma* and *Estesia* (figure 20), and is different from the less extensive flare of varanids (e.g. *Varanus komodoensis* [75]) and anguids (e.g. *Pseudopus apodus* [65]). Although there is an obvious quadrate foramen on UMNH VP 16266.7, the incompleteness of the tympanic crest makes it difficult to determine how the foramen passes through the crest, as can be observed in *Gobiderma pulchrum* (anteromedial passage [12]). A prominent quadrate foramen was not described by Norell *et al*. [5] on the quadrate of the holotype specimen of *Estesia mongoliensis*, nor is a prominent medial quadrate foramen obvious in observed specimens of *Heloderma* (specimen UF:Herp:153328, LACM 163852).

#### 4.2.8. Dentary

**Figure 21.**
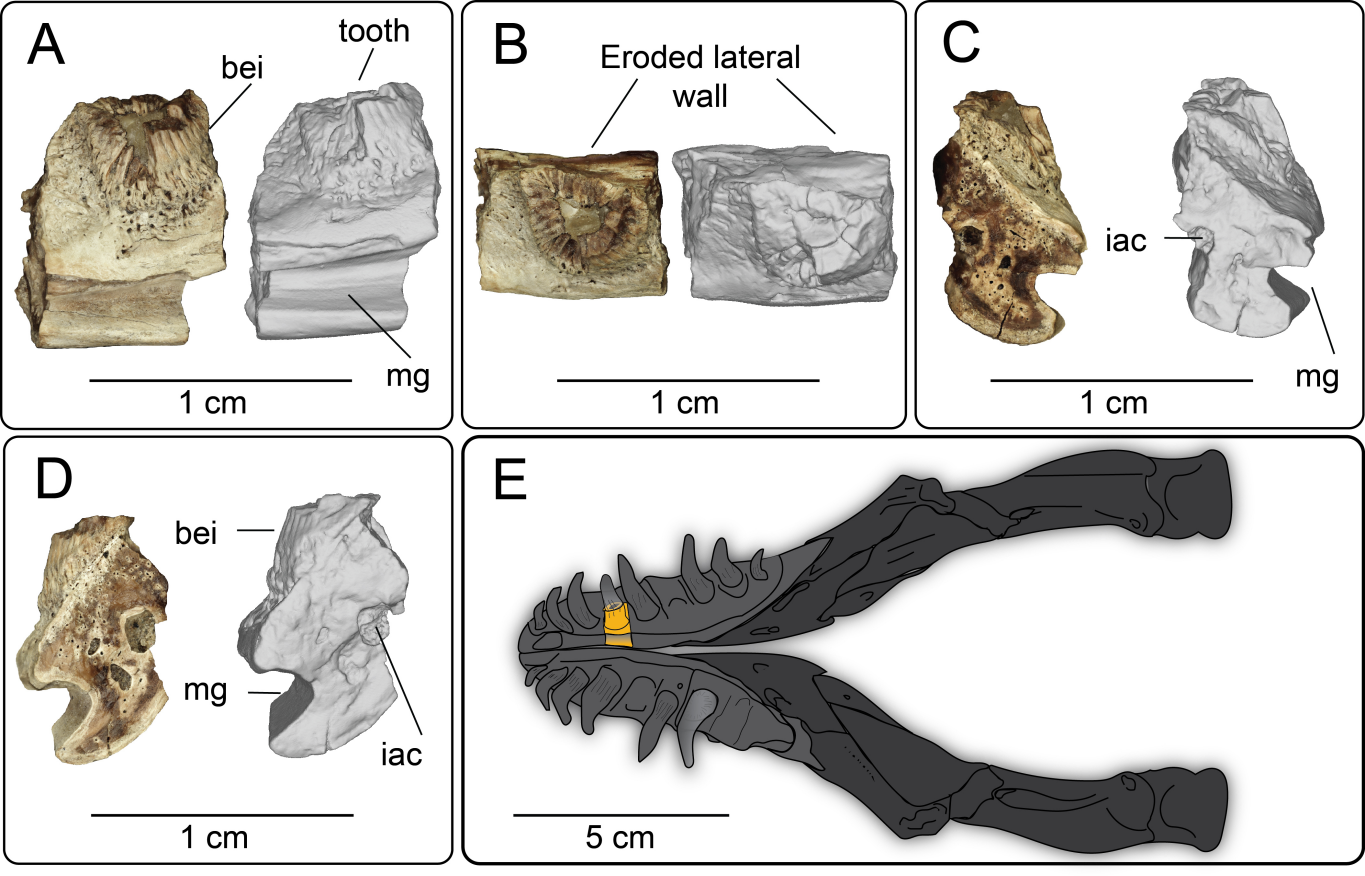
Photographs (left side of panels) and rendered CT scans (right side of panels) of the right dentary fragment UMNH VP 16266.6 (*Bolg amondol)* in (A) medial, (B) dorsal, (C) anterior and (D) posterior views. (E) Inferred placement of the element in a schematic reconstruction of the mandibles of *Bolg amondol* in dorsal view. Anatomical abbreviations: bei, basal tooth enamel infoldings; iac, inferior alveolar canal; mg, Meckelian groove.

**Description:** UMNH VP 16266.6 (figure 21) is the medial portion of the middle ramus of the right dentary, preserving one tooth position with the base of its tooth. The Meckelian groove projects ventromedially and narrows anteriorly (figure 21A*–*D) and the toothrow at this position lacks a prominent subdental shelf (figure 21A). The base of the preserved tooth is attached to the dentary in a modified pleurodont fashion, with an expanded tooth base possessing the plicidentine condition (figure 21A) which results in prominent basal folds in the enamel (figure 21A*–*D).

**Figure 22.**
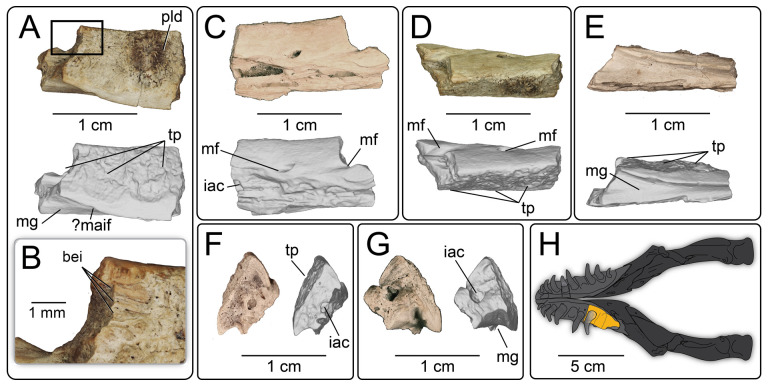
Photographs and rendered CT scans of the left dentary fragment UMNH VP 16266.5 (*Bolg amondol*) in (A) medial, (C) lateral, (D) dorsal, (E) ventral, (F) anterior and (G) posterior views. (B) Close-up image showcasing the preserved basal enamel infoldings. (H) Inferred placement of the element in a schematic reconstruction of the mandibles of *Bolg amondol* in dorsal view. Anatomical abbreviations: bei, basal tooth enamel infoldings; iac, inferior alveolar canal; mf, mental foramen; mg, Meckelian groove; pld, plicidentine; tp, tooth position.

UMNH VP 16266.5 (figure 22) is the posterior portion of the left dentary preserving two complete tooth positions and one partial tooth position; a small portion of the tooth is preserved in the anterior of the three positions. The lateral surface of the dentary is partially eroded (figure 22C,F) though the dorsal portion is present. Two mental foramina are present, one fully preserved (figure 22C) and the posterior foramen partially preserved. In ventral view (figure 22E), the Meckelian groove is shallower than in UMNH VP 16266.6 and widens posteriorly. In medial view (figure 22A), the dental shelf narrows posteriorly, with the ventral margin curving dorsally, forming a ventrally convex margin that overhangs the Meckelian groove. The ventromedial apex of this margin is pointed and almost blade-like in anterior and posterior view. Tooth attachment is modified pleurodont, with the base of the anterior tooth preserved (figure 22A) highlighting the plicidentine condition in the pulp cavity. The posterior-most fragmentary tooth position preserves basal enamel infoldings (figure 22A,B).

**Figure 23.**
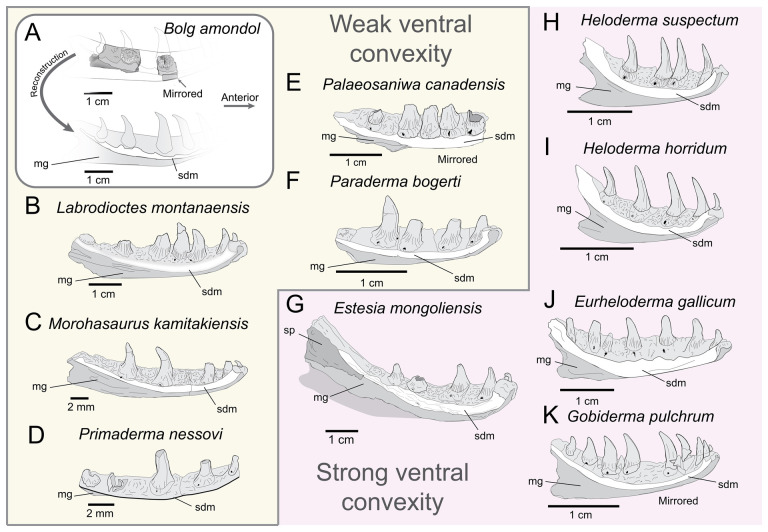
Comparisons of the medial subdental margin (depicted in white) on the dentaries of monstersaurian taxa. (A) Reconstructed left dentary of *Bolg amondol*, upper Campanian Kaiparowits Formation (Fm), Utah, USA, using a mirrored image of the right dentary fragment UMNH VP 16266.6. (B) Left dentary ANSP 18664 (holotype, *Labrodioctes montanaensis*, upper Campanian Oldman Fm, Alberta,Canada, modified from [7]. (C) Left dentary span >MNHAH D1049652 (holotype, *Morohasaurus kamitakiensis*, lower Albian Ohyamashimo Formation, Japan, modified from [15]. (D) Left dentary OMNH 27750 (referred specimen, *Primaderma nessov*i, Cenomanian Mussentuchit Member, Cedar Mountain Fm, Utah, USA, modified from [10]. (E) Right dentary (reflected for comparison) TMP 1989.121.0001 (referred specimen, *Palaeosaniwa canadensis*, upper Campanian Oldman Fm, Alberta, Canada, modified from [7]. (F) Left dentary UALVP 29845 (referred specimen, *Paraderma bogerti*, Maastrichtian Scollard Fm, Alberta, Canada, modified from [7]. (G) Left dentary IGM 3/14 (holotype, *Estesia mongoliensis*, Campanian Djadokhta Fm, Mongolia, modified from [5]. (H) Left dentary TMM-M 9001 (referred specimen, *Heloderma suspectum*, modern, modified from [19]. (I) Left dentary SDSNH 59469 (referred specimen, *Heloderma horridum*, modern, modified from [19]. (J) Right dentary (reflected for comparison) BMNHR 3487 (referred specimen, *Eurheloderma gallicum*, Eocene Phosphorites du Quercy, France, modified from [16]. (K) Left dentary IGM 3/55 (referred specimen, *Gobiderma pulchrum*, Campanian Djadokhta Fm, Mongolia, modified from [12].

**Comparison:** The fragmentary right segment UMNH VP 16266.6 was mirrored to reconstruct a composite left dentary of UMNH VP 16266 in medial view (figure 23). The inferred position of UMNH VP 16266.6 relative to UMNH VP 16266.5 is based on the curvature of the medial subdental margin in medial view, as well as the relative thickness of the smooth, medial surface of the subdental margin among the two elements. This smooth medial surface is dorsoventrally thickened in the posterior specimen (figures 22A and 23A). The positioning of the anterior dentary specimen UMNH VP 16266.6 in the reconstruction is based on the assumption that this medial margin is ventrally curved, as in all other monstersaurian taxa (figure 23). However, the more shallow curvature of the medial subdental margin suggests that UMNH VP 16266 possesses a more elongate dentary than those of Helodermatidae (e.g. *Eurheloderma gallicum* [16]; figure 23J; and *Heloderma* [19]; figure 23H,I) and *Estesia* [5] (figure 23G), and more similar to *Primaderma nessovi* [10] (figure 23D), *Labrodioctes montanaensis* [7] (figure 23B), two dentaries referred to *Palaeosaniwa canadensis* [7] (figure 23E), a dentary referred to *Paraderma bogerti* [7] (figure 23F) and *Morohasaurus kamitakiensis* [15] (figure 23C). An elongate dentary is also consistent with our inferred morphology of the snout based on the preserved maxilla of UMNH VP 16266, which was probably less anteroposteriorly ‘blunted’ than observed in the Helodermatidae (*Eurheloderma gallicum* [16] and *Heloderma*) and *Estesia mongoliensis* [6].

The medial subdental margin in the posterior specimen (UMNH VP 16266.5) is particularly comparable with other monstersaur specimens with more elongate dentaries (figure 23A*–*F). The curvature of the posterior medial subdental margin on UMNH VP 16266.5 is overall similar to *Labrodioctes montanaensis* (figure 23B) [7], *Morohasaurus kamitakiensis* (figure 23C) [15], *Primaderma nessovi* (figure 23D) [10], dentaries referred to *Palaeosaniwa canadensis* (figure 23E) [7], but more strongly curved dorsally than a dentary referred to *Paraderma bogerti* (figure 23F) [7]. The ventral edge of the medial subdental margin of UMNH VP 16266 slightly overhangs the Meckelian groove, forming a blade-like ventral projection that is visible in posterior view. This differs from the blunted, robust medial subdental margin on *Labrodioctes montanaensis* [7], as well as *Morohasaurus kamitakiensis* [15]. The overhanging, blade-like subdental margin is most similar to two dentary specimens referred to *Palaeosaniwa canadensis* by Gao & Fox [7] on the basis of size and chronostratigraphic overlap with the holotype vertebra described by Gilmore [31].

### 4.3. Axial skeleton

**Figure 24.**
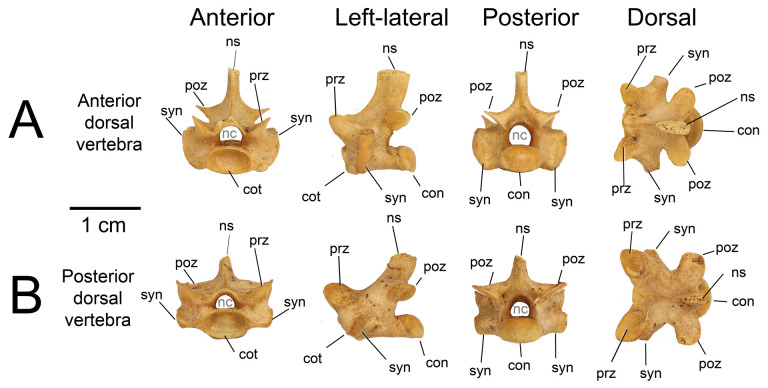
Morphological comparisons between a monstersaur individual’s anterior and posterior dorsal vertebrae (LACM 163584 *Heloderma horridum*). (A) Anterior dorsal vertebra in anterior, left-lateral, posterior and dorsal views. (B) Posterior dorsal vertebra in anterior, left-lateral, posterior, and dorsal views. Anatomical abbreviations: con, posterior condyle; cot., cotanterior cotyle; nc, neural canal; ns, neural spine; poz, postzygapophysis; prz, prezygapophysis; syn, synapophysis.

Nine partial presacral vertebrae and four partial caudal vertebrae are described here. In addition to these elements, numerous fragments of vertebral neural arches (*n* = 16), zyagapophyses (*n* = 12), anterior central cotyles (*n* = 4) and posterior central condyles (*n* = 6) were collected, some of which are figured here. Additionally, we have tentatively identified three rib fragments, which are too incomplete to merit description and discussion (see electronic supplementary material). All preserved vertebrae exhibit a procoelous centrum with a moderately oblique articulating condylar surface. No precondylar constriction is observed on any of the centra, a trait present in extant and fossil varanids [76]. The shape of the condyles are dorsoventrally compressed ovoids in posterior view. Although most of the neural spines are missing, the morphology of the preserved portions indicate that the neural spines were anteroposteriorly narrow, with presumably delicate, tall posterior ends based on the most complete specimens. Preserved dorsal vertebrae of UMNH VP 16266 originate from anterior, middle and posterior sections of the dorsal vertebral column. Here, we provide a comparative figure (figure 24) with anterior and posterior dorsal vertebrae of the extant *Heloderma horridum* (specimen LACM 163584) to distinguish between anterior and posterior dorsal vertebrae in UMNH VP 16266. Compared with posterior dorsal vertebrae, the anterior dorsal vertebrae in *Heloderma* exhibit: (i) a neural canal with a relatively larger diameter compared with the size of the vertebra; (ii) an anterolaterally expanded neural spine; (iii) a dorsoventrally expanded synapophysis for articulation with thoracic rib; (iv) a relatively smaller pre- and post-zygapophysis; and (v) an anteroposteriorly shortened neural arch in lateral and dorsal view compared with posterior dorsal vertebrae. See §4.7.1 for morphological comparison of UMNH VP 162666 dorsal vertebrae among monstersaur taxa.

#### 4.3.1. Posterior cervical/anterior thoracic vertebrae

**Figure 25.**
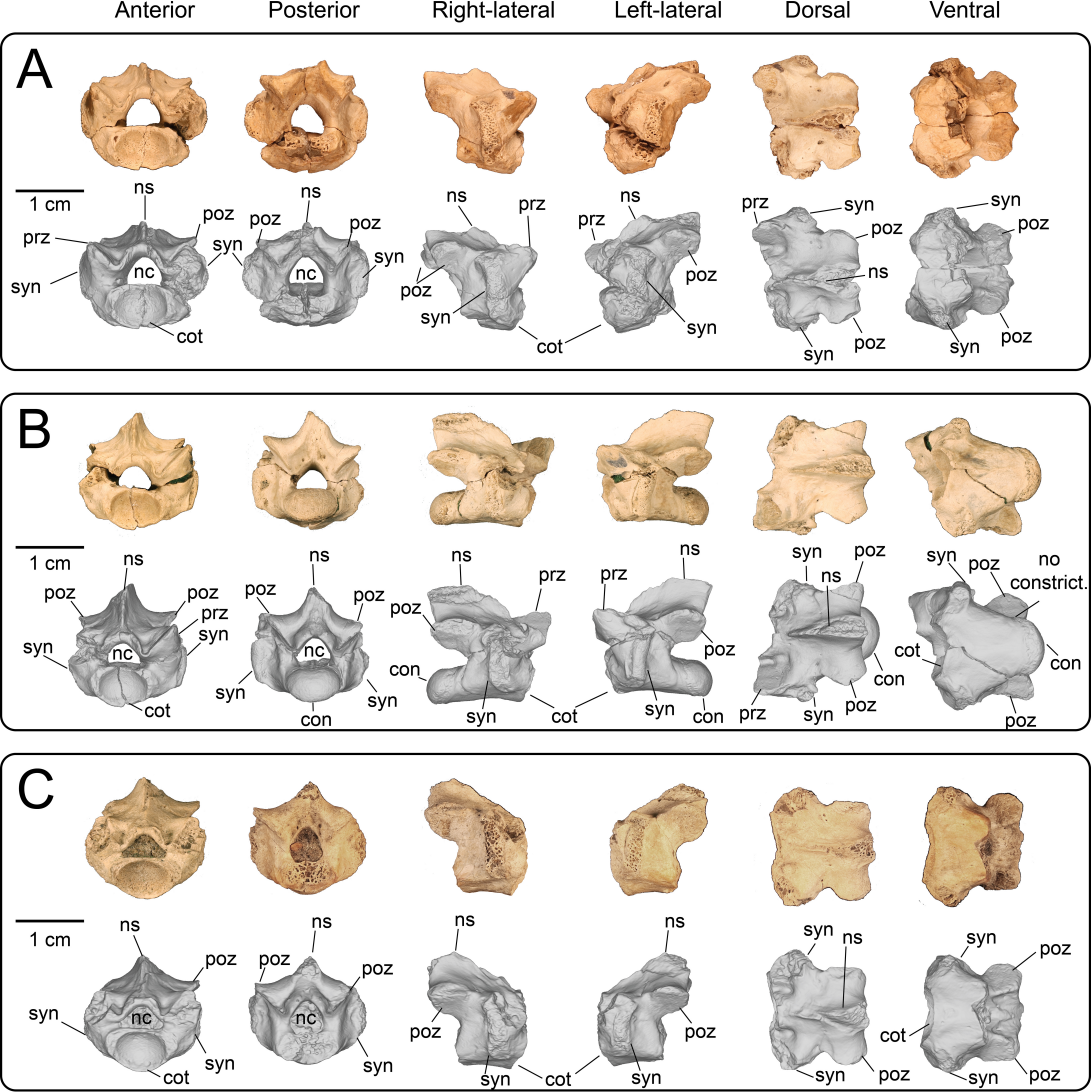
Photographs (top of panels) and rendered CT scans (bottom of panels) of anterior dorsal vertebrae of UMNH VP 16266 (holotype, *Bolg amondol*). (A) Anterior dorsal vertebra UMNH VP 16266.11 in six anatomical views. (B) Anterior dorsal vertebra UMNH VP 16266.12 in six anatomical views. (C) Anterior dorsal vertebra UMNH VP 16266.9 in six anatomical views. Anatomical abbreviations: con, posterior condyle; cot, anterior cotyle; nc, neural canal; no constrict., no precondylar constriction on the centrum; ns, neural spine; poz, postzygapophysis; prz, prezygapophysis; syn, synapophysis.

**Description:** UMNH VP 16266.11 (figure 25A), UMNH VP 16266.12 (figure 25B) and UMNH VP 16266.9 (figure 25C) represent either posterior cervical or anterior dorsal vertebrae, due to the anteroposterior and mediolateral shortening of the neural arch in dorsal view, the relatively larger diameter of the neural canal compared with the size of the vertebra, the steep rise of the neural spine posterior to the anterior terminus of the neural canal, and the subcircular shape of the anterior cotyle of the centrum in anterior view (more posterior cotyles are more dorsoventrally compressed). Additionally, these specimens possess dorsoventrally tall synapophyses that are posteriorly hooked in lateral view at the dorsal margin for articulation with posterior cervical and/or anterior thoracic ribs. The synapophysis of UMNH VP 16266.11 is slightly more dorsoventrally expanded in lateral view than those of UMNH VP 16266.12 and UMNH VP 16266.9. The shape of the neural canal is dorsally arched in anterior and posterior views.

#### 4.3.2. Thoracic vertebrae

**Figure 26.**
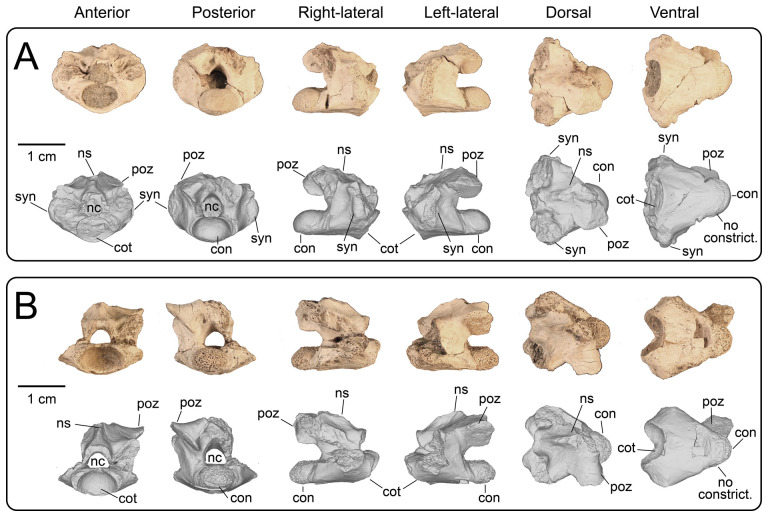
Photographs (top of panels) and rendered CT scans (bottom of panels) of middle dorsal vertebrae of UMNH VP 16266 (holotype, *Bolg amondol*). (A) Middle dorsal vertebra UMNH VP 16266.16 in six anatomical views. (B) Middle dorsal vertebra UMNH VP 16266.10 in six anatomical views. Anatomical abbreviations: con, posterior condyle; cot, anterior cotyle; nc, neural canal; no con, no precondylar constriction on the centrum; ns, neural spine; poz, postzygapophysis; prz, prezygapophysis; syn, synapophysis.

**Description:** UMNH VP 16266.16 (figure 26A) and UMNH VP 16266.10 (figure 26B) are thoracic vertebrae based on the anteroposterior shortening of the neural arch in dorsal view, as well as the dorsoventrally tall synapophyses for articulation with posteroventrally directed posterior cervical and/or anterior thoracic ribs. The neural canals of both vertebrae are dorsally arched in anterior and posterior view. The synapophyses on UMNH VP 16266.16 are slightly less dorsoventrally extensive than on UMNH VP 16266.9, indicating a potentially more posterior position in the vertebral column. UMNH VP 16266.10 (figure 26B) is hypothesized here to be located more posterior to UMNH VP 16266.16 (figure 26A), based on the dorsoventrally compressed ovoid shape of the anterior cotyle of the centrum (figure 26B) and anteroposterior extension of the neural arch in dorsal view. Neither synapophysis is preserved on the specimen and therefore we cannot discern the nature of rib attachments.

#### 4.3.3. Lumbar vertebrae

**Figure 27.**
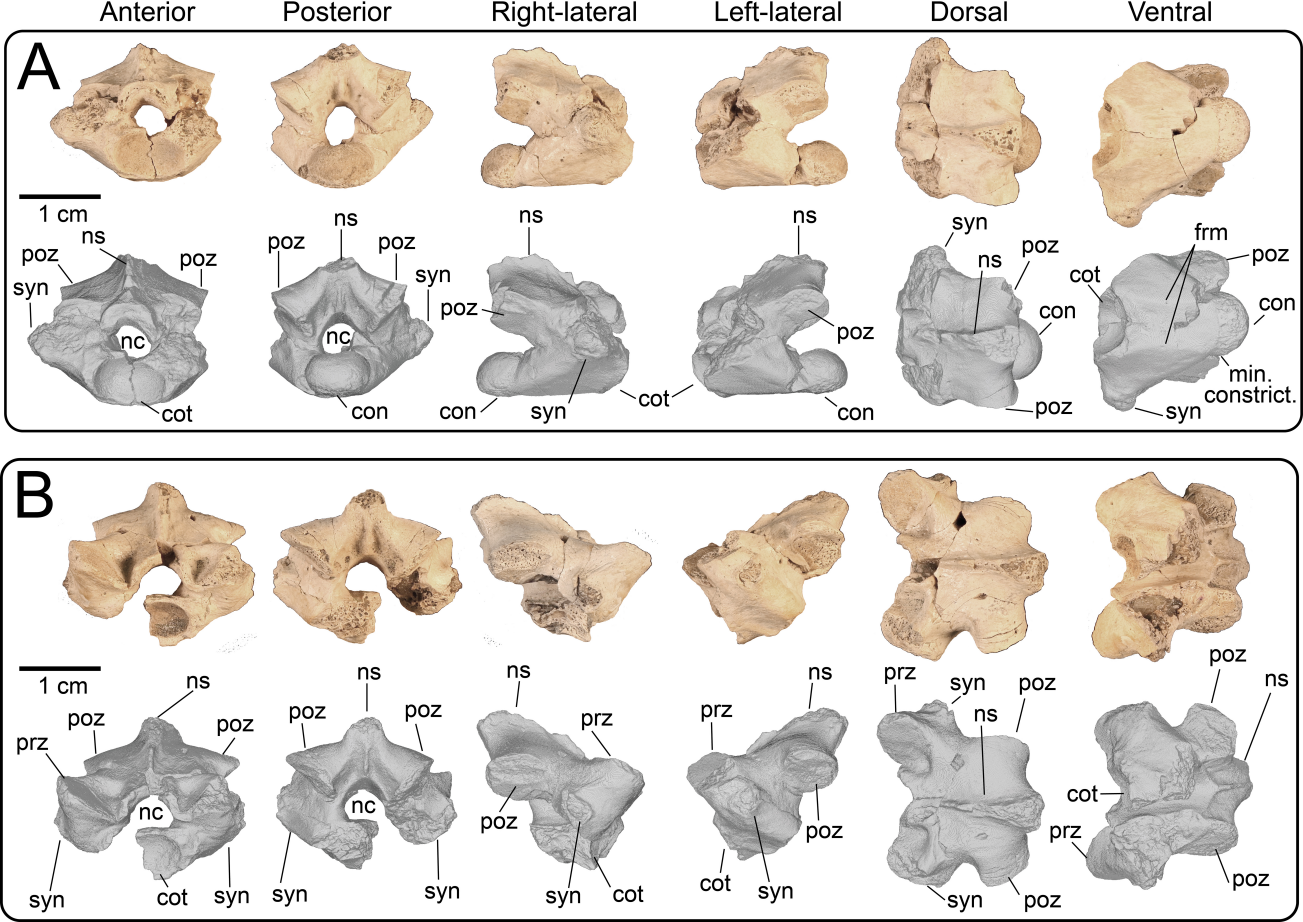
Photographs (top of panels) and rendered CT scans (bottom of panels) of posterior dorsal vertebrae of UMNH VP 16266 (holotype, *Bolg amondol*). (A) Posterior dorsal vertebra UMNH VP 16266.15 in six anatomical views. (B) Posterior dorsal vertebra UMNH VP 16266.13 in six anatomical views. Anatomical abbreviations: con, posterior condyle; cot, anterior cotyle; frm, foramina; min con, minimal precondylar constriction on the centrum; nc, neural canal; ns, neural spine; poz, postzygapophysis; prz, prezygapophysis; syn, synapophysis.

**Description:** UMNH VP 16266.15 (figure 27A) and UMNH VP 16266.13 (figure 27B) probably represent the two posteriormost dorsal vertebrae in the preserved series. This is primarily evident in: (i) absence of dorsoventrally expanded synapophyses; (ii) anteroposterior and mediolateral expansion of the neural arch in dorsal view; and (iii) anteroposterior reduction of neural spine width in lateral view. On UMNH VP 16266.15, the ventral surface of the centrum possesses two gently sloped medial ridges that run anteroposteriorly, indicating a posterior position more proximal to the sacrum as observed in *Estesia mongoliensis* [1], MOR 792 [8,30] and specimens of extant *Heloderma*. The centrum is only partially preserved in UMNH VP 16266.13, though a ridge is present on the left-lateral side of the centrum, potentially representing the anterior portion of a left medial ridge (figure 27B).

#### 4.3.4. Fragmentary dorsal vertebrae

**Figure 28.**
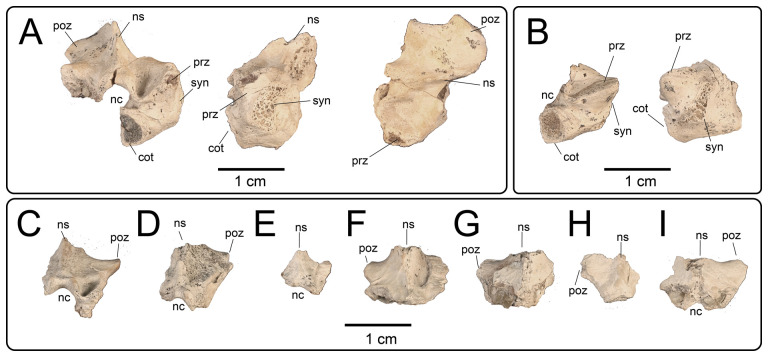
Photographs of dorsal vertebral fragments of UMNH VP 16266 (holotype, *Bolg amondol*). (A) Partial dorsal vertebra UMNH VP 16266.14 in (left to right) anterior, left-lateral and dorsal views. (B) partial dorsal vertebra UMNH VP 16266.17 in (left to right) anterior and left-lateral views. (C–I) Partial dorsal neural arches of UMNH VP 16266 in anterior view. Anatomical abbreviations: cot, anterior cotyle; nc, neural canal; ns, neural spine; poz, postzygapophysis; prz, prezygapophysis; syn, synapophysis.

**Description:** At least nine additional dorsal series vertebrae (figure 28) are represented by partial centra/neural arches (figure 28A,B) and by fragmentary neural arches (figure 28C*–*I). Each of these partial specimens share similar morphology to the neural arches on more complete dorsal vertebrae, confirming their association with UMNH VP 16266.

#### 4.3.5. Proximal and middle caudal vertebrae

**Figure 29.**
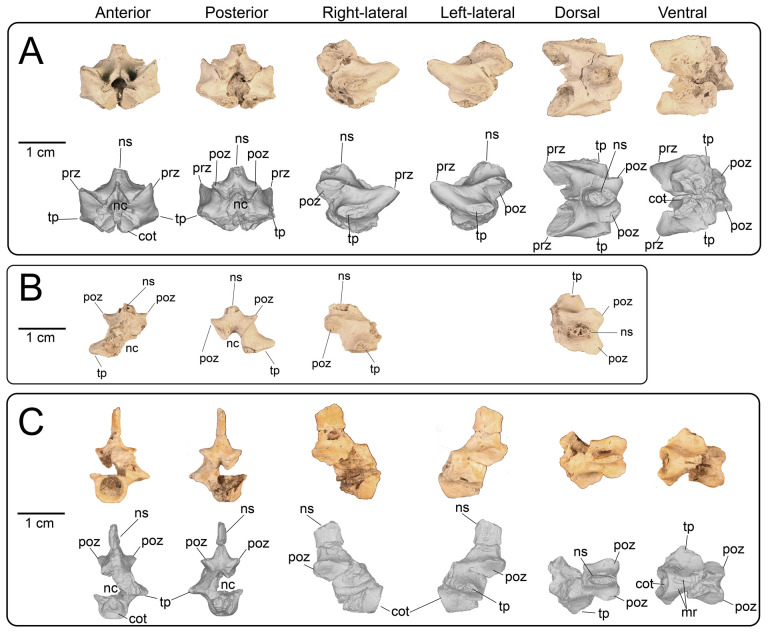
Photographs (top of panels) and rendered CT scans (bottom of panels) of proximal and middle caudal vertebrae of UMNH VP 16266 (holotype, *Bolg amondol*). (A) Proximal caudal vertebra UMNH VP 16266.8 in six anatomical views. (B) Partial proximal caudal vertebra UMNH VP 16266 in four anatomical views. (C) Middle caudal vertebra UMNH VP 16266.28 in six anatomical views. Anatomical abbreviations: con, posterior condyle; cot, anterior cotyle; mr, medial ridge on centrum; nc, neural canal; ns, neural spine; poz, postzygapophysis; prz, prezygapophysis; tp, transverse process.

**Description:** UMNH VP 16266.8 (figure 29A) is from a more proximal position than the other preserved caudal vertebrae and is hypothesized to be a proximal caudal vertebra due to its anteroposteriorly shortened neural arch in dorsal view, posterior constriction of the neural arch in dorsal view, and horizontally oriented bases of broken transverse processes. The posterior portion of the centrum is eroded away. The prezygapophyses are well-developed, whereas the articular surfaces of the postzygapohyses are comparatively less extensive. UMNH VP 16266.27 (figure 29B) represents the dorsal and left-lateral portion of an anterior caudal neural arch, with the base of the left transverse process preserved. In dorsal view, this neural arch is similar in proportions to the neural arch of UMNH VP 16266.8 (figure 29A) and is therefore interpreted to represent a similar position in the caudal vertebral series. UMNH VP 16266.28 is a partial middle caudal vertebra, with the anterior half of the centrum, most of the neural arch, and most of the neural spine preserved (figure 29C). In dorsal view, the neural arch appears to be anteroposteriorly lengthened, although the anterior end and prezygapophyses are not preserved. The neural spine is mediolaterally compressed and most of the height is restricted to the posterior portion of the neural arch.

#### 4.3.6. Distal caudal vertebrae

**Figure 30.**
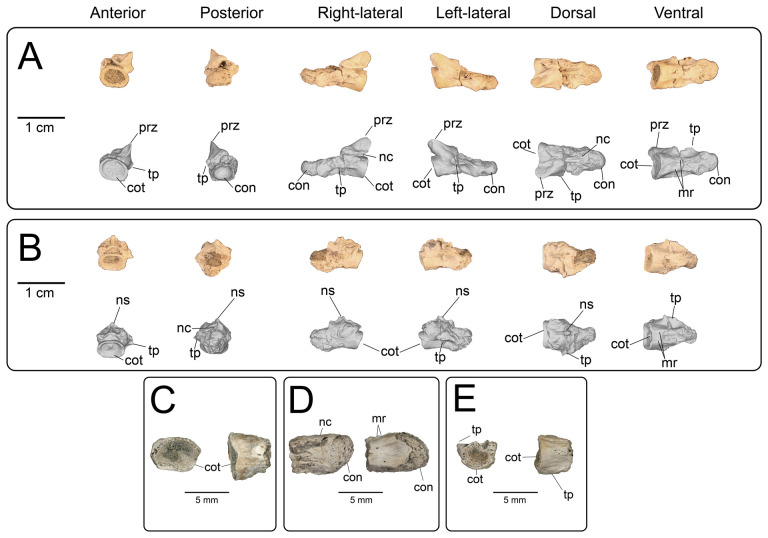
Photographs (top of panels) and rendered CT scans (bottom of panels) of distal caudal vertebrae of UMNH VP 16266 (holotype, *Bolg amondol*). (A) Distal caudal vertebra UMNH VP 16266.29 in six anatomical views. (B) Distal caudal vertebra UMNH VP 16266.30 in six anatomical views. (C–E) Partial distal caudal vertebrae. Anatomical abbreviations: con, posterior condyle; cot, anterior cotyle; mr, medial ridge on centrum; nc, neural canal; ns, neural spine; poz, postzygapophysis; prz, prezygapophysis; tp, transverse process.

**Figure 31.**
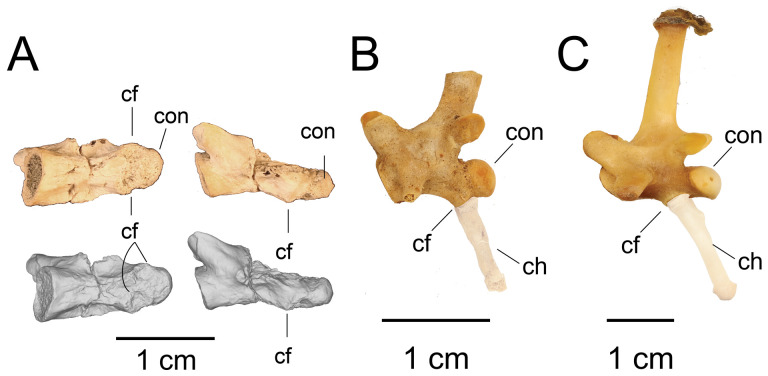
Comparisons of positioning of chevron facets on caudal vertebrae of select anguimorph taxa. (A) *Bolg amondol* distal caudal vertebra UMNH VP 16266.29 in ventral and left-lateral views. (B) LACM 163584 *Heloderma horridum* middle caudal vertebra in left-lateral view. (C) LACM 163943 *Varanus albigularis* middle caudal vertebra in left-lateral view. Anatomical abbreviations: cf, chevron articular facet; con, posteriorarticular condyle.

**Description:** UMNH VP 16266.29 and UMNH VP 16266.30 are incomplete distal caudal vertebrae (figure 30A,B). UMNH VP 16266.29 is represented by an almost complete centrum and a complete left prezygapophysis. On the ventral surface of the centrum, abutting the posterior condyle, are two small, gently concave surfaces. These are interpreted to be articulation facets for the chevron. The positioning of these facets is taxonomically distinguishable between *Heloderma* and *Varanus* (figure 31B,C), with the chevron facets for *Heloderma* abutting the surface of the condyle (as observed in UMNH VP 16266.29, figure 31A) and the chevron facets in *Varanus* completely separated from the condyle and located more anterior on the ventral surface of the centrum (figure 31C). UMNH VP 16266.30 is represented by the anterior half of a centrum with a complete anterior neural arch. The zygapophyses, neural spine and posterior centrum are missing.

**Figure 32.**
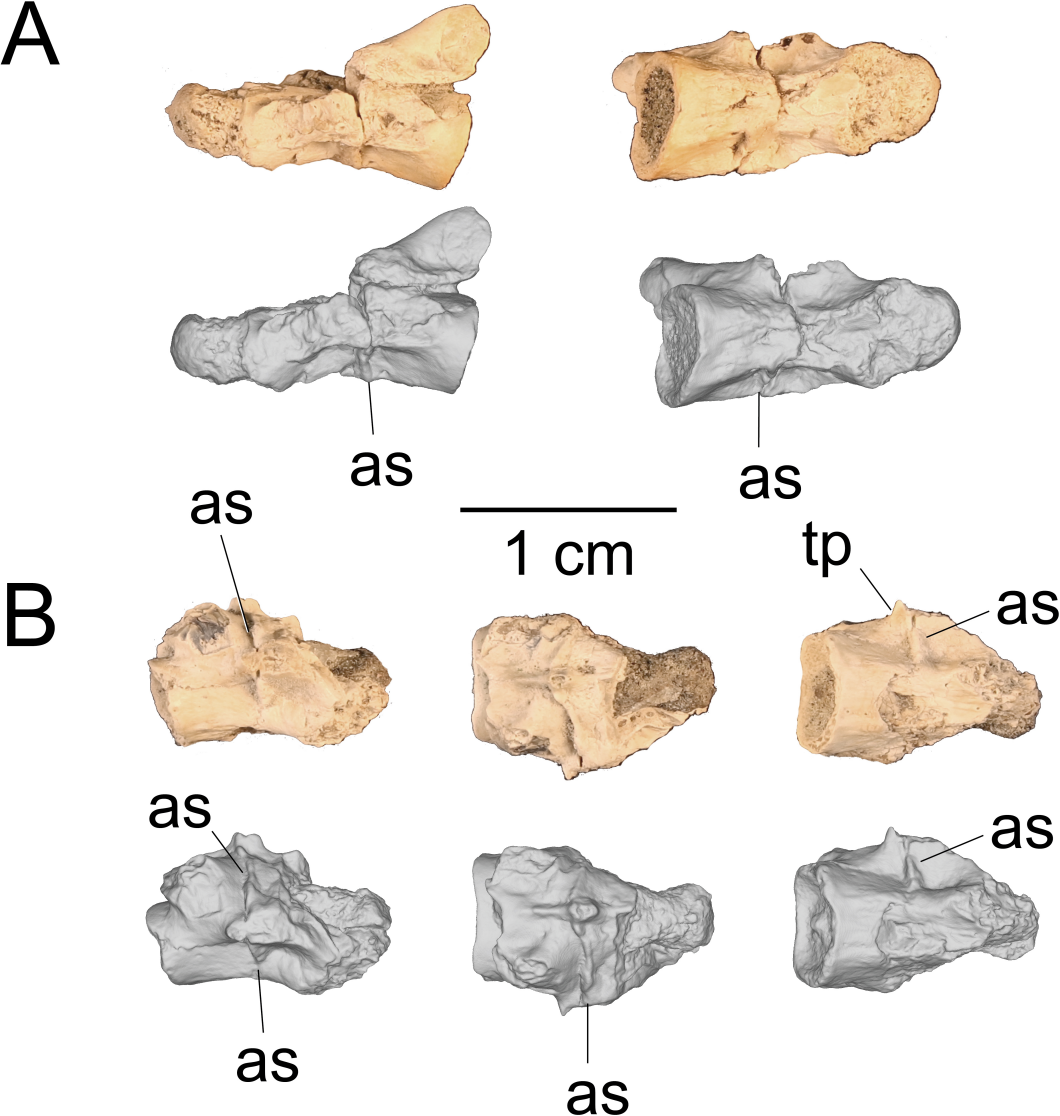
Distal caudal autotomy in *Bolg amondol*. (A) Autotomy septum preserved on distal caudal vertebra UMNH VP 16266.29, visible in right-lateral and ventral views. (B) Autotomy septum preserved on distal caudal vertebra UMNH VP 16266.30, visible in left-lateral, dorsal and ventral views. Anatomical abbreviations: as, autotomy septum; tp, transverse process.

**Figure 33.**
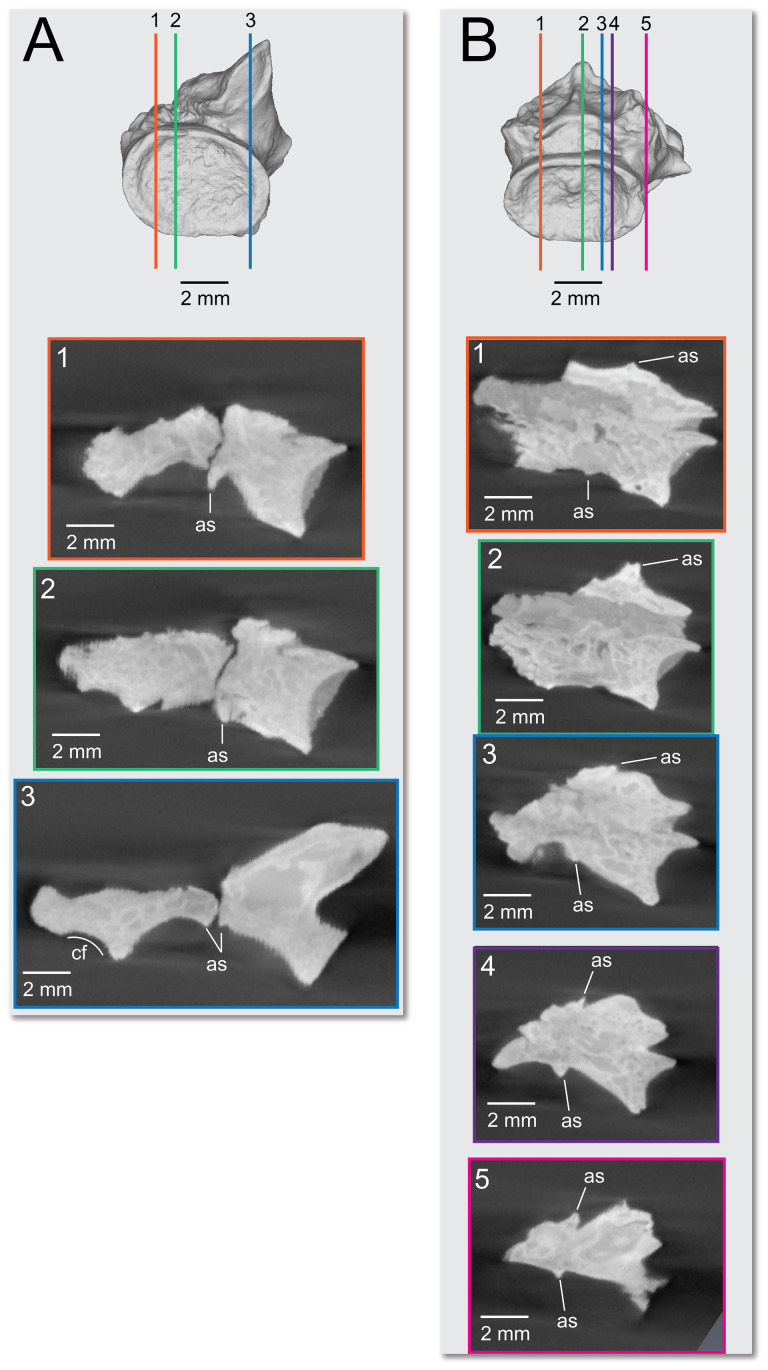
Sagittal CT slices of autotomy septa in (A) distal caudal vertebra UMNH VP 16266.29 and (B) distal caudal vertebra UMNH VP 16266.30. Location of slices indicated by coloured vertical lines in (A) and (B). Anatomical abbreviations: as, autotomy septum; cf, chevron facet.

On the preserved transverse processes of UMNH VP 16266.29 and UMNH VP 16266.30 are distinct, transverse ridges oriented perpendicular to the long axis of the vertebral centrum (figure 32). This ridge is visible across the preserved central surface of UMNH VP 16266.30 (figure 32A) and the ridges are prominently visible in CT slices of both specimens (figure 33). These are interpreted as caudal vertebral autotomy septa [77–79]. Early in a lizard individual’s development, the autotomy septa are usually cartilaginous or connective tissue that allow for that individual to voluntarily shed its tail during acute moments of stress (e.g. attempted predation [77]). Historically, workers have thought that these connective tissues gradually become replaced by bone through development [77,78], while the plane of weakness within the vertebra fuses and autotomy is less relied upon by adult individuals. However, newer research shows that some species of lizard retain the ability to autotomize in adulthood [80], even if ossification does occur in some vertebrae. Obvious internal planes of weakness are not visible in the CT slices of either UMNH VP 16266.29 or UMNH VP 16266.30, but it is likely that the anterior and posterior halves of UMNH VP 16266.29 broke along the autotomy plane (figure 33A, slice 3).

The presence of autotomy planes on UMNH VP 16266 is an important discovery, because it represents the earliest occurrence of this condition in the evolutionary history of Monstersauria. *Heloderma* does not possess autotomy planes at any developmental stage [77–79], and autotomy planes are not observed on the caudal vertebrae of UCMP 118497 [8], an associated skeleton from the Maastrichtian Hell Creek Formation of Montana referred to *Palaeosaniwa canadensis* [8]. Distal caudal vertebrae have not yet been described for *Gobiderma* [2,12] or *Estesia* [1,5,6,9], while caudal vertebrae have not yet been reported for any other monstersaur taxa. However, the presence of caudal autotomy in a Late Cretaceous member of the group suggests that the condition was probably plesiomorphic in the group.

#### 4.3.7. Fragmentary caudal vertebrae

**Description:** UMNH VP 16266.31, UMNH VP 16266.32 and UMNH VP 16266.33 are three incomplete distal caudal centra (figure 30C*–*E). UMNH VP 16266.31 and UMNH VP 16266.33 (figure 30C*–*E) are represented by anterior cotyles of the caudal centra, while UMNH VP 16266.32 (figure 30D) is represented by the posterior condyle; the neural arches of all are missing. The morphology of the haemopophyses of all caudal vertebrae in UMNH VP 16266 is unknown, as the posterior portions of the centra are not preserved well enough to discern the nature of attachment to chevrons.

### 4.4. Appendicular skeleton

#### 4.4.1. Scapulocoracoid

**Figure 34.**
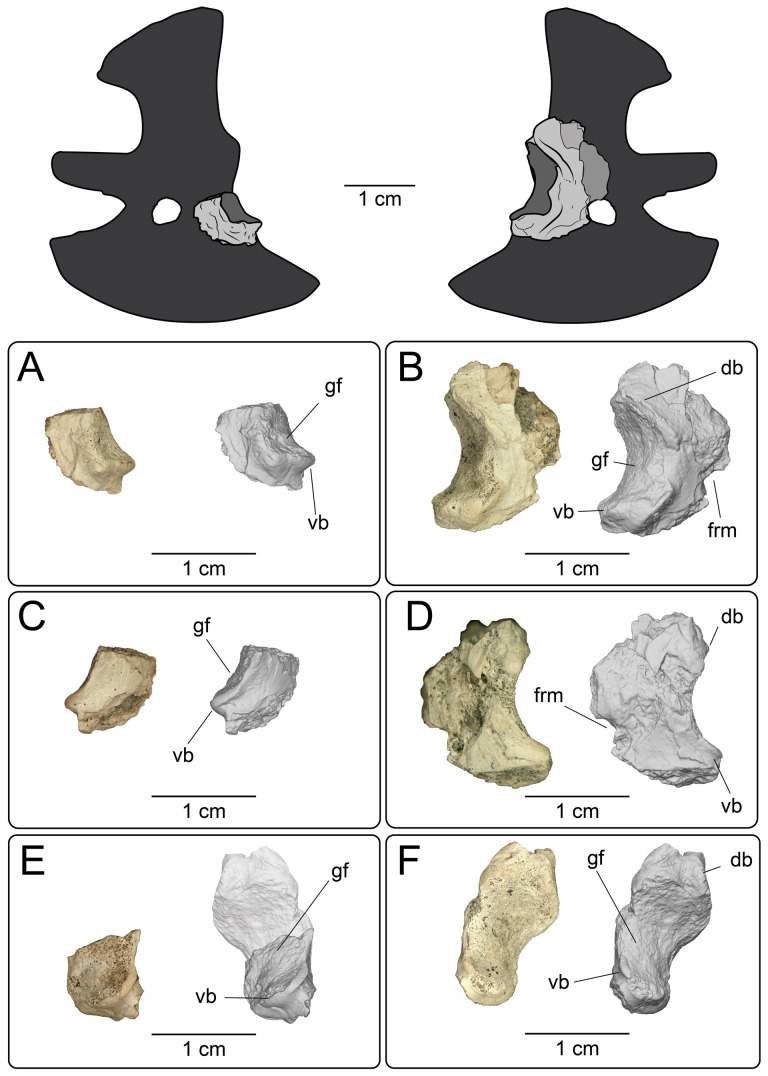
Photographs (left side of panels) and rendered CT scans (right side of panels) of the left and right scapulocoracoid of UMNH VP 16266 (holotype, *Bolg amondol*). Top: reconstructed positions of recovered elements within the pectoral girdle of *Bolg amondol*, modified from those of *Gobiderma pulchrum* [12]. (A) Left partial glenoid fossa UMNH VP 16266.34 in lateral view. (B) Right glenoid fossa UMNH VP 16266.35 in lateral view. (C) Left glenoid fossa in medial view. (D) Right glenoid fossa in medial view. (E) Left glenoid fossa in posterolateral view. Faded silhouette is a mirrored image of the rendered CT scan of the right glenoid fossa. (F) Right glenoid fossa in posterolateral view. Anatomical abbreviations: db, dorsal buttress; frm, foramen; gf, glenoid fossa; vb, ventral buttress.

**Description and comparison:** Portions of the glenoid fossae from both the left (UMNH VP 16266.34) and right (UMNH VP 16266.35) scapulocoracoids are preserved (figure 34). The morphology of the right glenoid fossa (figure 34B,D,F) shows that the scapulocoracoid is fused into a single element, as observed in *Gobiderma pulchrum* [12] and *Heloderma horridum* (e.g. specimen UF:Herp:153328). Relative contributions to the glenoid fossa from the coracoid and scapula are unclear because of the incomplete nature of the specimens and due to the lack of clarity on where the fusion between scapula and coracoid occurs. The glenoid fossa is oriented posterolaterally, as in most extant squamates. Both the dorsal and ventral buttresses are robust.

#### 4.4.2. Radiale

**Figure 35.**
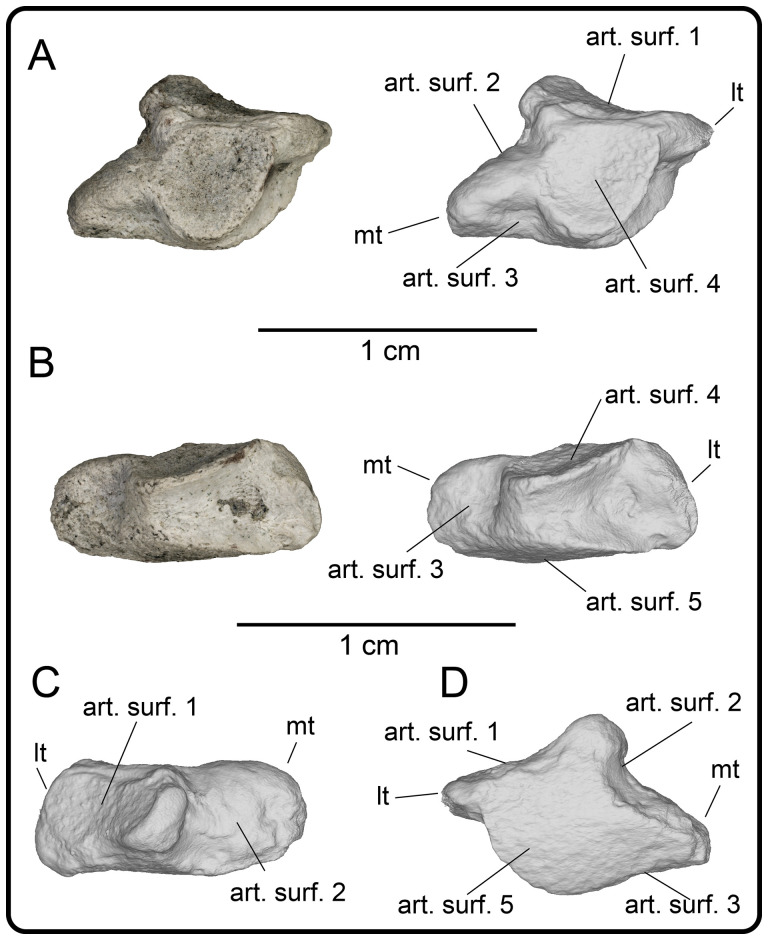
Photographs (left side of panels (A) and (B)) and CT renderings (A–D) of radiale UMNH VP 16266.36 (holotype, *Bolg amondol*). (A) View 1. (B) View 2. (C) View 3. (D) View 4. Anatomical abbreviations: art surf, articular surface; lt, lateral radiale tubercule; mt, medial radiale tubercule.

**Description and comparison:** UMNH VP 16266.36 is a carpal element, probably a radiale based on the presence of what are interpreted as medial and lateral radiale tubercules (figure 35A*–*D). The element compares favourably with the radiale of *Heloderma suspectum* [81] and other anguimorphs such as *Shinisaurus crocodiluus* [82] and *Bahndwivici ammoskius* [83]. The relative size of UMNH VP 16266.36 also supports the identification of this element as a radiale.

#### 4.4.3. Ilium

**Figure 36.**
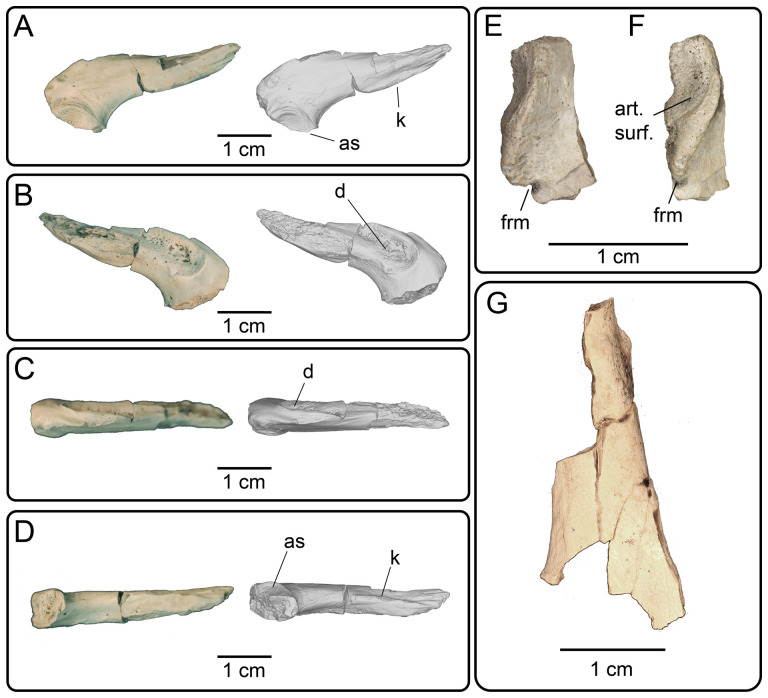
Photographs (left side of panels) and rendered CT scans (right side of panels) of the left ilium UMNH VP 16266.23 (holotype, *Bolg amondol*) in (A) lateral, (B) medial, (C) dorsal and (D) ventral views. (E–F) photographs of a girdle element of UMNH VP 16266.24 in (E) medial/lateral view, and (F) view of articular surface. (G) photograph of a fragmentary limb element of UMNH VP 16266. Anatomical abbreviations: art surf, articular surface; as, acetabular surface; d, depression; frm, foramen; k, keel.

**Description and comparison:** UMNH VP 16266.23 (figure 36A*–*D) is the mostly complete left ilium. The ilium tapers posterodorsally, its blade mediolaterally compressed anteriorly but subcircular dorsally. Ventrolateral to the main, subcylindrical posterior body, the ilium possesses a narrow ventral keel that is less prominent than in *Gobiderma* [83]. In medial view, an elongate anteroposterior depression is present. The orientation of this depression along the long axis of the ilium indicates that it would have been oriented posterodorsally in life, as observed in *Heloderma horridum*.

#### 4.4.4. Girdle element

**Description:** UMNH VP 16266.24 (figure 36E,F) is identified as a girdle element but it is too incomplete for more specific identification. The articular surface is moderately concave and presumably a part of the acetabulum. A small foramen is present below the articular surface.

#### 4.4.5. Limb element

**Description:** Fragments of a limb element are preserved (figure 36G), although limited morphology is available to identify the element. The specimen expands into a relatively wide and flat plane from a subcylindrical shaft.

### 4.5. cf. *Palaeosaniwa canadensis*

DMNH EPV.134394

DMNH EPV.134934 consists of a parietal (DMNH EPV.134394.1) and an associated partial posterior dorsal vertebra (DMNH EPV.134394.2). Both specimens were recovered from DMNH Loc. 5942 in The Blues field area, a bonebed horizon with disarticulated elements of mostly ceratopsid dinosaurs that was excavated from 2019 to 2021. The parietal and vertebra were discovered within 1 m of each other within the same horizon. Over this multi-year excavation, DMNH EPV.134394 was the only squamate specimen recovered from the quarry. The size of both specimens indicates they both belong to a large species of lizard. The preservation (detailed external morphology preserved, similar dark brown colour of permineralized bone) suggest similar postdepositional and diagenetic conditions. Taphonomy of the site, including rip-up clasts, pedogenic carbonate nodules and plant debris, indicates exhumation and redeposition of closely associated individuals of all taxa present in the horizon (two centrosaur ceratopsids, one crocodylian) from an original overbank host deposit in which skeletons were closely associated or articulated. The combination of these factors indicates that DMNH EPV.134394 probably belong to the same individual, and we treat this association as such throughout the rest of the manuscript.

#### 4.5.1. Parietal

**Figure 37.**
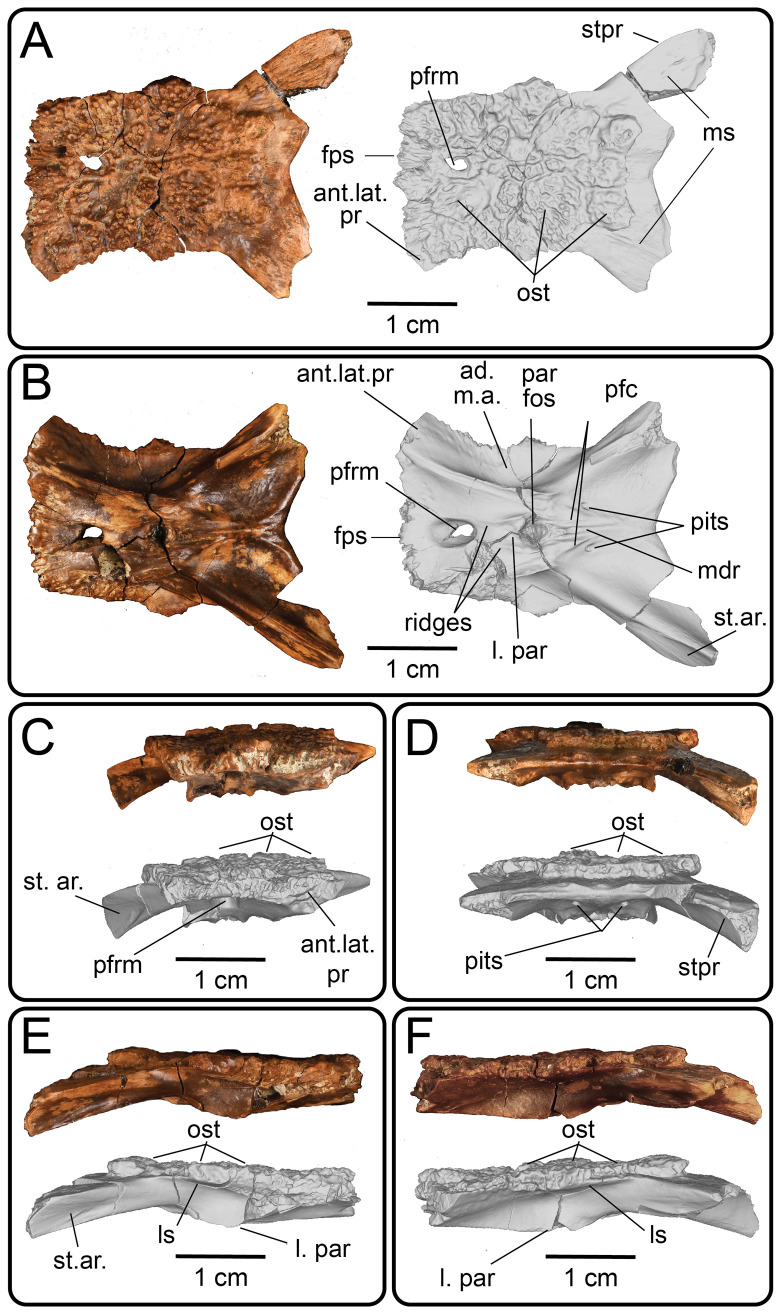
Photographs and rendered CT scans of DMNH EPV.134394.1 (cf. *Palaeosaniwa canadensis*), partial parietal in (A) dorsal, (B) ventral, (C) anterior, (D) posterior, (E) right-lateral and (F) left-lateral views. Anatomical abbreviations: ad.m.a., adductor musculature attachment; ant.lat.pr, anterolateral process; fps, frontoparietal suture; l. par, lamina parietalis; ls, lateral shelf; mdr, midline ridge; ms, musclescarring; ost, osteoderms; par fos, parietal fossa; pfc, postfoveal crest; pfrm, parietal foramen; st. ar., supratemporal articular facet; stpr, supratemporal process.

**Description:** DMNH EPV.134394.1 is a partial parietal preserving the majority of the parietal table, the proximal portion of the left supratemporal process and most of the right supratemporal process (figure 37). The parietal is co-fused into a single, midline element. The frontoparietal suture is transversely oriented (figure 37A) and is preserved along most of the anterior margin of the parietal, showcasing weak interdigitations. The anterolateral processes are broken, however enough of the left anterolateral process is preserved to show that its dorsal surface is medially concave (figure 37C). In ventral view (figure 37B), the ventrolateral surface of the anterolateral process transitions from mediodorsally convex to mediodorsally concave. The overall morphology of the parietal, including the position and shape of the parietal foramen, is nearly identical to DMNH EPV.132910 (see below). The left-lateral margin of the parietal table is almost completely preserved (figure 37D) and is slightly medially concave in dorsal view. The supratemporal processes extend posterolaterally at an obtuse angle from one another and extend slightly ventrally (figure 37D). The dorsal surfaces of the supratemporal processes are flat and broad like those of *Heloderma suspectum* and *Heloderma horridum* [12], *Palaeosaniwa canadensis* [8] and *Gobiderma pulchrum* [2,12], contrasting with the condition observed in many other anguimorphs and the monstersaur *Estesia mongoliensis* in which the dorsal surface of the supratemporal process is very narrow and bladelike [5]. The supratemporal processes are V-shaped in cross-section, their leading edge (the edge adjacent to the posteromedial margin of the supratemporal fenestra) dorsoventrally broadened. A shallow supratemporal articular facet is preserved on the right supratemporal process (figure 37C), extending anteriorly almost the entire length of the process such that the lateral margin of the supratemporal process would have been excluded from the supratemporal fenestra. The parietal fossa is located just anterior to the level where the wide parts of the supratemporal processes approach each other and join the main body of the parietal (ventral to the parietal table; figure 37B). In DMNH EPV.134394, the postfoveal crests (*cristae postfovealis* [64]) extend posteriorly and curve posterolaterally at the posterior margin of the parietal arch, such that each crest is strongly medially concave in ventral view. Between these postfoveal crests lies an additional crest at the midline that runs antero-posteriorly, absent in DMNH EPV. 1 32 910 (see below). The parietal trough is ventrally raised compared with the rest of the ventral surface of the parietal arch. Lateral to the anterior portions of the postfoveal crests are two small posteriorly projecting pits. The cephalic osteoderms are fused to the dorsal surface of the parietal (figure 37A), and are separated by grooves, though not as well-defined as seen in DMNH EPV.132910. Differences between the osteoderm patterns preserved on the two parietals are discussed below in §4.8, whereas broader comparisons of DMNH EPV.134394.1 among other monstersaurian taxa are discussed in §4.9 below.

#### 4.5.2. Dorsal vertebra

**Figure 38.**
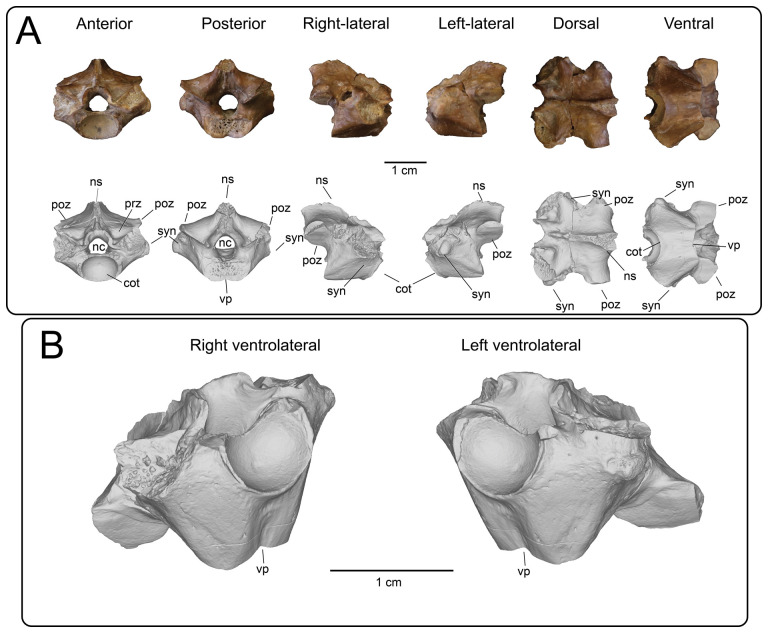
(A) Photographs (top) and rendered CT scans (bottom) of DMNH EPV.134394.2 (cf. *Palaeosaniwa canadensis*), a dorsal vertebra associated with parietal DMNH EPV.134394.1 in six anatomical views. (B) Ventrolateral views of rendered CT scan of DMNH EPV.134394.2 illustrating the deeply excavated ventral pit. Anatomical abbreviations: con, posterior condyle; cot, anterior cotyle; nc, neural canal; ns, neural spine; poz, postzygapophysis; prz, prezygapophysis; syn, synapophysis; vp, ventral pit.

**Description:** DMNH EPV.134394.2 (figure 38) is a posterior dorsal vertebra recovered in association with the parietal. The ventral ridges on the centrum DMNH EPV.134394.2 are more prominent than those observed on the lumbar vertebra of UMNH VP 16266. Between the two ventral ridges at the midline of the ventral surface of the centrum, a shallow depression or ‘groove,’ is present, similar to the position and size of the ventral pits on the holotype specimen of *Palaeosaniwa canadensis* (USNM 10864 [31]) and on MOR 792 [8,30]. The left synapophysis is preserved and is located on the anterior third of the centrum. The synapophysis is only slightly more dorsoventrally elongate than it is anteroposteriorly, it is angled slightly posterodorsally, and it is laterally convex in both anterior and dorsoventral views. The prezygapophyses are broken and missing, whereas the postzygapophyses are mostly preserved. The articular surfaces of the postzygapophyses are oriented more ventrally than laterally. A moderately developed zygosphene is present, and its articular facets are angled dorsolaterally. The neural spine is broken, but its base extends the full anteroposterior length of the neural arch. The preserved anterior end of the neural spine is short and mediolaterally thin, while the base of the broken posterior end expands laterally. Overall, this vertebra is larger in size than the lumbar/posterior thoracic vertebrae in UMNH VP 16266 (*Bolg amondol*), therefore we infer that it belongs to a larger individual. See §4.7.1 below for further discussion.

#### 4.5.3. DMNH EPV.132910, parietal

**Figure 39.**
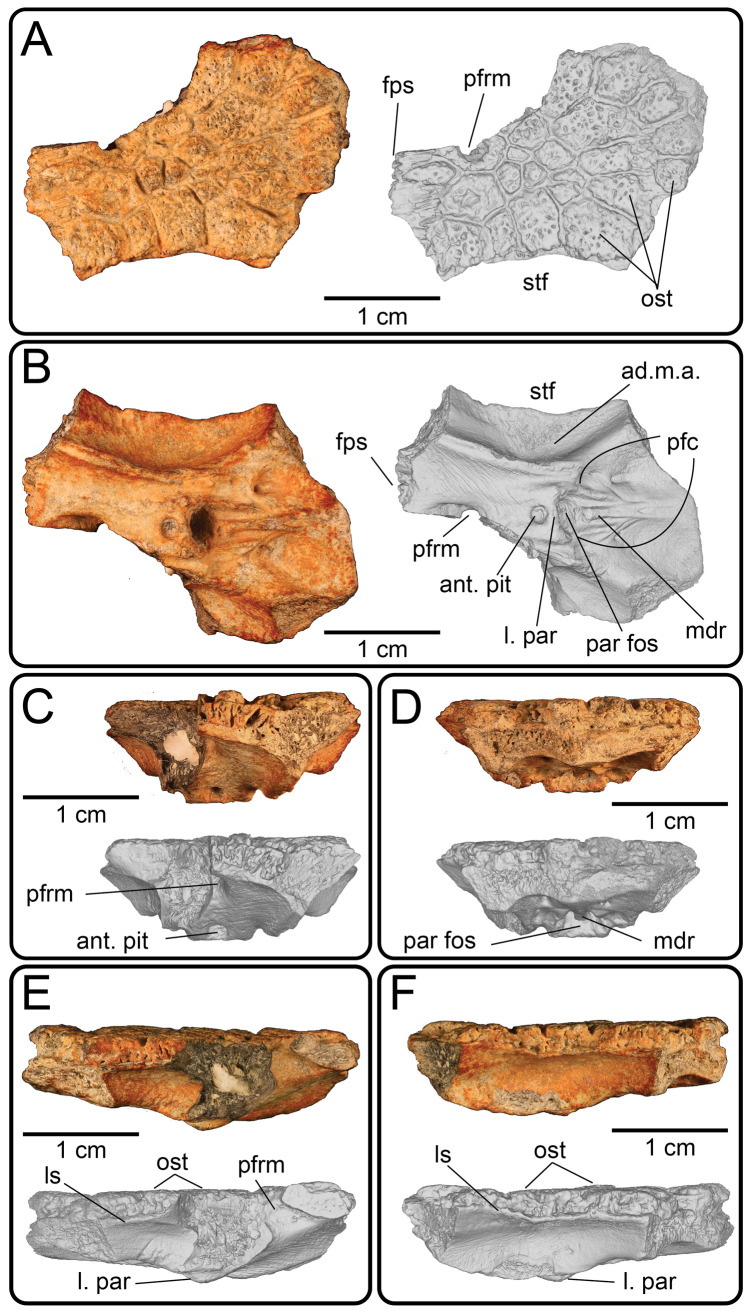
Photographs and rendered CT scans of DMNH EPV.132910 (cf. *Palaeosaniwa canadensis*), a partial parietal in (A) dorsal, (B) ventral, (C) anterior, (D) posterior, (E) right-lateral and (F) left-lateral views. Anatomical abbreviations: ad. m. a., adductor musculature attachment; ant. pit, anterior pit; fps, frontoparietal suture; l. par, lamina parietalis; ls, lateral shelf; mdr, midline ridge; ost, osteoderms; parfos, parietal fossa; pfc, postfoveal crest; pfrm, parietal foramen; stf, supratemporal fenestra.

**Description:** DMNH EPV.132910 (figure 39) is a parietal missing both supratemporal processes and most of the right-lateral side of the parietal table. Prominent osteoderms are fused to the dorsal surface of the parietal. A small portion of the frontoparietal suture is preserved, showcasing weak interdigitations (figure 39A). The parietal entirely encloses the parietal foramen, its external aperture excavated through the fused osteoderms on the dorsal surface of the parietal table. The external aperture of the parietal foramen is about half of the diameter of the internal aperture (figure 39E), and the pineal canal widens anteroventrally. The preserved left-lateral margin of the parietal, which presumably borders the supratemporal fenestra, is weakly concave medially in dorsal view. Discounting the fused osteoderms, which average in dorsoventral thickness at approximately 2.3 mm, the dorsal surface of the left-lateral margin forms a thin lateral shelf, 0.3 mm in dorsoventral thickness (figure 39C,D). The adductor musculature arises from the ventral surface of this shelf. The parietal fossa projects posteriorly and is located just posterior to the level of the apex of the medial curve of the anterior ramus of the *crista crania parietalis* (figure 39B). The anterior margin of the parietal fossa is delimited by the apex of the dorsoventrally inflated *lamina parietalis* [64]. A distinctive, anteroventrally projecting pit borders the anterior margin of the parietal fossa (anterior pit; figure 39B). This feature is unique for lizards more closely related to *Heloderma* than other extant lizards, though a similar structure is present in the anguid *Ophisaurus holeci* [65] and the iguanians *Dipsosaurus dorsalis* and *Sauromalus obesus* [84]. The lateral and posterior margins of the parietal fossa are formed by the postfoveal crests (*cristae postfovealis* [64]) that extend posteriorly and terminate without contacting one another. Between these postfoveal crests lies an additional anteroposterior crest at the midline. The cephalic osteoderms are strongly fused to the dorsal surface of the parietal (figure 39A) and are separated by deep, linear grooves. The dorsal surfaces of the osteoderms are pitted and the osteoderms form elongate pentagonal or hexagonal shapes in dorsal view. Osteoderms at the margins of the parietal are two to three times larger than those that are situated closer to the parietal foramen. See §4.7.3 below for further discussion of similarities and differences of DMNH EPV.132910 and other monstersaur parietals.

### 4.6. Monstersauria indet.

#### 4.6.1. UMNH VP 36373, presacral vertebra

**Figure 40.**
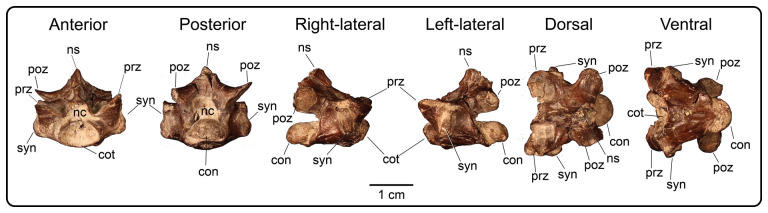
Photographs of UMNH VP 36373 (Monstersauria indet.), an isolated dorsal vertebra, in six anatomical views. Anatomical abbreviations: con, posterior condyle; cot, anterior cotyle; nc, neural canal; ns, neural spine; poz, postzygapophysis; prz, prezygapophysis; syn, synapophysis.

**Description:** UMNH VP 36373 (figure 40) is a well-preserved presacral vertebra missing the distal portion of the neural spine. It is worth noting that a distinct oblique cotylar articulation facet is present around the anterior margin of the central condyle. The synapophyses are dorsoventrally compressed in lateral views, indicating a posterior position in the thoracic series. On the ventral surface of the centrum a shallow ovoid pit is present along the midline of the centrum. This feature is strikingly similar to those found on the dorsal vertebrae of MOR 792 [8,30] but is not as deeply excavated as seen on the holotype specimen of *Palaeosaniwa canadensis* USNM 10864 [31] or on DMNH EPV.134934.2. See §4.7.1 below for morphological comparison of UMNH VP 36373 and other dorsal vertebrae among monstersaur taxa.

### 4.7. Broader comparisons

#### 4.7.1. Dorsal vertebral comparisons

**Figure 41.**
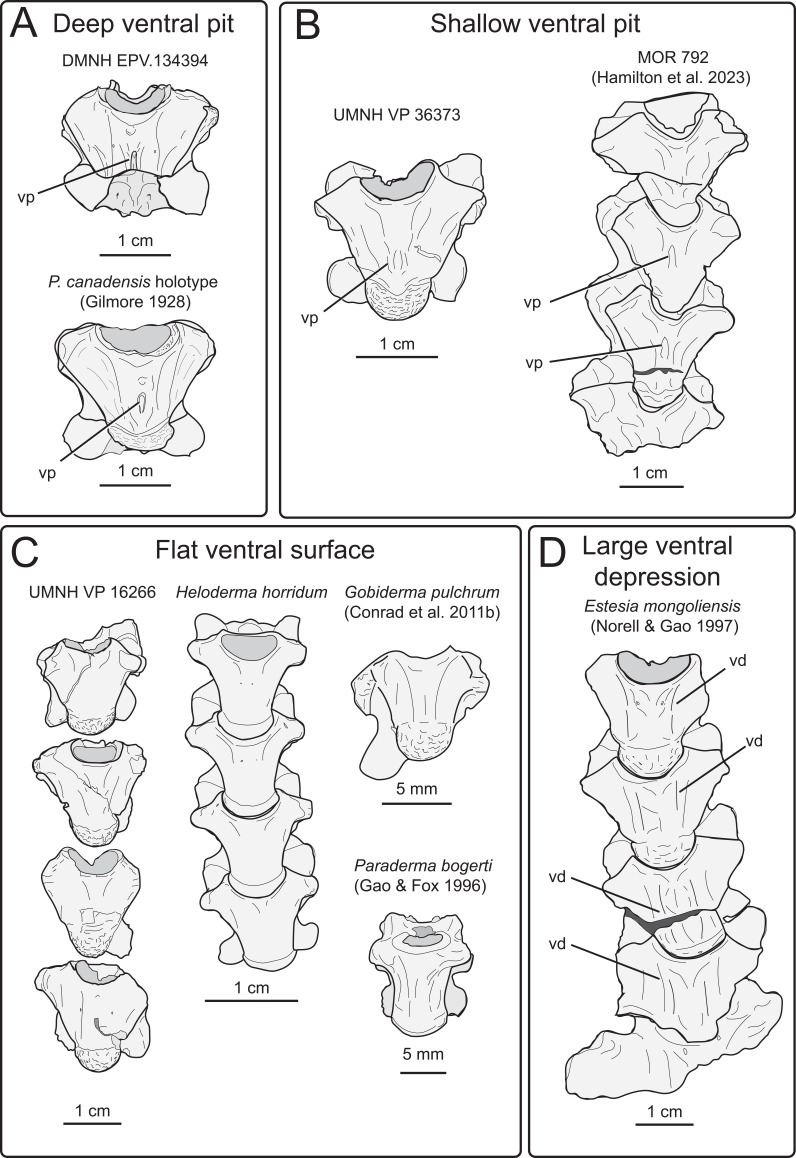
Comparisons of ventral centrum morphotypes on vertebrae referred to monstersaur taxa. (A) Deep pit on the ventral surface of the centrum, as observed on DMNH EPV.134394.2 (top, middle), monstersaur vertebra from the upper Campanian Kaiparowits Formation (Fm) and USNM 10864 (Holotype, *Palaeosaniwa canadensis*, upper Campanian Oldman Fm, modified from [31], pl. XI, fig. 5a). (B) Shallow pit on the ventral surface of the centrum, as observed on UMNH VP 36373 (upper Campanian Kaiparowits Fm, left), and MOR 792 (*Palaeosaniwa canadensis*, Campanian Two Medicine Fm, modified from [30], fig. 5D). (C) Flat ventral surface, as observed on UMNH VP 16266 (left, upper Campanian Kaiparowits Fm), *Heloderma horridum* (middle, modern, UF:Herp:153328), *Gobiderma pulchrum* (IGM 3/905, Campanian Djadokhta Fm, modified from [12], fig. 46B), and *Paraderma bogerti* (modified from [7]). (D) Large depression on the ventral surface of the centrum, as observed on *Estesia mongoliensis* (IGM 3/15, Campanian Djadokhta Fm, modified from [1]). Anatomical abbreviations: vd, ventral depression; vp, ventral pit.

The centra of UMNH VP 16266 and 36373, as well as DMNH EPV.134394.2 all preserve ventral morphological features that can be directly compared with one another, as well as with other monstersaur taxa (figure 41) that together suggest a noteworthy pattern in the biodiversity of monstersaurs from the Kaiparowits Formation. First, DMNH EPV.134394.2 preserves a deeply excavated pit at the midline of the centrum, which is almost identical to the holotype specimen of *Palaeosaniwa canadensis* USNM 10864 (figure 41A) [31]. This character (alongside relatively larger zygapophyses) was used by Gilmore [31] to differentiate the specimen from the vertebrae of the Eocene varanid *Saniwa ensidens* [31,74]. This character was also used as one of the primary bases for the referral of specimen MOR 792 to *Pal. canadensis* in an unpublished PhD dissertation [8,30]; however, the ventral pits on MOR 792 appear to be more shallowly excavated than in USNM 10864 (figure 41B).

Second, almost identical to MOR 792, UMNH VP 36373 also preserved a shallow ovoid ventral pit at the midline of the centrum (figure 41B). This difference in deep excavation (figure 41A) and shallow excavation (figure 41B) is admittedly difficult to ascribe to a single root cause because of the fragmentary monstersaur fossil record. On one hand, we cannot entirely rule out the possibility that these subtle distinctions in pit excavation could be due to generic or species-level taxonomic differences. On the other hand, given the serial variation along the dorsal vertebral column of a single extant monstersaurian individual (*Heloderma horridum* LACM 163584; figure 24), it is possible that the distinctions in the extent of ventral pit excavation are simply due to different sections of the vertebral column being preserved in the described specimens. For instance, the shallower pits on UMNH VP 36373 and MOR 792 could be due merely to a more anterior placement on the vertebral column than DMNH EPV.134394.2 and USNM 10864. This pattern is evident on the posterior dorsal vertebrae of *Estesia mongoliensis* (figure 41D) [1], where the ventral trough on the dorsal centrum becomes gradually more extensive posteriorly. The absence of the midline pit (regardless of excavation extent) on other monstersaurian specimens belonging to a variety of different taxa (e.g. figure 41C,D) suggests that there could still be a taxonomic signal to this feature.

Third, UMNH VP 16266 (*Bolg amondol*; figure 41C) preserves vertebral centra that all lack the ventral pit present on both of the other Kaiparowits specimens (DMNH EPV.134394.2, UMNH VP 36373) as well as those on USNM 10864 and MOR 792. This is more similar to the smooth/flat ventral surfaces seen on *Heloderma horridum* (figure 41C; specimen UF153328) and *Gobiderma pulchrum* (figure 41C; specimen IGM 3/905), as well as dorsal vertebrae from the Maastrichtian Scollard Formation of Alberta, Canada assigned to *Paraderma bogerti* (e.g. UALVP 33903 [7]; figure 41C). As with the other specimens discussed above, we cannot completely rule out the possibility that missing portions of the vertebral column of UMNH VP 16266 possessed a ventral pit. But given current evidence that this feature is not ubiquitous across monstersaurian taxa, and the fact that the four best-preserved centra of UMNH VP 16266 do not preserve evidence of an ovoid ventral pit along the midline (figure 41C), we hypothesize that this feature was not present in *Bolg*, pending recovery of more complete skeletal material.

In sum, the Kaiparowits Formation preserves monstersaur vertebrae with at least two, and potentially three, morphotypes, each comparable with other described monstersaur specimens and taxa from both the Late Cretaceous and Recent records of Asia and North America (figure 41). Based on current evidence, we find that vertebral centrum morphology carries taxonomic signal among monstersaurs, although future reassessment of the character is needed (see §6). We hypothesize that two monstersaurian taxa were present in the Kaiparowits Formation: DMNH EPV.134394.2 and UMNH VP 36373 belong to one taxon, cf. *Palaeosaniwa canadensis*, and UMNH VP 16266 belongs to a different taxon, *Bolg amondol* gen. et sp. nov.

#### 4.7.2. Skull roof osteoderm comparisons

**Figure 42.**
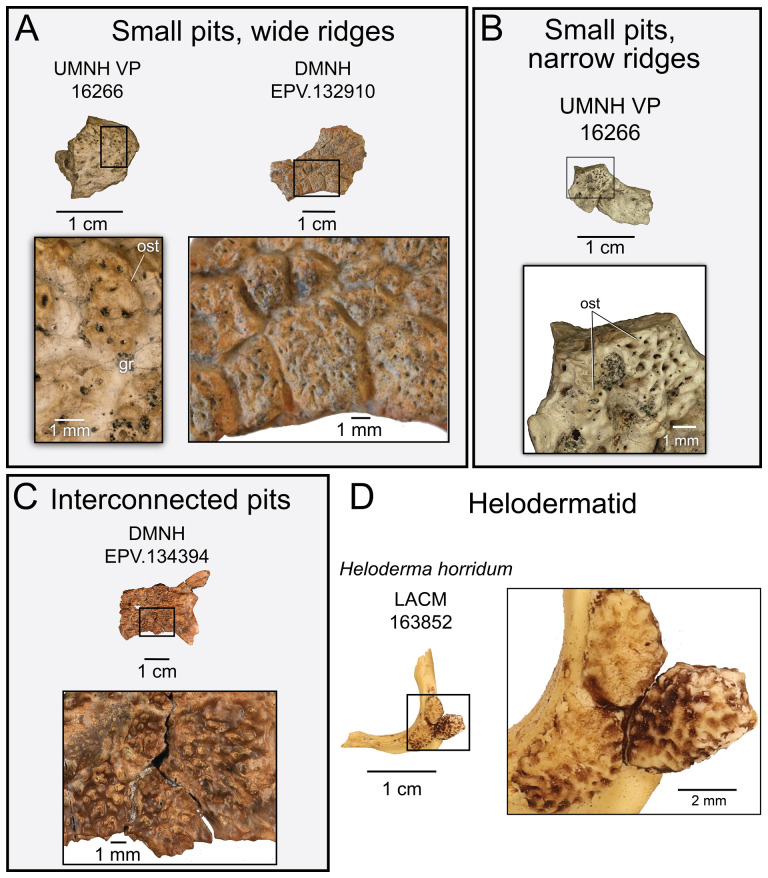
Comparisons of cranial dermal sculpturing on monstersaurian specimens. (A) ‘Small pits, wide ridges’ osteoderm morphotype, as seen on partial right maxilla UMNH VP 16266.2 (*Bolg amondol*), DMNH EPV.132910 (cf. *Palaeosaniwa canadensis*). (B) ‘Small pits, narrow ridges’ osteoderm morphotype, as observed on partial right jugal UMNH VP 16266.2 (*Bolg amondol*). (C) ‘Interconnected pits’ osteoderm morphotype, as observed in DMNH EPV.134394.1, partial parietal (cf. *Palaeosaniwa canadensis*). (D) ‘Helodermatid’ osteoderm morphotype, observed on LACM 163852 (*Heloderma horridum*).

UMNH VP 16266, DMNH EPV.134934.1 and DMNH EPV.132910 all preserve pitted polygonal osteoderms fused to the dorsal surface (figure 42). The osteoderms on each of these specimens exhibit morphological variation that is comparable to other Late Cretaceous monstersaurian specimens. In Late Cretaceous North America, we observe at least four distinct osteoderm morphotypes among described taxa (figure 42). The first osteoderm morphotype (osteoderm surface with wide ridges separating small pits; figure 42A) is the most widely observed among published monstersaur material, including on the maxilla of UMNH VP 16266, material attributed to *Paraderma bogerti* (Holotype maxilla UCMP 54261 [3,22], referred parietal TMP 1985.058.0058 [7]), and a monstersaurian frontal TMP 1978.008.0001 from the Campanian Oldman Formation of Alberta referred to cf. *Palaeosaniwa canadensis* [30]. The second osteoderm morphotype, characterized by an osteoderm surface with small pits separated by thin, septum-like ridges, is observed on the jugal of UMNH VP 16266 (figure 42B) and a fragmentary pair of frontals from the Campanian Oldman Formation of Alberta (TMP 1988.036.0212 [7]). The third osteoderm morphotype, which is denoted by a surface of interconnected pits separated by vermiculate ridges, is observed on the parietal DMNH EPV.134394.1 (figure 42C), as well as a frontal UALVP 59503 from the Campanian Wapiti Formation of Alberta [30]. A fourth morphotype observed in the Late Cretaceous of North America, characterized by dorsoventrally thin, wide plates with small pits that are widely spaced apart, is observed on the parietal 1985.058.0058 from the Campanian Oldman Formation of Alberta [7], and is a feature unique to that specimen. Different from all of the other osteoderm morphotypes is the ‘*Heloderma* morphotype’, which can be characterized by subcircular/subrounded vermiculate osteoderms that are not tightly fused to the skull roof, as observed in *Heloderma* (e.g. *H. horridum*; figure 42D).

The differences in osteoderm morphotypes present on the maxilla and jugal of UMNH VP 16266 (figure 42A,B) is consistent with recent observations of varying degrees of cephalic osteoderm morphology across different regions of the skull in extant individuals within Platynota/Varanoidea (e.g. *Varanus komodoensis*, *Lanthanotus borneensis*, *Heloderma suspectum*, *Varanus salvator* [85], figs 1 and 2; [20]) as well as differences in osteoderm size and shape on the frontals and parietals of extinct monstersaurs, such as *Gobiderma pulchrum* [12]. As a result, comparisons among overlapping elements of the skull among monstersaur taxa probably provides the most valuable insight on potential taxonomic differences in cephalic osteoderm morphology [30].

The osteoderms on the nasal process of the maxilla of UMNH VP 16266 (*Bolg amondol*) and the anterior nasal process of the holotype specimen of *Paraderma bogerti* UCMP 54261 belong to the same morphotype, and are difficult to distinguish from one another other than the slight difference of osteoderms near the dorsal margin of the nasal process are proportionally more fragmented on UMNH VP 16266 than on UCMP 54261. Osteoderms are notoriously variable in positioning and shape across intraspecifically and ontogenetically, sometimes fully absent at early ontogenetic stages [20,^84^,^85^]. Therefore, differences in relative size and position of maxillary osteoderms in *Par. bogerti* and *Bolg* are probably due to a multitude of factors beyond taxonomic difference.

The osteoderms fused to the parietals of DMNH EPV.132910 (figure 42A) and a parietal referred to *Par. bogerti* (TMP 1985.058.0058) [7] also belong to the same morphotype and are strikingly similar to those attached the partial frontal TMP 1978.008.0001 from the Campanian Oldman Formation of Alberta [7,30]. This suggests that groove-separated osteoderms with small pits, each separated by wide ridges between them, was a widespread anatomical feature in North American taxa throughout the Late Cretaceous, and may be a plesiomorphic trait, as evidenced by the presence of pitted osteoderms separated by grooves on the holotype maxilla and referred parietal of *Primaderma nessovi* [10].

Even though they are located on different skull elements, the osteoderms on the jugal of UMNH VP 16266 and the partial fused frontals (TMP 1988.036.0212 [7]), are similar. As discussed in the description of the jugal above, the presence of fused osteoderms is a unique feature among monstersaurs with preserved jugals. Additionally, the osteoderms on TMP 1988.036.0212 are notably different from those observed on TMP 1978.008.0001 [7,30] and UALVP 59503 [30].

The osteoderms fused to the parietal DMNH EPV.134394.1 (figure 42C) and UALVP 59503 [30] are also similar in that they possess interconnected pits with vermiculate ridges. This also appears to resemble those described on the frontal of MOR 792 (for an extensive discussion on these similarities and differences among frontals, see [30]).

**Figure 43.**
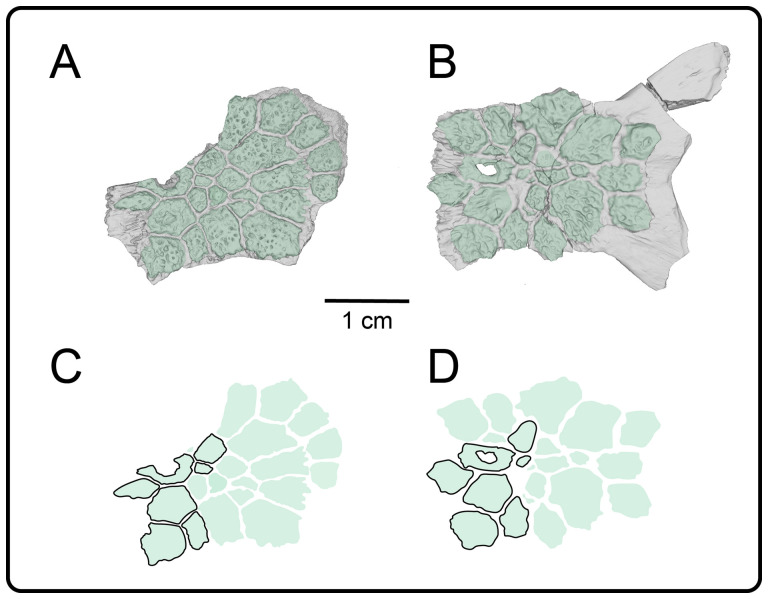
Comparisons of rendered CT scans of the dorsal surfaces DMNH EPV.132910 and DMNHEPV.134394.1. (A) Dorsal surface of DMNH EPV.132910, with osteoderms mapped in green. (B) Dorsal surface of DMNH EPV.134394.1, with osteoderms mapped in green. (C) Osteoderm patterning of DMNHEPV.132910. Osteoderms outlined in black indicate shared shapes/positions among the two parietals. (D) Osteoderm patterning of DMNH EPV.134394.1. Osteoderms outlined in black indicate shared shapes/positions among the two parietals.

**Figure 44.**
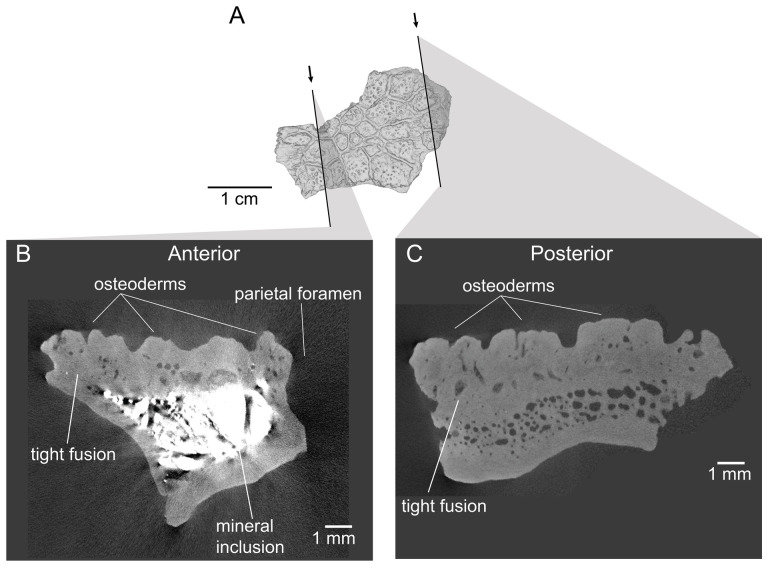
Comparisons of cranial dermal sculpturing on the parietal table of DMNH EPV.132910. (A) Rendered CT scan in dorsal view. Black lines and arrows indicate the positions and angles at which CT slices in (B) and (C) were taken. (B) Anterior slice of CT data. (C) Posterior slice of CT data.

**Figure 45.**
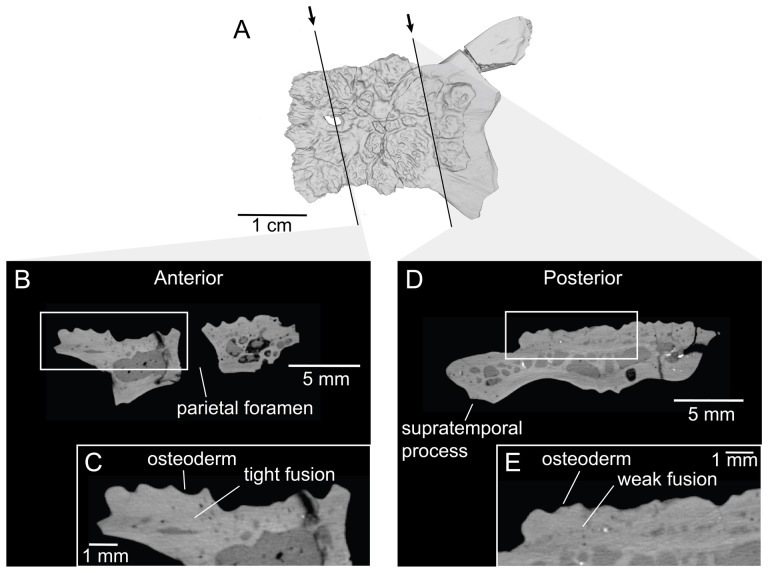
Comparisons of cranial dermal sculpturing on the parietal table of DMNH EPV.134394.1. (A) Rendered CT scan in dorsal view. Black lines and arrows indicate the positions and angles at which CT slices in (B–E) were taken. (B) Anterior slice of CT data. White box indicates position of zoomed-in image in (C). (C) Close-up image illustrating strong fusion of anterior osteoderms to the parietal table. (D) Posterior slice of CT data. White box indicates position of zoomed-in image in (E). (E) Close-up image illustrating weak fusion of anterior osteoderms to the parietal table.

Although DMNH EPV.134394.1 and DMNH EPV.132910 exhibit differing morphology of their individual osteoderms, they exhibit some similarities in the shape and patterning of the osteoderms on the anterior half of the parietal table (figure 43A,B). Particularly on the anterior, left-lateral side, both parietals appear to have similar positioning, size and shapes of osteoderms, including those surrounding the parietal foramen (figure 43C,D). Posteriorly, there are no obvious positional or shape correlates among the osteoderms in the two specimens, as there are different numbers of osteoderms of different sizes. Comparing differences in the osteoderm attachment on the anterior and posterior surfaces of both specimens (figures 44 and 45) reveals that anterior osteoderms on both specimens are strongly fused to the parietal table, whereas posterior osteoderms are not as tightly fused to the parietal table in DMNH EPV.134394.1 (figure 45D,E) as in DMNH EPV.132910 (figure 44C). This is evident in a clearly visible suture line on the posterior osteoderms in DMNH EPV.134394.1 (figure 45D,E). This suture line is not obviously visible in the anterior osteoderms of DMNH EPV.134394.1, nor the osteoderms in DMNH EPV.132910. This difference could be the result of several factors, chiefly including inter-/intras-specific variation and/or ontogeny (e.g. [71,84]).

#### 4.7.3. Parietal comparisons

##### Kaiparowits specimens

**Figure 46.**
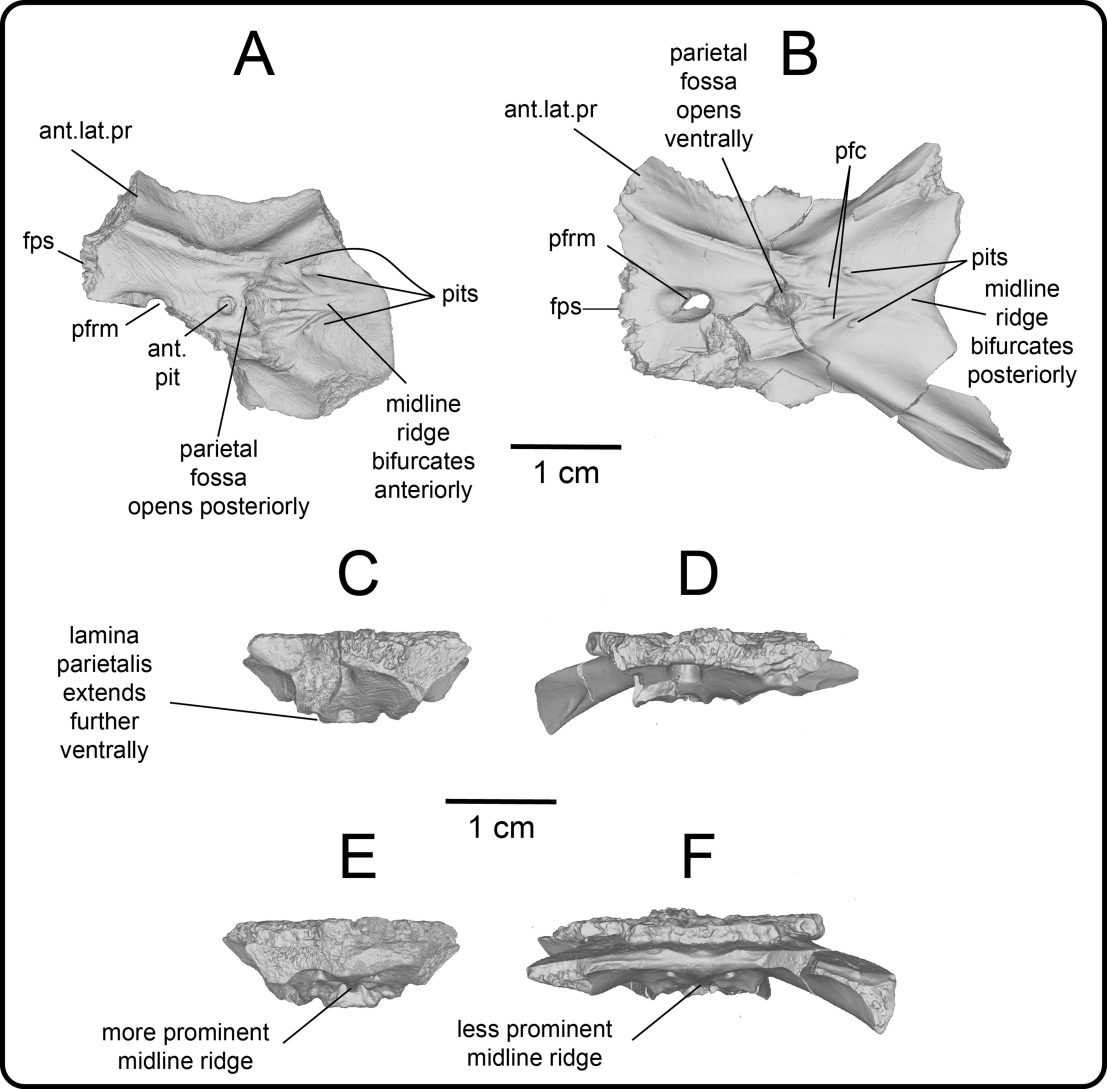
Comparisons of CT scans of DMNH EPV.132910 and DMNH EPV.134394.1. DMNH EPV.132910 in (A) ventral, (C) anterior and (E) posterior views. DMNH EPV.134394.1 in (B) ventral, (D) anterior and (F) posterior views. Anatomical abbreviations: ant.lat.pr, anterolateral process; ant. pit: anterior pit on the lamina parietalis; fps, frontoparietal suture; pfc, postfoveal crest; pfrm, parietal foramen.

Both parietals DMNH EPV.132910 and DMNH EPV.134394.1 (figure 46) exhibit similar general morphology including: (i) dermal sculpturing in the form of pitted polygonal mounds; (ii) co-fusion into a single element; (iii) presence of a parietal foramen; (iv) ventral attachment of jaw adductor musculature; and (v) supratemporal processes oriented posterolaterally. Additionally, both specimens possess a flat parietal table that extends to the posterior margin; correlation among similar structures (e.g.the convergence of the supratemporal processes on the parietal table; figure 46) show that the specimens are nearly identical in size; and the position/orientation of the parietal foramen is nearly identical. However, the ventral surfaces of DMNH EPV.134394.1 and DMNH EPV.132910 (figure 46) differ in a number of ways, including: (i) lamina parietalis of DMNH EPV.132910 extends ventrally to a greater extent than in DMNH EPV.134394.1 (figure 46C,D); (ii) parietal fossa of DMNH EPV.132910 opens posteriorly, whereas the parietal fossa of DMNH EPV.134394 opens ventrally (figure 46A,B); and (iii) DMNH EPV.132910 possesses a pit on the anteroventral margin of the lamina parietalis, absent in DMNH EPV.134394.1 (figure 46A–D). In terms of the different dermal sculpturing on the dorsal surface of the parietals, osteoderms can be variable in texture across a single individual (as discussed above), but also among individuals in anguimorph taxa [65,85,86] and helodermatids [16,18,20,87]. Finally, the observed differences between DMNH EPV.134394.1 and DMNH EPV.132910 could be the product of differences in ontogenetic stage. Major heterochronic differences have been observed both in external dermal sculpturing and in ventral morphologies of parietals at different ontogenetic stages in the extant anguid lizard genus *Ophisaurus* [65]. Further work is needed to understand the morphological variability of skull roof osteoderms in and in the parietals of monstersaur taxa,specifically, *Heloderma*. However, the presence of heterochronic variability in a closely related Monstersauria outgroup (according to genomics-based phylogenetic hypotheses; see §5) suggests that interspecific morphological variation across individuals and ontogeny is a likely explanation for the differences observed in the two parietals from the Kaiparowits Formation.

Given the general morphological similarities that DMNH EPV.134394.1 and DMNH EPV.132910 share, coupled with documented explanations for their potential subtle differences, there is not sufficient evidence to make taxonomic distinctions between the specimens. Until more complete and associated material is recovered, we hypothesize that the two parietals belong to the same taxon (cf. *P. canadensis*) and that the differences they exhibit are due to intraspecific variation in bone/osteoderm development and morphology.

##### Kaiparowits specimens and other monstersaurian parietals

**Figure 47.**
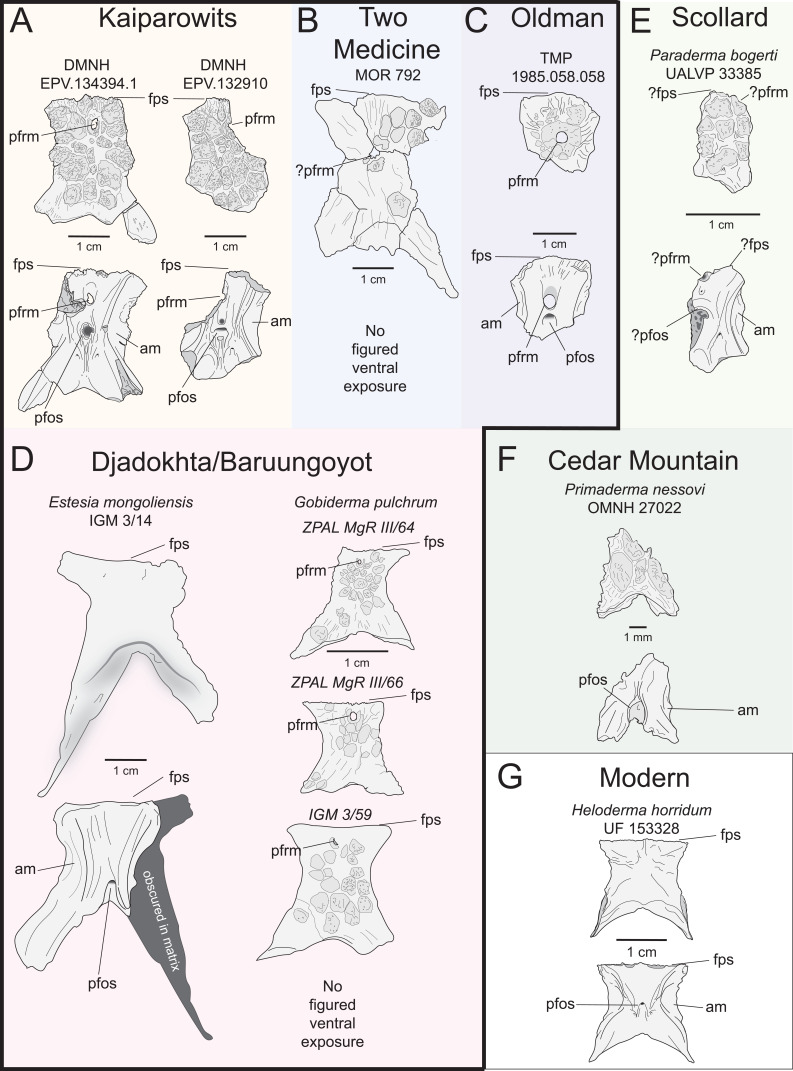
Line drawings comparing parietal morphology among Late Cretaceous and Modern monstersaurs. (A–D) Campanian monstersaur specimens, denoted by black border. (A) Monstersaur parietals from the upper Campanian Kaiparowits Formation (Fm) in dorsal (top) and ventral (bottom) views. (B) Dorsal view of the parietal from specimen MOR 792 (referred to *Palaeosaniwa canadensis*) from the Campanian Two Medicine Fm, modified from Balsai [8] and Hamilton *et al*. [30]. (C) Dorsal (top) and ventral (bottom) views of specimen TMP 1985.058.058 (Helodermatidae indet.) from the upper Campanian Oldman Fm, modified from Gao & Fox [7]. (D) Described monstersaur parietals from the Campanian Djadokhta/Baruungoyot formations. Left: Parietal from the holotype specimen IGM 3/14 of *Estesia mongoliensis* in dorsal (top) and ventral (bottom) views, modified from Norell *et al*. [5]. Right: dorsal views of parietals referred to *Gobiderma pulchrum*. Top: ZPAL MgR III/64 (holotype), modified from Conrad *et al*. [12]; middle: ZPAL MgR III/66, modified from Conrad *et al*. [12]; bottom: IGM 3/59, modified from Conrad *et al*. [12]. (E) Parietal UALVP 33385 from the Maastrichtian Scollard Fm, referred to *Paraderma bogerti*, in dorsal (top) and ventral (bottom) views. Modified from Gao & Fox [7]. (F) Parietal OMNH 27022 referred to *Primaderma nessovi* from the Cenomanian Mussentuchit Member, Cedar Mountain Fm, in dorsal (top) and ventral (bottom) views. Modified from Nydam [10]. (G) Parietal of UF 153328 (*Heloderma horridum*) in dorsal (top) and ventral (bottom) views. Anatomical abbreviations: am, adductor musculature attachment; fps, frontoparietal suture; pfos, parietal fossa; pfrm, parietal foramen.

The occurrence of other parietals with monstersaurian features (e.g. flat parietal table, ventral origin of adductor musculature) in the Late Cretaceous (figure 47A–F) allow us to compare DMNH EPV.134394.1 and DMNH EPV.132910 more broadly among related taxa. First, the Kaiparowits specimens, and particularly DMNH EPV.134394.1, share an exceedingly similar overall shape to the preserved parietal of MOR 792 (figure 47B) [8,30]. Both of the Kaiparowits specimens and MOR 792 share a medially curved margin of the supratemporal fenestra, which Balsai [8] noted contributes to an ‘hourglass-shaped’ parietal table. Balsai [8] noted a lack of evidence for a parietal foramen on MOR 792, although it is worth noting that the heavily fractured areas of the parietal converge on a hole that is conspicuously similar in location and size to the parietal foramina on other monstersaur parietals (figure 47A,C,D,G). A redescription of MOR 792 is ongoing (K. Smith & E. Metz 2023, personal communication to C.H.W.) and promises to reveal important information on the nature of this feature. If the parietal of MOR 792 does not have a parietal foramen, as surmised in Balsai [8], this could be a major morphological and evolutionary difference between the Two Medicine monstersaur represented by MOR 792 and the Kaiparowits monstersaur represented by DMNH EPV.132910 and DMNH EPV.134394.1. If the hole on MOR 792 is in fact a parietal foramen, it would be located further posterior on the parietal table than either parietal foramen on the Kaiparowits specimens, which are identically positioned relative to the frontoparietal suture (figure 47A). The identical positioning of the parietal foramen on both of the Kaiparowits specimens, as well as the identical positionings of the parietal foramen on three of the at least five parietals referred to *Gobiderma pulchrum* (figure 47D) [12] indicate that positioning of the parietal foramen is conserved across individuals of a given taxon. The relative angle that the supratemporal processes extend from the parietal table is similar in MOR 792 and DMNH EPV.134394.1 (figure 47A,B). No ventral view of MOR 792 is available in the published literature, and our comparisons are thus limited to the figured dorsal view.

TMP 1985.058.0058 (figure 47C) from the Campanian Oldman Formation, Alberta, is represented by the anterior half of the parietal table. Like the Kaiparowits specimens and MOR 792, the medial margin of the supratemporal fenestra is medially curved. The dorsal portion of the parietal table is partly covered by thin, platelike osteoderms, the largest of which surrounds the parietal foramen. The parietal foramen on this specimen is at least twice the diameter of those found on the Kaiparowits specimens, even though the parietals are similar in size. Additionally, the parietal foramen on TMP 1985.058.0058 is located further posteriorly from the frontoparietal suture than the Kaiparowits specimens. On the ventral side (figure 47C, bottom panel), the parietal foramen is located just anterior to the parietal fossa. Additionally, the ventral attachment for the adductor musculature on TMP 1985.058.0058 is mediolaterally narrower than those observed on the Kaiparowits specimens. The relative size and positioning of the parietal foramen, the narrower attachment surfaces for the adductor musculature and the unique osteoderm morphology on the dorsal surface of TMP 1985.058.0058 indicate significant morphological differences between the Oldman specimen and the Kaiparowits specimens.

Examples of described parietals from the Djadokhta/Baruungoyot (figure 47D) are figured to illustrate the diversity of parietal shape and size among Monstersauria. The parietal of *Estesia* differs from the Kaiparowits specimens in that it does not preserve a parietal foramen, the short axis of the supratemporal process is dorsoventrally oriented and blade-like, and there appears to be a prominent nuchal fossa dorsal view. Similar to the Kaiparowits specimens, the margin of the supratemporal fenestra is medially curved, such that the parietal table is hourglass-shaped in dorsal view, unlike the trapezoidal parietal of *Gobiderma pulchrum* and *Heloderma horridum* (figure 47G), and that inferred by Gao & Fox [7] for the parietal table for UALVP 33385, referred to *Paraderma bogerti* (figure 47E). Similar to the Kaiparowits specimens, *Gobiderma pulchrum* preserves a parietal foramen positioned just posterior to the frontoparietal suture, with posterolaterally oriented supratemporal processes that are flattened (figure 47D). Bead-like pitted osteoderms are present on the dorsal surfaces of the parietals [12].

UALVP 33385 is a partial parietal from the Maastrichtian Scollard Formation of Alberta referred to *Paraderma bogerti* [7] based on osteoderm morphology and comparable size to the holotype maxilla UCMP 54261 [3,7,22] (figure 47E). The osteoderms on UALVP 33385 are separated by deep grooves and have small pits with wide ridges separating them, like that observed in DMNH EPV.132910. Gao & Fox [7] note that the lateral margin of the parietal table is relatively straight, rather than medially concave, as seen in MOR 792 and the Kaiparowits specimens, and more similar to that seen on *Heloderma* (figure 47G), but the left-lateral margin of the parietal is incomplete anteriorly and posteriorly, thus making this trait difficult to assess fully. Gao & Fox [7] observed the presence of a partially preserved parietal foramen positioned nearly adjacent to the frontoparietal suture, which differs from the positioning observed in all other figured parietals with a parietal foramen in figure 47. Gao & Fox [7] mention the presence of a parietal fossa and that the parietal foramen is located more anteriorly on UALVP 33385 than on TMP 1985.058.0058 from the Oldman Formation (figure 47C) [7]. However, the feature is poorly preserved and its location was not explicitly discussed in the text. The figured specimens in the study by Gao & Fox [7] suggest that the parietal fossa is located in a position similar to that found in the Kaiparowits specimens, MOR 792 and TMP 1985.058.0058, located just posterior to the level of the apex of the medial curve of the anterior ramus of the *crista crania parietalis*. If the inferred placements of the described features of UALVP 33385 [7] are accurate, this could imply that the parietal foramen of the Maastrichtian UALVP 33385 (referred to *Paraderma bogerti*) is located further anterior to the parietal fossa than any of the Campanian monstersaur specimens. UALVP 33385 is also much smaller than the Campanian specimens, so it could be possible that these differences are ontogenetic. However, the consistency of the size of UALVP 33385 with other material referred to *P. bogerti* (All specimens are from the Maastrichtian [3,4,7,22]) suggests that *P. bogerti* achieved smaller body sizes than the Campanian monstersaurian taxa. Given the current information available, we interpret the differences in the positioning of the parietal foramen to be the result of taxonomically informative anatomical differences.

## 5. Phylogenetic analyses: data, methodology and results

### 5.1. Phylogenetic dataset

To estimate the evolutionary relationships of *Bolg amondol* among Anguimorpha, we modified the phylogenetic dataset from Conrad [73], updated by Bolet *et al*. [88], which includes one of the most extensive morphological character datasets to-date for squamates (836 characters). The full [73] dataset examines the relationships of all lepidosaur groups and their immediate outgroups (201 taxa), and is an aggregate of previously published matrices for osteological characters [11,12,24,25,71,89,90], soft tissue [89] and salivary compounds [90]. Because of the suite of morphological similarities that *B. amondol* shares with monstersaurs and other, closely related anguimorph taxa (e.g. *Varanus*, *Parasaniwa*), we removed all of the non-anguimorph squamate taxa from the Conrad [73] dataset and selected outgroup taxa identical to previously published versions of the Conrad matrix that focus on anguimorph ingroup relationships [6,14,24]. We added five taxa to this pruned down matrix, all of which are hypothesized to have monstersaurian affinities: *Asprosaurus bibongriensis* [14]; *Bolg amondol* (this study); *Chiangshia nankangensis* [13]; *Labrodioctes montanaensis* [7]; *Morohasaurus kamitakiensis* [15]. We scored these taxa based on published character scorings in the Conrad matrix [13,14], as well as published figures and anatomical descriptions. The resulting matrix includes 42 taxa. We also added three new characters pertaining to monstersaur taxa in the matrix. The characters are listed here:

**837**. maxilla, anterodorsal apex of the nasal process, bordering the external naris:
  (0) smooth
  (1) angulated 
**838**. dentary, shape, strength of ventral convexity of the subdental margin:
  (0) absent
  (1) weak
  (2) strong
**839**. dorsal vertebrae, centrum ventral surface:
  (0) flat
  (1) ovoid pit at the midline
  (2) large ventral depression

**Figure 48.**
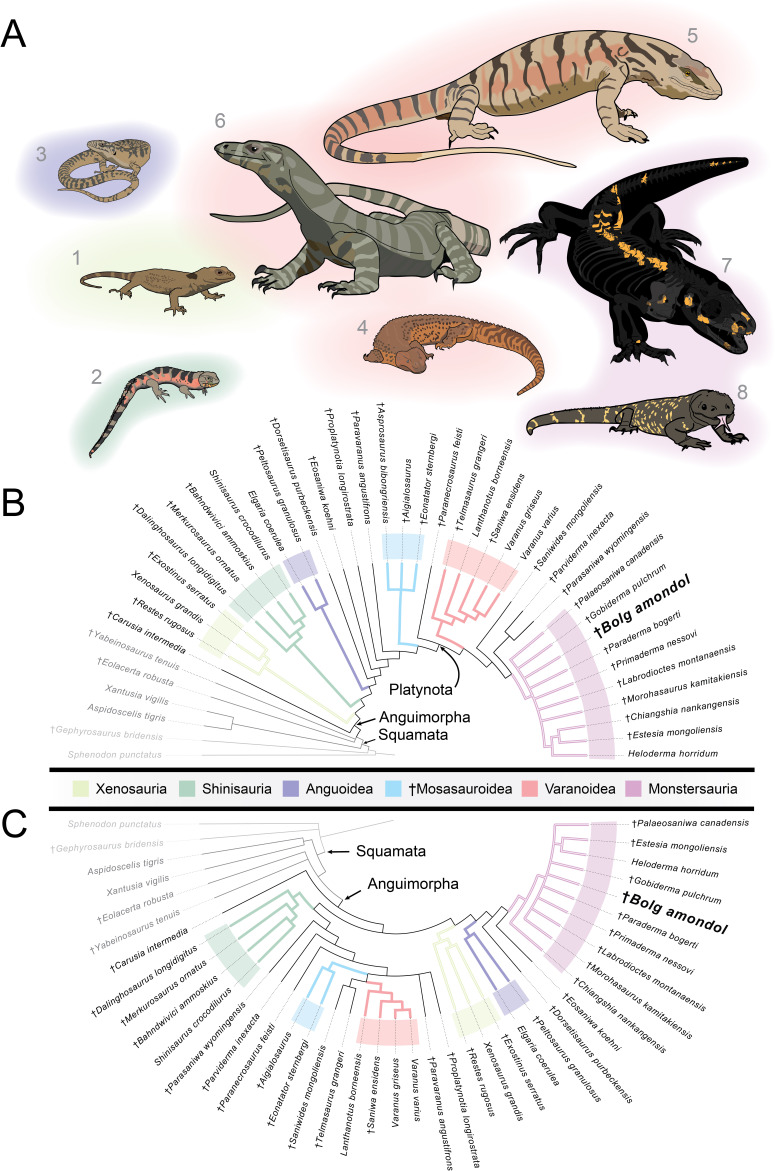
Phylogenetic relationships of *Bolg amondol* among Anguimorpha, using a modified version of the Conrad [73] matrix with the corrections of Bolet *et al*. [88]. Outgroup selection follows Conrad *et al*. [12] and Yi & Norell [6]. (A) Representative taxa from the five extant anguimorph clades (not to scale). (1) *Xenosaurus grandis*; (2) *Shinisaurus crocodilurus*; (3) *Elgaria coerulea*; (4) *Lanthanotus borneensis*; (5) *Varanus griseus*; (6) *Varanus varius*; (7) *Bolg amondol*; (8) *Heloderma horridum*. (B) Results of the unconstrained analysis of the phylogenetic relationships of *B. amondol*. Strict consensus of 52 most parsimonious trees shown. (C) Results from the constraint analysis using the genomics-based hypothesis of the phylogenetic relationships of Anguimorpha from Pyron *et al*. [26]. Strict consensus of 2016 most parsimonious trees shown. Extinct taxa in (B) and (C) denoted with the † symbol.

We carried out an unconstrained phylogenetic analysis using the software program TNT v. 1.6 [91]. In TNT, we allocated 10 GB of memory. We then uploaded the matrix as a .tnt file to the program. We instructed TNT to hold up to 1 000 000 trees in the memory using the 'hold' command. We specified *Sphenodon punctatus* as the outgroup taxon. We then deactivated 514 uninformative characters (list of deactivated characters is provided in electronic supplementary material S3) using the *xinact* command. With these parameters, we then carried out a New Technology Search (sectorial, ratchet, drift, and tree fusing options activated) to search for 500 minimum tree length recoveries. The analysis resulted in 52 most parsimonious trees (MPTs) with tree lengths of 973 steps (figure 48B; table 1). These trees and the matrix were then exported as a NEXUS file (.nex) and uploaded into PAUP* v. 4.0a169 [92] to compute consensus trees and calculate consistency and retention indices (table 1).

**Table 1.** Simplified results from phylogenetic analyses in this study. All analyses in the table only include the holotype specimen of *Bolg amondol* (UMNH VP 16266). Abbreviations: CI: consistency index; MPTs: most parsimonious trees; RI: retention index; PEA: Pyron et al. (2013).

analysis	MPTs	tree length	CI	RI	monophyletic monstersauria?
Conrad [73] Anguimorpha	52	973	0.431	0.589	Yes
PEA [26] genomics constraint	2016	1000	0.419	0.564	Yes

Additionally, to test the sensitivity of the relationships of *B. amondol* and the monophyly of Monstersauria under fundamentally different hypotheses of anguimorph relationships, we ran a constraint analyses in PAUP* v. 4.0a169 [92] using a genomics-based hypothesis of anguimorph evolutionary relationships [26]. For this analysis, we loaded the modified Conrad [73] matrix into PAUP* and then uploaded a backbone constraint .tre file with the topology of Pyron *et al*. [26], keeping only extant species as terminal taxa (electronic supplementary material, S6).The constraint analysis was carried out in PAUP* as follows: once the constraint tree file was uploaded using the ‘load constraints’ option under the ‘Analysis’ menu, we carried out a Heuristic Search, keeping all trees that aligned with the uploaded constraint topology. We deactivated the 514 uninformative characters and set the outgroup taxon to *Sphenodon punctatus*. We ran 500 replicates for each search, with a timer of 30 s set for each replicate to facilitate completion of analyses in a reasonable timeframe. Once the analyses were finished, we calculated consensus trees and exported the results as a NEXUS file (electronic supplementary material, S7). For this constraint analysis, we recovered 2016 MPTs of 1000 steps in length (figure 48C; table 1).

### 5.2. Phylogenetic relationships of *Bolg amondol* among Anguimorpha

The strict consensus trees of both the unconstrained morphology-only (figure 48B) and genomics-constrained (figure 48C) phylogenetic analyses illustrate *B. amondol* is recovered within a monophyletic Monstersauria. *Labrodioctes montanaensis* and *Morohasaurus kamitakiensis* are also recovered as monstersaurs in both analyses, and form a polytomy with *B. amondol*, *Paraderma bogerti*, *Palaeosaniwa canadensis*, *Primaderma nessovi*, and the monophyletic clade formed by *Estesia mongoliensis* and *Heloderma horridum. Chiangshia nankangensis* is also recovered within Monstersauria, but its positioning within the clade varies considerably between the morphology and genomics-based topologies. In the unconstrained analysis (figure 48B), *C. nankangensis* is recovered as the sister taxon to *E. mongoliensis*, reinforcing the initial hypothesis of its evolutionary relationships [13]. When enforcing the genomics-based hypothesis of anguimorph relationships (figure 48C), *C. nankangensis* is recovered as the outgroup taxon to the rest of Monstersauria.

*Asprosaurus bibongriensis* [14] is not recovered as a monstersaur, and instead is recovered in a clade with mosasauroids *Eonatator sternbergi* and *Aiglialosaurus* (figure 48C), forming the outgroup to Platynota. This is not necessarily surprising, as many portions of the skeleton that are used to diagnose Monstersauria (e.g. the parietal and marginal tooth-bearing bones) are either missing or only partially preserved in *As. bibongriensis*. Park *et al*. [14] highlight the uncertainty of this taxon’s phylogenetic position when utilizing the Gauthier *et al*. [25] dataset. Two hundred seventy one characters from the Gauthier *et al*. [25] analysis are included in the Conrad [73] dataset, which may partially explain the differences between the recovered relationships of *As. bibongriensis* in this study and that of Park *et al*. [14]. In fact, the affinities of *As. bibongriensis* are uncertain enough that, in the genomics constraint analysis in this study, it collapsed most of the anguimorph tree into a polytomy (electronic supplementary material). Thus, we removed *As. bibongriensis* from the final version of the genomics constraint analysis to elucidate the relationships of *B. amondol* among anguimorphs.

### 5.3. Synapomorphies of Monstersauria and close outgroups

In the unconstrained analysis (figure 48B), under both ACCTRAN and DELTRAN models of character optimization, Monstersauria is defined by the following combination of unambiguous synapomorphies: (i) Parietal tabs present on frontals (Char. 69 = 1); (ii) broad, flat dorsal margins of the parietal supratemporal processes (Char. 81 = 1); (iii) ventral attachment of jaw adductor musculature on the parietal (Char. 86 = 1); and (iv) distinct, elongate choanal groove on the palatine (Char. 113 = 1). Unfortunately, none of these characters could be scored for the current material assigned to *B. amondol*. Unambiguous synapomorphies that unite Monstersauria and its immediate outgroups in the unconstrained analysis (Most recent common ancestor of *Parasaniwa wyomingensis + Parviderma inexacta*; *Saniwides mongoliensis*; figure 48B) are as follows: (i**−**iii) presence of dermal sculpturing on the maxilla (Char. 8 = 1, scored for *B. amondol*), prefrontal (Char. 9 = 1), and frontal/parietal (Char. 10 = 1); (iv) Prefrontal does not block contact between nasal and maxilla (Char. 38 = 0); (v) Short and broad coronoid height relative to the rest of the mandible (Char. 192 = 0); (vi) short anterolateral extensions of the nasofrontal suture (Char. 378 = 0).

In the genomics constraint analysis (figure 48C), under both ACCTRAN and DELTRAN models of character optimization, Monstersauria is defined by the following combination of unambiguous synapomorphies: (i) Four or more maxillary/premaxillary tooth positions anterior to the septomaxilla (Char. 2 = 1); (ii) presence of cranial osteoderms that develop as thickened polygonal mounds (Char. 308 = 2, scored for *B. amondol*) with (iii) pitted sculpturing (Char. 7 = 1, scored for *B. amondol*). Unambiguous synapomorphies that unite Monstersauria and its immediate outgroups in the genomics constrained analysis (*Eosaniwa koehni*; *Dorsetisaurus purbeckensis*; figure 48C), exclusive to Anguoidea are as follows: (i) absence of medial flaring on the palatine flange of the maxilla (Char. 32 = 0); (ii) frontals paired and separate in adults (Char. 55 = 0); (iii) presence of parietal tabs on the frontals (Char. 69 = 1); (iv) anteromedial walling of the Meckel’s canal by the splenial extends less than One-half of the dentary length (Char. 189 = 0); (v) presence of a long, low anterior process on the coronoid (Char. 196 = 1). Unambiguous synapomorphies that unite Monstersauria with *Eo. koehni* exclusive to *D. purbeckensis* are as follows: (i) posterior extent of maxillary tooth row terminates at anterior border of orbit in lateral view (Char. 33 = 1); (ii) palatine roughly subequal in length and width in palatal view (Char. 111 = 1); (iii) absence of a posterodorsal coronoid process on the dentary (Char. 184 = 2); (iv) anterior end of coronoid abuts the dentary (Char. 194 = 1); (v) widely spaced dentition, spaces between tooth bases greater than one-half the width of a tooth shaft (Char. 211 = 1); (vi) trenchant, curved teeth (Char. 212 = 2).

Recovered ingroup taxa of Monstersauria include *B. amondol*, *G. pulchrum*, *L. montanaensis*, *M. kamitakiensis*, *Pr. nessovi*, *Paraderma bogerti*, *Pal. canadensis*, *E. mongoliensis*, *C. nankangensis* and *H. horridum*, as these taxa consistently plotted in a monophyletic clade in both the unconstrained (figure 48B) and constrained (figure 48C) analyses, even though the positioning of *C. nankangensis* differs between the two. Conrad *et al*. [12] included *Parv. inexacta* and *Parasaniwa wyomingensis* as the earliest branching lineages within Monstersauria, as our unconstrained analysis shows (figure 48B), but the placement of these taxa as outgroups to Varanoidea in the genomics constraint analysis (figure 48C) suggest considerable uncertainty in their systematic affinities. Therefore, we do not include *Parasaniwa wyomingensis,* nor *Parv. inexacta*, as ingroup taxa in Monstersauria.

### 5.4. Phylogenetic position of *Bolg amondol* among Monstersauria

The listed unambiguous synapomorphies of Monstersauria in both phylogenetic analyses above (figure 48) suggest that *B. amondol* is recovered within the clade based on a combination of osteoderm characters. The presence of these dermal characters in *B. amondol* are particularly important in distinguishing the taxon from varanoids, which lack dermal sculpturing on skull bones, even though osteoderms have been found to be present in the dermis of some taxa (e.g. *Varanus komodoensis* [85]). In addition to these unambiguous dermal synapomorphies, *B. amondol* shares numerous character states with monstersaur taxa that help explain its placement in the clade: (i) modified pleurodont marginal tooth attachment (Char. 214 = 2); (ii) presence of plicidentine in marginal teeth (Char. 218 = 1); (iii) absence of precondylar constriction on the dorsal vertebrae (Char. 233 = 0); (iv) caudal chevrons positioned at the posteroventral margin of the centrum (Char. 252 = 0); (v) presence of grooves that separate osteoderms on the maxilla (Char. 307 = 1; also observed in *Par. wyomingensis*). In both analyses, *B. amondol* preserves one unambiguous autapomorphy: the presence of fused osteoderms on the postorbital process of the jugal (Char. 837 = 1). Although no distal caudal vertebrae are described for other monstersaur taxa beside *Heloderma*, the presence of autotomy septa on the distal caudal vertebrae of *B. amondol* (Char. 250 = 0) represents the first described occurrence of the trait in the clade (autotomy planes are absent in *Heloderma*).

**Figure 49.**
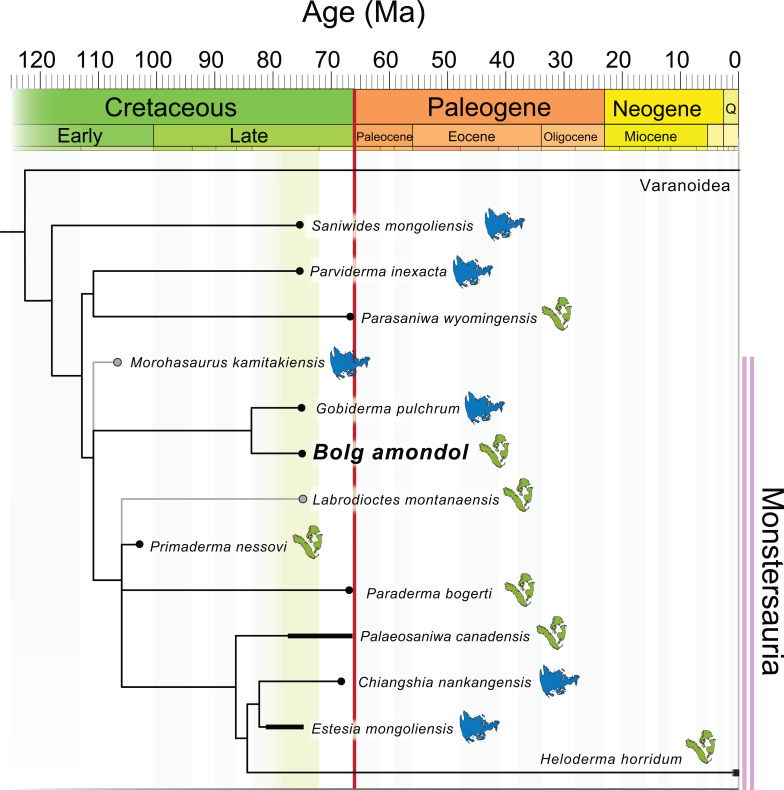
Adams consensus showing the relationships of Monstersauria and proximal anguimorph outgroups from the morphology-only analysis (figure 48B), time-calibrated according to Borsuk-Byalinicka [2], Gao & Fox [7], Conrad *et al*. [12], Mo *et al*. [13], Ikeda *et al*. [15] and radiometric dates for *Bolg amondol* [49,53]. Branches in grey indicate volatile taxa (*Morohasaurus kamitakiensis*, *Labrodioctes montanaensis*). Blue silhouettes: palaeogeographic map of late Campanian Asia, indicating Asian origin of taxon. Green silhouettes: palaeogeographic map of late Campanian North America, indicating a North American origin of taxon. Green vertical bar indicates the late Campanian Stage.

The strict consensus of both the unconstrained and constrained phylogenetic analyses recover *B. amondol* in a polytomy with *G. pulchrum*, *L. montanaensis*, *Pal. canadensis*, *Paraderma bogerti*, *Pr. nessovi*, *M. kamitakiensis* and the monophyletic clade formed by *H. horridum* and *E. mongoliensis*. To elucidate any potential structure to the monstersaur tree, we chose to also display the Adams consensus of the unconstrained morphological analysis, time-calibrated to illustrate temporal occurrences of terminal taxa in the group (figure 49). We emphasize here that we are not interpreting the timing of cladogenic events using the Adams consensus. The Adams consensus displays relationships that are common to all recovered trees, while collapsing volatile taxa to their least inclusive node [93]. In figure 49, we identify volatile taxa in grey (*M. kamitakiensis*, *L. montanaensis*). What is most likely to explain the volatile phylogenetic placement of these two taxa is that their published record to date only includes isolated dentaries [7,15,30], and the limited morphological information preserved in *M. kamitakiensis* and *L. montanaensis* leads to higher uncertainty in their recovered relationships. In 33 of 52 MPTs, *M. kamitakiensis* is recovered as nested within the clade *Pal. canadensis *+ the monophyletic clade formed by *H. horridum*, *E. mongoliensis* and *C. nankangensis*. However, *M. kamitakiensis* is also placed on various other branches of the monstersaur tree, including as its earliest diverging member (8 of 52 MPTs). *L. montanaensis* consistently plots out on lower levels of the monstersaur tree than *M. kamitakiensis*, and is most frequently recovered as the outgroup taxon to the clade *H. horridum*, *E. mongoliensis* and *C. nankangensis* (28 of 52 MPTs) or as sister taxon to *Paraderma bogerti* (20 of 52 MPTs).

In the Adams consensus, we observe that *B. amondol* is recovered as sister taxon to *G. pulchrum* and this clade forms a trichotomy with *M. kamitakiensis* and the most recent common ancestor to other monstersaurs, which form another monophyletic clade that includes the other volatile taxon, *L. montanaensis*. The major character that separates this clade from other monstersaurs is the presence of venom grooves in the marginal teeth (Char. 220 = 1). The teeth of *G. pulchrum* and *M. kamitakiensis* do not possess venom grooves [12,15]. The teeth of *B. amondol* are not fully preserved, and thus this trait is impossible to assess in this taxon at present. An isolated platynotan tooth from the Kaiparowits Formation with a conspicuous venom groove was described in Nydam [32], but it cannot be confidently assigned to *B. amondol* nor any other taxon. Thus, the ‘absence’ of a venom groove in *B. amondol* is due to missing anatomical data, and discovery of more complete material could shift the recovered relationships in this study (figure 49). Of note also is the frequent sister taxon relationships between Asian taxa and North American taxa (e.g. *Parviderma + Parasaniwa*; *Gobiderma + Bolg*; the most recent common ancestor of *Estesia* and *Chiangshia + Heloderma*). This pattern is consistent with the multiple biogeographic dispersal events between Asia and North America hypothesized for many non-avian dinosaur groups [43,52,94–99] and plant lineages [100] (see §6 below) during the Cretaceous.

## 6. Discussion

### 6.1. Reconsidering historical referrals to *Palaeosaniwa canadensis* (Gilmore, 1928)

With a few exceptions historically [7], much of the large anguimorph lizard material found in the Campanian of North America was referred to *Palaeosaniwa canadensis* [3,4,7,8,30,31,39]. Gilmore [31] erected the taxon *Pal. canadensis* on the basis of three isolated dorsal vertebrae (UALVP 112; USNM 10864, CMNFV 8510 (formerly GSC 8510)) from the Campanian Belly River Group of Alberta and ‘provisionally’ referred a posterior dorsal vertebra from the Maastrichtian Lance Formation (USNM 11045) to the species. In comparing these vertebrae to those of the Eocene genus *Saniwa,* Gilmore [31] observed that the type specimen (USNM 10864) exhibits: (i) relatively larger zygapophyses; and (ii) a median channel-like longitudinal depression (i.e. a ‘pit’) that traverses the ventral surface of the centrum. The latter character trait was also observed on specimen MOR 792, and was used in an unpublished PhD dissertation [8] to tentatively assign MOR 792 to *Pal. canadensis*. This median pit is also observed on DMNH EPV.134934 (cf. *Pal. canadensis*), but is not present on the vertebrae of UMNH VP 16266 (*B. amondol*).

The similarities between the vertebrae of DMNH EPV.134394 and MOR 792, and either the major differences (e.g. MOR 792 does not possess a parietal foramen) or minor but significant differences (e.g. the broken parietal foramen is located more posteriorly on parietal table in MOR 792 than in Kaiparowits specimens) in parietal morphology between the two specimens (see §4 above), invite us to reconsider the medial pit on the ventral centrum surface as autapomorphic for *Pal. canadensis*. Hamilton *et al*. [30] highlighted that a similar median groove/pit is present in other squamates, including extant species of *Varanus*, some mosasauroids and a vertebra assigned to *Parasaniwa wyomingensis* [7], but the combination of that character, as well as enlarged zygapophyses, large specimen size and the presence of moderate precondylar constriction currently remain diagnostic for the taxon until a demonstrably different taxon from MOR 792 (and USNM 10864) is described with grooved/pitted vertebrae. Here, the admittedly fragmentary specimen DMNH EPV.134394, which possesses a ventral medial pit and is large in size, suggests that either (i) the presence/absence or positioning of the parietal foramen was highly variable across individuals of *P. canadensis* or (ii) that multiple North American monstersaur taxa possessed a median groove/pit on the ventral surface of dorsal vertebral centra. This problematic juxtaposition indicates a glaring need for a full reassessment of *Palaeosaniwa canadensis* and other materials assigned to the taxon over the past century [3,4,7,8,30,31,39,101], but is far beyond the scope of the present study. At the very least, our results show that fossil elements of large lizards from the Late Cretaceous of North America should not uniformly be referred to *Pal. canadensis*, as was previously common practice.

### 6.2. Monstersaur diversity in the Kaiparowits Formation

**Figure 50.**
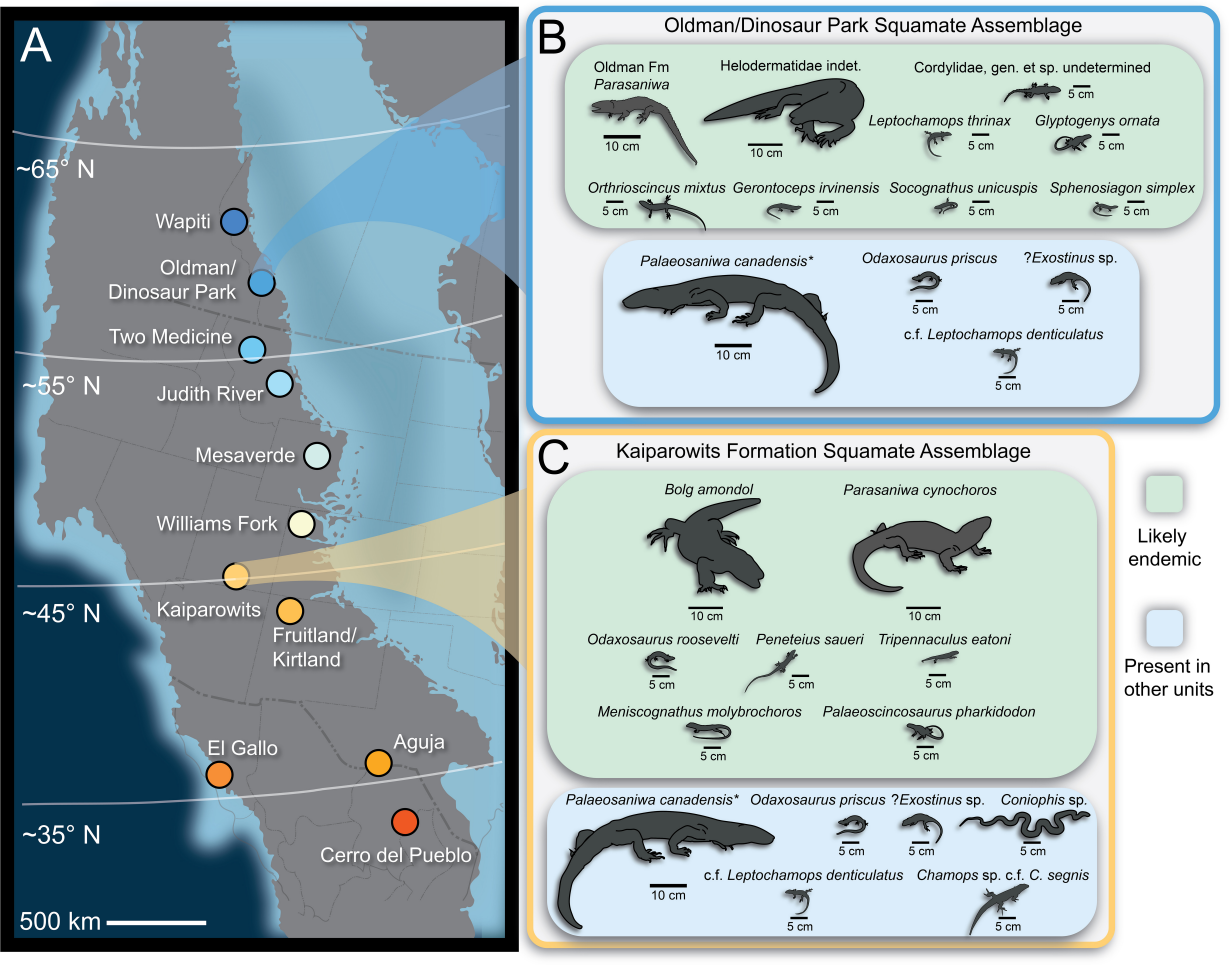
Taxonomic comparison of the two best-sampled squamate assemblages from the late Campanian of Laramidia. (A) Geographic distribution of published squamate occurrences in 11 penecontemporaneous depositional basins in the late Campanian of western North America, with modern political boundaries overlain. Modified from Sampson *et al*. [43]. Data reported from following references, listed by corresponding geologic unit, from north to south: Wapiti [30,102]; Oldman /Dinosaur Park [7,31]; Two Medicine [8,30,103]; Judith River [7,37]; Mesaverde [38]; Williams Fork [104]; Kaiparowits [32,41] (this study); Fruitland/Kirtland [36]; Aguja [34]; Cerro del Pueblo [39]; El Gallo [41]. (B) Published named taxa from the Oldman/Dinosaur Park formations in southeastern Alberta. (C) Published named taxa from the Kaiparowits Formation in southern Utah. Taxa in green boxes indicate probable endemic taxa, while taxa in blue boxes indicate taxa present in other geologic units. Silhouettes of lizard taxa modified from publicly available images of closest related modern squamate relatives at Phylopic.org. Body size of each taxon is a rough estimate based on published figures of described specimens. Asterisk next to *Palaeosaniwa canadensis* indicates uncertainty of affinity of material referred to the taxon.

Within the Kaiparowits Formation squamate assemblage, the differences in the described dorsal vertebrae of UMNH VP 16266 (*Bolg amondol*) and DMNH EPV.134394 (cf. *Palaeosaniwa canadensis*) reveal the presence of at least two co-occurring species of large-bodied monstersaurs in the same assemblage. Coupled with the incompletely known but distinct *Parasaniwa cynochoros* [32], the Kaiparowits Formation palaeoecosystem from the primary fossil-bearing horizons of the middle unit accommodated at least three, large-bodied anguimorph taxa (figure 50B). Based on comparable cranial material from other Campanian taxa (see §4), the large-bodied anguimorph Kaiparowits assemblage is likely made up of taxa both endemic to the depositional basin (e.g. *Parasaniwa cynochoros*, *B. amondol*) and those that are found in other units (*Palaeosaniwa canadensis*, pending recovery of more complete material and taxonomic reassessment). We make this hypothesis based on the well-documented evidence of regional faunal provinces on Laramidia [43,94,98,105,106], as well as: (i) previous assessments of endemic species of other lizard taxa in the Kaiparowits Formation [32,33,42,107]; (ii) assessments of endemic taxa in other squamate assemblages from syndepositional basins, such as the Oldman/Dinosaur Park formations [7], Wapiti Formation [30,102], Two Medicine Formation [103] and Aguja Formation [34]; (iii) unique occurrences of species of other tetrapod groups [94], such as non-avian dinosaurs [43,52,98,108]; and (iv) an endemic floral assemblage in the Kaiparowits Formation [44], illustrating varying levels of endemism across trophic levels within late Campanian Laramidia.

The anguimorph assemblage in the Kaiparowits Basin lends additional insight into ecological dynamics within known squamate assemblages of Laramidia and, in the best-sampled assemblages (figure 50), the accommodation of multiple large-bodied, faunivorous lizard species. Nydam [32] hypothesized that the blunted tooth crowns of *Parasaniwa cynochoros* indicate a durophagous component to the species’s diet, similar to modern *Varanus niloticus* [109] and *Dracaena guianensis* [110]. Although tooth crown morphologies are unknown for *B. amondol*, the modified pleurodont tooth attachment and basal enamel infoldings/plicidentine is consistently observed in extant faunivorous lizards, which include species in Varanoidea (e.g. *Varanus salvador*, *Lanthanotus borneensis* [6]) and *Heloderma* [6,19]. To our knowledge, no predominantly herbivorous lizard possesses the combination of a modified pleurodont tooth attachment and basal enamel infoldings/plicidentine in the tooth bases. Therefore, we believe it is reasonable to infer a predominantly faunivorous diet for *B. amondol*. The large body sizes for *Parasaniwa cynochoros*, *Bolg amondol* and cf. *Palaeosaniwa canadensis* suggest that they could target larger, more resource-rich prey than most other squamates in the ecosystem [111] but would have feasibly competed for resources with other faunivorous tetrapod taxa in the Kaiparowits assemblage, potentially including but not limited to small avian and non-avian theropod dinosaurs and crocodyliforms.

The co-occurrence of three large-bodied anguimorph squamates in the Kaiparowits Formation suggests a productive and stable ecosystem (as does the co-occurrence of many species of large-bodied dinosaurian herbivores), given the likelihood of competition between these species and with other vertebrates of similar body size. However, it is important to note that diet is only one factor that controls lizard diversity in an ecosystem. In modern lizard communities, the diet of closely related species may overlap considerably, but species will partition the habitat both spatially and temporally [112–115]. Thus, in addition to potential prey differences, the three large-bodied anguimorph taxa of the Kaiparowits ecosystem may also favour different microhabitats and/or activity periods. And it is important to emphasize that the co-occurrence of several closely related large-bodied anguimorphs is not unprecedented in modern ecosystems. For example, the Great Victoria Desert of western Australia is one of the most squamate-rich ecosystems in the world (>40 species) despite relatively low primary productivity [115,116]. Here, there are three species of *Varanus* with body lengths over half a metre with similar diets but differing in both relative abundance and spatial and temporal use of microhabitats [115]. Thus, it is reasonable to infer that Kaiparowits anguimorphs coexisted similarly even if they competed for many of the same prey species.

The presence of autotomy septa on the distal caudal vertebrae of *B. amondol* showcase two noteworthy anti-predator adaptations: dermal armour and voluntary detachment of the tail. Modern *Heloderma* (as well as *Varanus*) notably lack autotomy septa on their caudal vertebrae [77–79]. This may reflect infrequent predation on these larger-bodied lizard taxa in modern ecosystems and a lack of consistent need to possess a physiologically costly anti-predation adaptation [77]. While the full taxonomic and functional diversity of predators in the Kaiparowits palaeoecosystem is incompletely known, the presence of multiple anti-predation adaptations in *B. amondol* clearly suggest that having multiple deterrents may have been advantageous for large-bodied lizards in Late Cretaceous dinosaur ecosystems, which stands in clear contrast with the ecosystems they inhabit today.

### 6.3. An emerging portrait of the apex of monstersaur diversity

**Figure 51.**
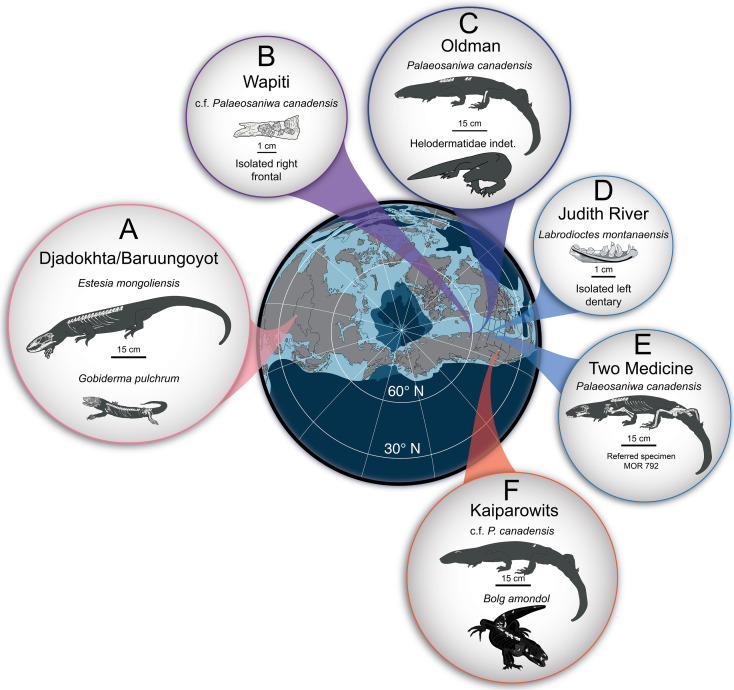
Geographic distribution of material assigned to monstersaurian taxa during the Campanian. Centre: Map of late Campanian Earth (ca. 76 Ma) centred on the North Pole. Modified from Scotese [117], which was rendered using Gplates software [118]. (A) Monstersaurian components of the squamate fauna of the Djadokhta/Baruungoyot formations, Mongolia. Reconstructions modified from Conrad *et al*. [12]. (B) Monstersaurian components of the squamate fauna of the Wapiti Formation, Alberta, Canada, modified from Hamilton *et al*. [30]. (C) Monstersaurian components of the squamate fauna of the Oldman Formation, Alberta, Canada [7,30]. (D) Monstersaurian components of the squamate fauna of the Judith River Formation [7]. (E) Monsterf ventral convexity of the subdentasaurian components of the squamate fauna of the Two Medicine Formation [8,30]. (F) Monstersaurian components of the squamate fauna of the Kaiparowits Formation (this study).

The presence of multiple species of monstersaurs in basins across the upper Campanian Western Interior of North America, as well as in the upper Campanian Djadokhta/Baruungoyot formations in Mongolia and China [9,35] bolsters hypotheses of floral and faunal biogeographic connections and interchanges between North America and Asia during the Cretaceous (figure 51). Faunal interchange has been hypothesized for many non-avian dinosaur groups, including ceratopsians [43,95,99], hadrosaurids [94,96], ankylosaurians [52] and tyrannosauroids [97,98], as well as in *Metasequoia, Sequoia* and several angiosperm lineages [100]. The timing of these interchange events has not been well established, though a late Early Cretaceous (Aptian–Albian) interchange has long been hypothesized [119–123], and multiple Late Cretaceous interchange events have been suggested [52,98]. Described fossils from Asia (*Morohasaurus kamitakiensis*, lower Albian Ohyamashimo Formation, Japan [15]) and North America (*Primaderma nessovi*, Cenomanian Mussentuchit Member, Cedar Mountain Formation [10]), suggest that monstersaurian lizards had been present on both Asia and western North America for at least 30 million years prior to the late Campanian, possibly a result of an earlier Aptian–Albian interchange.

Despite this increased global generic richness, monstersaurs make up a small proportion of the total taxonomic richness among their respective squamate assemblages [7,9,32,33] (figure 50). At the same time, across late Campanian assemblages, monstersaur taxa are the largest carnivorous lizards described from each ecosystem. In fact, this pattern persists through the Cretaceous–Palaeogene boundary and to the extant *Heloderma* ecosystems in the southwestern United States and northern Mexico [23]. Future work is needed to uncover the ecological and evolutionary pressures behind this pattern through time, but the addition of *Bolg amondol* and a second taxon, cf. *P. canadensis*, from the Kaiparowits Formation presents further evidence that monstersaurs have maintained similar roles as large-bodied predators in their ecosystems for at least 76 million years [5,8,10,23].

## 7. Conclusions

By describing the monstersaur assemblage of the Kaiparowits Formation, including a new taxon, *Bolg amondol*, we add crucial new evidence to the understanding of the evolutionary history of a charismatic group of lizards, represented today by five species of *Heloderma*. The Kaiparowits material offers insight into the morphological diversity of Monstersauria at the greatest extent of their global distribution during the late Campanian, approximately 77−74 million years ago. The presence of autotomy septa in the caudal vertebrae of *B. amondol* indicate that this adaptation was an ancestral trait that was subsequently lost in more recent taxa, such as extant *Heloderma*. Importantly, *B. amondol* is morphologically distinct from *Palaeosaniwa canadensis*, whereas other Kaiparowits monstersaur material that compares favourably to *Pal. canadensis* illustrates a glaring need for a reassessment of a genus that has remained problematic for nearly a century. The described material from the Kaiparowits Formation offers a clearer picture of monstersaurian richness during a crucial period of their evolutionary history. Critically, the Kaiparowits monstersaur material demonstrates the value of continuing to scrutinize historical collections and build new collections in the effort to uncover hidden biodiversity in a fragmentary fossil record.

## Data Availability

All CT data files will be uploaded to Morphosource.org and will be available upon acceptance of publication. All data used for phylogenetic analyses is included in the electronic supplementary material [124].
